# Scaffold Hopping Toward Agomelatine: Novel 3, 4-Dihydroisoquinoline Compounds as Potential Antidepressant Agents

**DOI:** 10.1038/srep34711

**Published:** 2016-10-04

**Authors:** Yang Yang, Wei Ang, Haiyue Long, Ying Chang, Zicheng Li, Liangxue Zhou, Tao Yang, Yong Deng, Youfu Luo

**Affiliations:** 1 State Key Laboratory of Biotherapy and Department of Neurosurgery/Collaborative Innovation Center for Biotherapy, West China Hospital, West China Medical School, Sichuan University, Chengdu, Sichuan 610041, P.R. China; 2 Key Laboratory of Drug Targeting and Drug Delivery System, Ministry of Education, West China School of Pharmacy, Sichuan University, Chengdu, Sichuan 610041, P. R. China; 3 Department of Pharmaceutical and Bioengineering, School of Chemical Engineering, Sichuan University, Chengdu, Sichuan 610065, P. R. China

## Abstract

A scaffold-hopping strategy toward Agomelatine based on *in silico* screening and knowledge analysis was employed to design novel antidepressant agents. A series of 3, 4-dihydroisoquinoline compounds were selected for chemical synthesis and biological assessment. Three compounds (**6a-1**, **6a-2**, **6a-9**) demonstrated protective effects on corticosterone-induced lesion of PC12 cells. Compound **6a-1** also displayed low inhibitory effects on the growth of HEK293 and L02 normal cells and it was further evaluated for its potential antidepressant effects *in vivo*. The forced swim test (FST) results revealed that compound **6a-1** remarkably reduced the immobility time of rats and the open field test (OFT) results indicated a better general locomotor activity of the rats treated with compound **6a-1** than those with Agomelatine or Fluoxetine. Mechanism studies implied that compound **6a-1** can significantly reduce PC12 cell apoptosis by up-regulation of GSH and down-regulation of ROS in corticosterone-induced lesion of PC12 cells. Meanwhile, the down-regulation of calcium ion concentration and up-regulation of BDNF level in PC12 cells may account for the neuroprotective effects. Furthermore, compound **6a-1** can increase cell survival and cell proliferation, promote cell maturation in the rat hippocampus after chronic treatment. The acute toxicity data *in vivo* indicated compound **6a-1** exhibited less hepatotoxicity than Agomelatine.

Depression characterized by sadness, loss of interest or pleasure, low self-esteem, poor concentration and always associated with disturbed sleep and appetite, affects approximately 350 million people all over the world^1^,^2^. According to the World Health Organization’s Global Burden of Disease project, major depressive disorder will become the leading cause of disability and a major contributor to the overall disease burden worldwide. Patients with major depression have an increased onset risk of aging-related somatic diseases such as heart disease, diabetes, obesity and cancer^3^,^4^. At its worst, depression can lead to suicide. Over 800 000 people die due to suicide every year and more than 70 percent suicides suffer from major depression^1^,^2^. Currently, most medications for treatment of depression target serotonergic and/or noradrenergic transmitter systems or inhibit monoamine oxidase to reduce the degradation of serotonin and noradrenaline. Despite that a large number of antidepressant drugs commercially available, there are still many issues leading to risks of depression therapy. It was reported that a number of patients who took antidepressant drugs experienced serious side effects and drug-drug interactions, with fewer than half of patients responding well to currently available treatments^5^. Besides, the long-lasting therapy period gives rise to poor patient compliance^6^.

The monoamine hypothesis of depression has dominated thinking about mood disorders since 50 years ago owing to the fact that both monoamine oxidase inhibitors and tricyclic antidepressants increased brain levels of monoamines. However, rapid drug-induced elevations of monoamine levels and symptom improvement require weeks of antidepressant treatment^7^. Neuroscientists have made great efforts to investigate the neurobiological and structural changes correlated with the clinical course over the last decade. Neuronal plasticity, neurogenesis in the adult brain, and the ability of antidepressants to regulate the expression of genes related to plasticity and resilience, have attracted great amount of attention in the past years^8^,^9^,^10^,^11^,^12^,^13^.

Several studies^14^,^15^,^16^,^17^ showed that hippocampal volume decreased in patients with stress-related major depression, which might be due to glial and neuronal atrophy or loss related in part to increases in corticosteroids and excitatory amino acids; such relationships have been demonstrated in animal models^18^,^19^,^20^, while still under investigation in humans^21^,^22^. Meanwhile, Agomelatine, a recently marketed antidepressant drug, was reported to induce neurogenesis and cell proliferation in the ventral part of dentate gyrus, resulting to the rapid and early increase in maturation at a critical period of neuronal development, which likely influences the functional integration of new born cells into hippocampal circuitry. The mentioned above formed the basis for the neuroplasticity hypothesis of major depression. Fluoxetine and many other antidepressants in clinic also shared above neurogenetic effects^23^,^24^,^25^,^26^,^27^,^28^. In addition, many studies indicated that antidepressant drugs are able to prevent neuronal damage and cell loss that may occur in the brain of patients with mood disorders^29^,^30^,^31^,^32^. Although the links between hippocampal neurogenesis and psychiatric disorders are far to be elucidated, a better understanding of the regulation of neurogenesis by antidepressants and how they influence distinct phases of progenitor cell development may yield insights into the physiological mechanisms that underlie antidepressant behavioral efficacy.

As stated before, Agomelatine, Launched in European Union in 2009, was reported to induce neurogenesis and cell proliferation in the ventral part of dentate gyrus of patients, and brought great expectation in the clinic treatment of major depression. However, it was soon reported to have considerable hepatotoxicity, which should be the major reason why it was discontinued development for the US market in October 2011^33^. A number of observations imply that it is urgently desirable to find new chemical entities (NCE) as potential antidepressant candidates with enhanced benefit-risk balance.

In the field of modern medicinal chemistry, scaffold hopping strategy, a lead optimization method, has been widely used to discover novel drug candidates that bind to the same receptor or possess similar pharmacological effects. A change in the central chemical template of the lead compound can also lead to a granted patent and even enhanced ADME/T properties. There are now a lot of computational approaches to scaffold hopping. For example, the popular Maestro modelling software provides us ligand-based, structure-based and isosteric matching core hopping methods. However, it is still challenging to get alternative structures with synthetic tractability and at the same time conserve essential pharmacophore features. Furthermore, complicated similarity descriptors are hard to manage by the experimental pharmaceutial chemists and of little use if the scaffold hopping campaign starts from a single active compound only.

Inspired by aforementioned reasons and as a part of the ongoing work in our research groups aimed at the search of novel antidepressants^6^,^34^ with neuroprotective mechanism, we started a scaffold hopping campaign of Agomelatine in *silico.* Combination of the scoring function of fitting values and expertise, 3, 4-dihydroisoquinoline skeleton was selected as novel scaffold for chemical synthesis and the structure-activity relationship on C-1 position of this scaffold was extensively explored.

As known, PC12, a cell lineage derived from a pheochromocytoma of rat adrenal medulla, has been widely used to investigate the mechanisms involved in neurotoxicity, neuroprotection and neurorestoration^35^,^36^. Glucocorticoids at high concentration lead to PC12 neuronal damage under depressive disorder, and this feature makes PC12 cells very useful as a model system for *in vitro* screening^37^. As such, in this paper, we used corticosterone-induced lesion of PC12 cells as a rapid *in vitro* screening model for preliminary assessment of neuroprotective activity of the synthesize novel 3,4-dihydroisoquinoline compounds.

Thus, we disclose our studies on the design, synthesis and biological evaluation of a novel series of 3, 4-dihydroisoquinoline compounds as antidepressants. In the *in vitro* screening, compound **6a-1** was identified to possess highly neuroprotective effect and low cytotoxicities. Further *in vivo* FST and OFT experiments implies that compound **6a-1** is a potential potent antidepressant. To make clear the underlying mechanism of **6a-1** for the observed neuroprotective on PC12 cells, hoechst 33258 staining was performed to check out the apoptosis of corticosterone-induced PC12 cells. On the other hand, real-time PCR and ELISA method were also employed to analyze the trophic factors such as brain-derived neurotrophic factor (BDNF), nerve growth factor (NGF), Vascular endothelial growth factor (VEGF), Insulin-like growth factor 1 (IGF-1) *in vitro* or *in vivo*. Furthermore, the potential of compound **6a-1** to increase neuron cells survival, proliferation and maturation in the rat hippocampus was also investigated after chronic treatment.

## Scaffold Hopping

**Figure 1:**
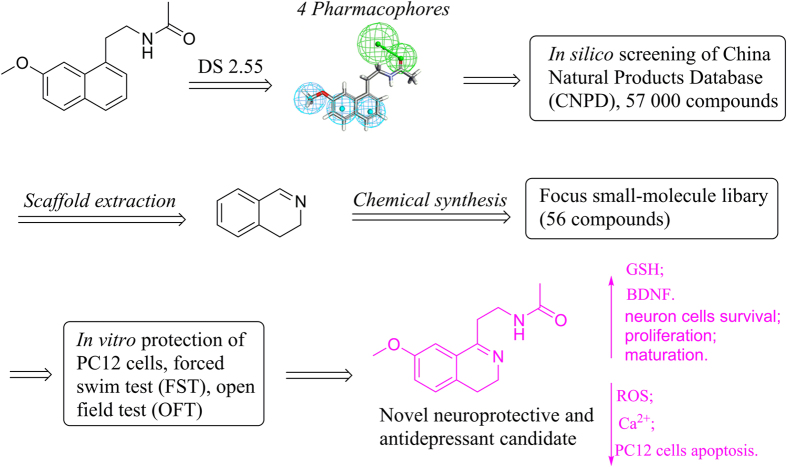
The flowchart of scaffold hopping and other study procedures.

As shown in Fig. 1, the 3D chemical structure of Agomelatine was generated by adding hydrogen and minimization with CHARMm field and Common Feature Pharmacophore protocol in Discovery studio 2.55 was employed to produce four pharmacophores (Figure S1), which were applied to screen Chinese Nature Products Database (CNPD) with Ligand Profiler protocol. 8254 compounds were successfully profiled and the top 500 hits with highest fitting values of each pharmacophore were combined and the duplicated hits were removed. 1061 compounds were remained for further analysis. Brain-blood barrier penetration is of first importance for antidepressants and favored by low molecular weight, lack of ionization at physiological pH, and lipophilicity. To meet the requirement of lack of ionization at physiological pH, 13 alkaloid compounds (as shown below) were visually inspected and selected from the 1061 molecules libarary. In order for facilely synthetic access and getting analogues with low molecular weight of nature products, the common structural moiety of the selected alkaloids, dihydroisoquinoline (this article) or tetrahydroisoquinoline (to be explored) core structure, can be extracted as novel scaffold for chemical synthesis and structure-activity relationship exploration.

## Chemistry

**Figure 2:**
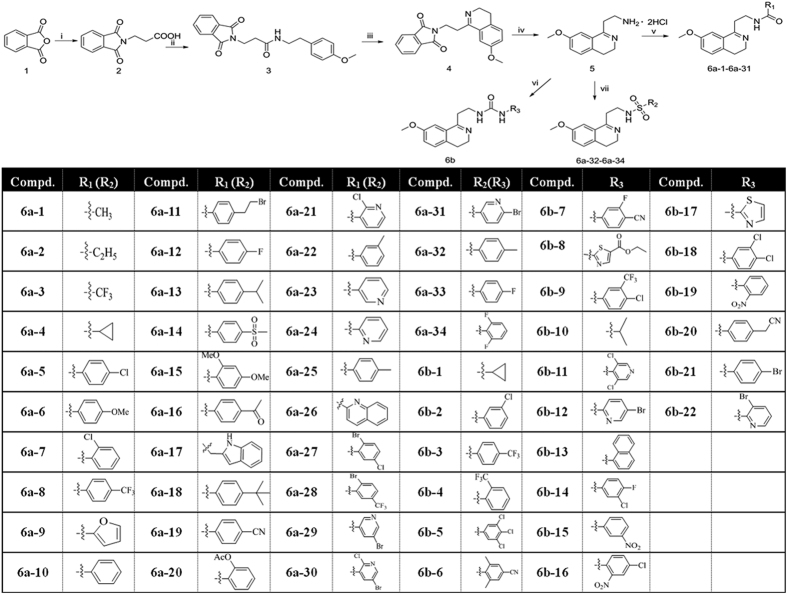
General synthetic route of 3, 4-dihydroisoquinoline compounds 6a and 6b. Reagents and conditions: (i) β-alanine, AcOH, reflux, 4 h; (ii) 2-(4-methoxyphenyl)ethan–1-amine hydrochloride, EDCI, Pyridine, r.t., 24 h; (iii) P_2_O_5_, POCl_3_, 130 °C, 4 h; (iv) HCl(aq.), reflux, 8 h; (v) Acyl chloride or anhydride/EDCI, r. t., 10 h; carboxylic acid/EDCI/pyridine, r.t., 10 h; (vi) amine, CDI, 0 °C-r.t., 10 h; (vii) sulfonyl chloride, EDCI, r.t., 10 h.

The target compounds **6a** and **6b** were obtained via multi-step synthesis according to Fig. 2. The first two steps are very straightforward. The *N*-protected β-alanine **2** was readily prepared by refluxing the mixture of isobenzofuran-1,3-dione **1,** β-alanine and acetic acid for 4 hours according to literature^38^,^39^ with appropriate revision. Then intermediate **2** was condensed with 2-(4-methoxyphenyl)ethanamine hydrochloride at room temperature to give intermediate **3** in high yield^40^.

According to a reported method, intermediate **4** can be conveniently obtained by strategy of Bischler-Napieralski reaction^41^. However, the usual reaction and workup conditions for Bischler-Napieralski reaction did not work in our case. The key issue is of incomplete conversion and mixed unknown impurities. After several attempts, we finally found that heating the intermediate **3** with 6.0 eq. POCl_3_ and 3.0 eq. P_2_O_5_ at 130 °C for 4 hours could reach a complete and neat conversion to the target compound. The reaction mixture was cooled down to room temperature naturally, and the excessive POCl_3_ was decanted carefully. Then the semisolid residue was gradually buffered to pH 10-11 by diluted NaOH solution with stirring at 60–70 °C. The aqueous solution was extracted with ethyl acetate/dichloromethane (4/1, v/v). To note, the working efficiency is extremely low if this workup was performed at room temperature because the asphalt-like residue was hard to agitate, although generally low temperature was believed to be beneficial for the product stability.

Accordingly, compound **5** was obtained by acid hydrolysis of the key intermediate **4** in a yield of 86.7%. The final compounds **6a** were easily made by acylation of compound **5** with the corresponding acyl chloride, or acid anhydride, or carboxylic acid, or sulfonyl chloride at appropriate conditions. Meanwhile, compounds **6b** were afforded by condensation compound **5** with corresponding amine with carbonyl diimidazole (CDI) under mild condition^42^. Through above procedures, 56 novel 3, 4-dihydroisoquinoline compounds were synthesized and isolated in moderate to high yields.

## Result and Discussion

### Protective effects on corticosterone-injured PC12 cells

As reported before^37^, corticosterone-injured PC12 cells is a fruitful *in vitro* model for preliminary screening of antidepressant drugs. All the target compounds were tested for their protective activity on this *in vitro* model at drug concentration of 1.25, 2.5 and 5 μM. The data were shown in Table 1 (and Supplementary material Table S1). Ten of them (**6a-1**, **6a-2**, **6a-9**, **6a-21**, **6a-27**, **6b-3**, **6b-4**, **6b-12**, **6b-17** and **6b-18**) demonstrated potential protective effects (above 10%) on corticosterone-induced lesion of PC12 cells at concentration of 1.25 μM. Three amide compounds (**6a-1**, **6a-2** and **6a-9**) showed pronounced efficacy with protection rates of 25.4%, 32.7% and 20.3% respectively and compound **6a-1** showed a good concentration-dependant manner. As for structure-activity relationship, two prominently active compound **6a-1** and **6a-2** are substituted with methyl and ethyl group at the same position, which suggests that steric hindrance of the side chain may have an influence on the activity. Last, another active compound **6a-9** was introduced with a furan ring into its side chain, where the electron-rich furan ring may contribute to decrease the ROS level in the stimulated PC12 cells.

### Cytotoxicities

In order for assessment of their potential cytotoxicities, the inhibitory effects of the final 3, 4-dihydroisoquinoline compounds were assessed *in vitro* on L02 cells and HEK293 cells. As shown in present data(see Supplementary material Table S2), most of the target compounds displayed low toxicities on the tested cells. Four compounds (**6a-1**, **6a-16**, **6b-6** and **6b-13**) showed inhibitory rates lower than 20% on both cells at concentration of 100 μM. The neuroprotection active compound **6a-9** exerted higher inhibition on growth of HEK293 and L02 cells. Other compounds displayed comparable effects to the positive control, Agomelatine. The most promising compounds **6a-1** exerted higher safety profile on HEK293 (10.3%) and L02 (13.7%) cells, superior to Agomelatine (47.5% and 41.8%).

### BBB penetration ability calculation and assay

Lipophilicity is generally regarded as a most important physicochemical property largely related to ultimate success in drug discovery and development. It was also identified as an important determinant of central nervous system (CNS) exposure, including both the rate and extent of drug distribution into the brain. Accordingly, a CLogP value of around 2.0 were suggested to be the most optimal for CNS drugs. Described as a surrogate measure of hydrogen-bonding capacity and molecular polarity, the tPSA is a commonly used metric during the optimization of a drug’s ability to permeate cell membranes^43^. Therefore, CLogP and tPSA were calculated for early assessment of their CNS drug-likeness. The values of ClogP and tPSA for the synthesized compounds were calculated. Accordingly, we find out that the CLogP values of all target compounds fall into the CLogP range (0.16–6.59) of the marketed CNS drugs. As for tPSA, three compounds (**6b-15**, **6b-16** and **6b-19**) go beyond the tPSA range (4.63–108) of the marketed CNS drugs. It suggested that the nitro group in these three compounds is not favored by BBB penetration(see Supplementary material Table S3).

Further, a PAMPA-BBB experiment was performed to measure the CNS permeation ability according to a report method with minor revision^44^. The results were listed in Table 2. Pe values of eleven drugs were also assayed for validation the method. The Pe value of compound **6a-1** is very close to that of Agomelatine, which make us believe compound **6a-1** may have antidepressant effects *in vivo*.

### Forced swim test

**Figure 3:**
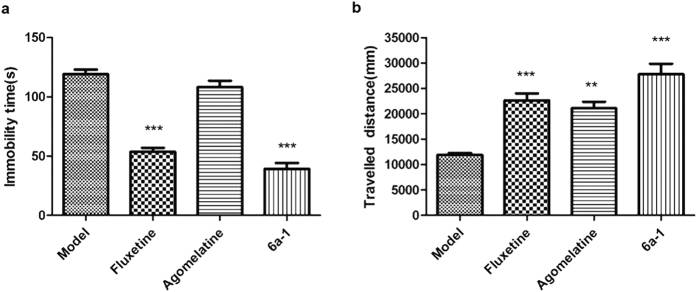
*In vivo* antidepressive effects evaluation. Forced swim test and Open field locomotor activity test. (**a**) Effects of compound **6a-1** and positive controls in forced swim test; (**b**) Antidepressant-like effects of compound 6a-1 and positive controls in the open field test. SD rats were treated at the same time of each day of days 2–15 (ig.32 mg/kg/day). Data represent the mean ± S.D. of 10 rats per group. Values are significant at *P < 0.05 when compared with model group treated with vehicle.

The forced swim test has been widely used as a predictive model of depressive behavior in pre-clinical test^45^. The FST data of SD rats were shown in Fig. 3a. In the group treated with compound **6a-1,** the mean immobility time of the swimming SD rats was reduced by 62.5% compared to the model group. The same parameter for Fluoxetine is 50.7%, for Agomelatine is 8.4%. In our animal models, Agomelatine did not significantly reduce the immobility time of the swimming SD rats. Compound **6a-1** displayed pronounced antidepressant-like activity compared to the model group treated with vehicle. In this test, its effects is similar to that of Fluoxetine.

### Open field locomotor activity

The open field test (OFT) evaluates the general locomotor and exploratory behavior of rats. Sometimes the nonspecific motor activities may lead to false-positive results in the forced swim test. Hence, we employed open field apparatus to further test its spontaneous locomotor activity and the data was shown in Fig. 3b. After treated with compound **6a-1**, SD rats travelled longer distance than the ones did not treated drugs, which improved the travelled distance by 131.2%. When rats were treated with Fluoxetine or Agomelatine, the percentage of improved travelled distance was 69.8% and 91.2%, respectively. The above data implied that compound **6a-1** can treat the depressant behavior in SD rats model.

### Measurement of intracellular GSH and ROS level

**Figure 4:**
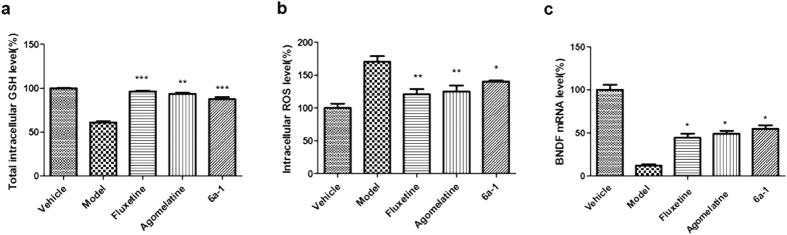
Mechanism studies *in vitro* of compound 6a-1. Effect of compound **6a-1** regulate the total GSH level or intracellular ROS level or BDNF mRNA levels of corticosterone injured PC12 cells. PC12 cells were treated with normal saline (control); 200 μM of corticosterone (vehicle); 200 μM of corticosterone and 5 μM of drugs, respectively; Values are expressed as mean ± S.D. (n = 6) *P < 0.05.

Accumulation of oxidative stress has been noted in the brain of stress-induced animal models and oxidative stress is considered to be a mechanism of major depression^46^. High corticosterone level positively correlates with the oxidative stress in stress-induced animal models and stress-triggered depression patients. It helps antidepressant mechanism elucidation to investigate the anti-oxidative effects of our compound. The GSH level and ROS level of vehicle-treated PC12 cells were designated as 100%. In corticosterone injured PC12 cells, the GSH level was reduced to 60.9%, compared with vehicle control (Fig. 4a). However, compound **6a-1** can antagonize the GSH down-regulation induced by corticosterone effectively, where the GSH level was 87.7%. The data for Fluoxetine and Agomelatine were 96.3% and 93.4%, respectively. On the other hand, the ROS level in corticosterone injured PC12 cells was increased to 170.4% (Fig. 4b). Compound **6a-1** can antagonize the ROS up-regulation induced by corticosterone effectively, where the ROS level was 140.3%. The ROS level of Fluoxetine and Agomelatine were 120.7% and 125.0%. The remarkable decrease of ROS level and up-regulation of GSH level induced by compound **6a-1** may alleviate the oxidative stress in nerve cells, which may improve the treatment of depressed subjects.

### Detection of mRNA level of BDNF and NGF

To examine whether brain-derived neurotrophic factor Trophic factor (BDNF) and nerve growth factor (NGF) were implicated in the neuoprotective effects of compound **6a-1** on corticosterone injured PC12 cells. The BDNF mRNA level of corticosterone injured PC12 cells decreased by 87.8% compared with control (Fig. 4c). Compound **6a-1** was shown to up-regulate the BDNF level by 42.8% compared with corticosterone-injured group, while the Agomelatine and fluoxetine only increased mRNA levels of BDNF by 37.2% and 32.1% compared with corticosterone-injured group. These data suggested BDNF play a part in protective effect of compound **6a-1** on corticosterone-injured PC12 cells. On the other hand, the NGF mRNA levels almost remained untouched among different groups (See see Supplementary material Figure S4).

### Hoechst 33258 staining

It is well established that endogenous oxidative stress can induce neuron cell apoptosis^47^. To detect the antagonism effect of compound **6a-1** on corticosterone-induced PC12 cells apoptosis, we took advantage of Hoechst 33258 staining (see Supplementary material Figure S5). Cells exhibiting abnormal nuclei (crenation, condensation, and fractionation) were regarded as apoptotic cells. It is no difficulty finding out that apoptosis of corticosterone injured PC12 cells were the most severe. Compound **6a-1**effectively reversed apoptosis of PC12 cells induced by corticosterone.

### Intracellular calcium ion concentration analysis

Although the calcium intake is helpful for the depression patients, intracellular Ca^2+^ overload can trigger either necrotic or apoptotic cell death. And prevention of intracellular Ca^2+^ overload will be of importance for neuroprotection^48^. Thus we employed Thermo Scientific Array Scan Infinity to test fluorescence intensity of free intracellular calcium ion (see Supplementary material Figure S6). As the results showed, in corticosterone-injured PC12 cells, free calcium ion concentration was obviously overloaded compared with normal control. Fluoxetine and Agomelatine exhibited weaker fluorescence intensity than corticosterone-injured PC12 cells, suggested their suppression on calcium ion overload. To our delight, compound **6a-1** demonstrated much weaker fluorescence intensity than Fluoxetine and Agomelatine, implied compound **6a-1** may possess better neuroprotective activity.

### Cell survival, proliferation and maturation in rat hippocampus

**Figure 5:**
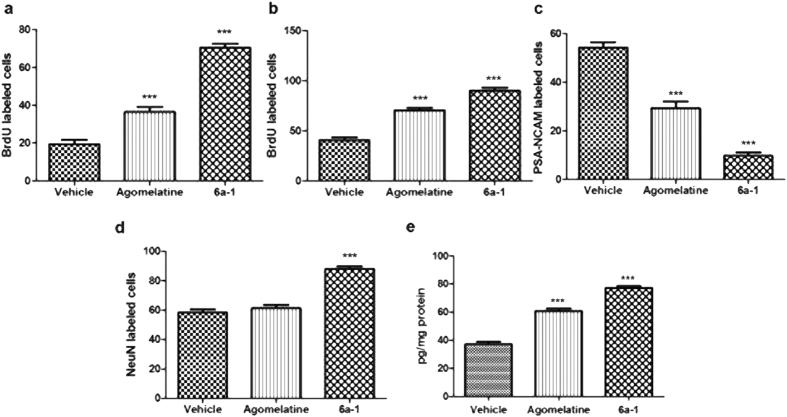
Mechanism studies *in vivo* of compound 6a-1. Analysis of survival, cell proliferation, maturation and BNDF level in the rat hippocampus. (**a**) Compound **6a-1** increases cell survival *in vivo*; (**b**) Compound **6a-1** increases cell proliferation *in vivo*; (**c,d**) Compound **6a-1** increases cell maturation *in vitro*. Results are means ± SD of the number of BrdU-labeled cells for n = 6rats per group. (**e**) Compound **6a-1** increases BDNF level*in vivo*. Results are means ± SD pg/mg total protein for six rats per group.

It has been clarified that cell proliferation and neurogenesis are generally reduced in animal models of depression and increased by chronic antidepressant treatments^49^. Bromodeoxyuridine (5-bromo-2′-deoxyuridine, BrdU) is a synthetic nucleoside that is an analog of thymidine and commonly used in the detection of cell survival and proliferation in living tissues. To evaluated effects of compound **6a-1** on cell survival and proliferation after 21 days treatment, BrdU was injected either at beginning (survival) or the end (proliferation) of drug treatments. Rats were treated once daily with compound **6a-1** or Agomelatine (i.p. 40 mg/kg). Hippocampal BrdU-labeled cells were quantified and the results showed the number of BrdU-labeled neuron cells were remarkably increased after compound **6a-1** treatment in both survival (Fig. 5a, Supplementary material Figure S7c) and proliferation (Fig. 5b, Supplementary material Figure S7d) groups, much significantly than that of Agomelatine. The data indicated that compound **6a-1** increases neuron cell survival and proliferation *in vivo*.

On the other hand, the degree of maturation of newly formed cells labeled with BrdU *in vivo* was determined at 21 days of development by a combination of polysialic acid form of neural cell adhesion molecule (PSA-NCAM) and a neuronal nuclear antigen (NeuN) labeling. Treatments with either compound **6a-1** or Agomelatine for 21 days reduced the expression of PSA-NCAM compared with vehicle-treated group. The compound **6a-1** group showed an approximately three-fold decrease in the number of PSA-NCAM-labeled cells than that of Agomelatine group (Fig. 5c, Supplementary material Figure S7a). In addition, compared with vehicle-treated group, only compound **6a-1** induced a significant (P < 0.001) increase in the number of NeuN cells (Fig. 5d, Supplementary material Figure S7b). The results showed a highly significant increase in number of mature neurons after chronic treatment with compound **6a-1**.

### Detection of BDNF, VEGF and IGF-1 Level *In Vivo*

As accumulating evidence supports that antidepressants stimulate growth factors such as BDNF, IGF-1 and/or VEGF expression generally enhance adult neurogenesis and may exert behavioral antidepressant-like effects^50^,^51^. Levels of VEGF, IGF-1 and BDNF protein were measured in the hippocampus after 21-day treatment (i.p. 40 mg/kg). Hippocampal extracts were analyzed by ELISA assays. Compared with Agomelatine treatment, compound **6a-1** induced similar increase in hippocampal BDNF level (Fig. 5e), while neither compound **6a-1** nor Agomelatine exerted any influence on the level of VEGF, IGF-1 when compared with vehicle-treated group (see Supplementary material Figure S8).

### *In vivo* toxicity evaluation

**Figure 6:**
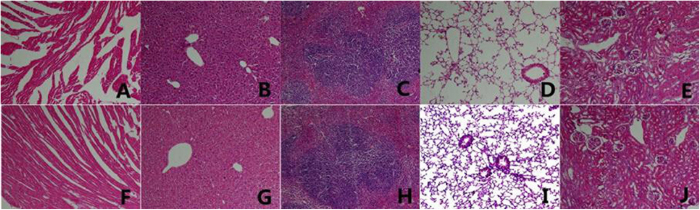
Histomorphological examination of main organs of C57 mice after one week compound 6a-1 treatment. (**A–E**) was the Heart, Liver, Spleen, Lung, Kidney of compound **6a-1** treated mice, (**F–J**) was the Heart, Liver, Spleen, Lung, Kidney of Agomelatine treated mice.

Compound **6a-1** was administered to adult C57 mice in an intragastric manner at dose of 140 mg/kg/day. No visible clinical signs of toxicity, such as irritability, twisting, righting reflex, tremors, convulsions, breathing, weight loss, or death, were observed during 7-day administration. Ten mice of each group were sacrificed on the 7th day, their heart, liver, spleen, lung and kidney were harvested and H&E histological staining was performed. Figure 6A,F represents an optical micrograph of heart tissue of mice treated with compound **6a-1** and Agomelatine, respectively. Cardiac myocytes were clear and arranged in good order, with no necrosis, hemorrhage or inflammatory exudates were observed in Agomelatine group. As for compound **6a-1** group, cardiac myocytes were fragmented and arranged promiscuously after treated with compound **6a-1**. The hepatic cords were distinctly clear in the group of compound **6a-1**(Fig. 6B), while in Agomelatine group (Fig. 6G) they were severely damaged. As shown in Fig. 6C,H, the tissue structure of the spleen in two groups were unchanged, spleen sinus did not show any pathological changes. The lung and kidney treated with compound **6a-1** (Fig. 6D,E) also did not show any significant difference compared with normal. The above results showed that liver toxicity, the major concern of Agomelatine, was not observed in compound **6a-1**.

### Inhibitory effects of compound 6a-1 on H9C2 cells growth and hERG K^+^ channel

Although compound **6a-1** significantly decreased the hepatotoxicity, the unexpected cardiotoxicity raised a critical issue and drew our concerns. It is desirable to clarify the underlying mechanism for further structural optimization. Then we firstly evaluated the *in vitro* cytotoxicity of compound **6a-1** on H9C2 cells, a permanent cell line derived from rat cardiac tissue. The IC_50_ value of compound **6a-1** was 455.0 μM, higher than Agomelatine (374.7 μM, Table S4). This result implicated the compound **6a-1** has little effects on H9C2 cells growth.

As we know, the *in vitro* hERG potassium channel activity in mammalian cell lines can be tested to predict QT prolongation risk, which is frequently associated with potentially lethal arrhythmias^42^. Thus the effects of compound **6a-1** on hERG channel was further investigated to clarify its QT prolongation risk. As a result, we tested the hERG K^+^ channel inhibition of compound **6a-1** (Table S4) and the IC_50_ value was over 40 μM, indicated that compound **6a-1** has minor effects on hERG K^+^ channel.

From above *in vitro* studies, a reasonable interpretation of the observed *in vivo* cardiotoxicity could not be reached and more studies are still needed. Interestingly, Neferine, one of the four natural products we used to extract the chemical scaffold in this paper, was reported to possess potential cardiotoxicity by disruption of calcium homeostasis^52^. In our case, compound **6a-1** downregulated the calcium overload in corticorsterone-injured PC12 cells. It will help to decipher the intrinsic relationship between these two observations and suggest a probable direction in future studies.

### Test on FLIPR assays

Given that 5-HT_2C_ receptor was involved in the effect of Agomelatine on cell proliferation, maturation and survival, we tested compound **6a-1** on 5-HT_2C_ antagonism effects with FLIPR assays. As shown in Table S5, the IC_50_ value of compound **6a-1** was higher than 100 μM, indicated the major biological target of compound **6a-1** is different from Agomelatine. This observation tells us that there may be a possibility that novel bioactive compounds discovered from scaffold hopping strategy may take biological effects with changed biological target(s).

## Conclusions

Based on virtual screening of CNPD library with pharmacophores generated from the marketed drug Agomelatine, 3, 4-dihydroisoquinoline scaffold was selected as new scaffold of novel Agomelatine analogues for further chemical synthesis and structure-activity relationship investigation. Accordingly, fifty-six novel 3, 4-dihydroisoquinoline compounds were synthesized by altering the C-1 substituted groups of the skeleton. The compounds **6a-1** and **6a-2** with small steric hindrance were found to possess highly neuroprotective effects on corticosterone-injured PC12 cells. Further results from *in vitro* cytotoxicities tests of compound **6a-1** on HEK293 and L02 cells, and BBB permeation ability make it worthy of *in vivo* animal studies. The FST and OFT of SD rats implied compound **6a-1** possesses obvious antidepressant effects on animal models. Mechanism studies implicated that compound **6a-1** can significantly reduce PC12 cell apoptosis by up-regulation of GSH and down-regulation of ROS in corticosterone-induced lesion of PC12 cells. Meanwhile, the down-regulation of calcium ion concentration and up-regulation of BDNF level in PC12 cells may account for the neuroprotective effects. Analysis of cell survival, cell proliferation and maturation in the rat hippocampus show compound **6a-1** can increase cell survival and cell proliferation, promote cell maturation after a chronic treatment. Besides, the acute toxicity data *in vivo* indicated compound **6a-1** exhibited less hepatotoxicity than Agomelatine. Although more studies are needed to elucidate the exact action molecular target(s) and mechanism of the observed cardiotoxicity *in vivo,* compound **6a-1** provides us insights for further structural optimization and discovery of novel antidepressant agents with neuroplasticity mechanism. Our study also demonstrated a successful scaffold hopping approach in the process of drug lead discovery by a combination of *in silico* screening and knowledge-based analysis.

## Experiment Section

### Corticosterone-induced PC12 cells lesion and protective effects of the 3, 4-dihydroisoquinoline compounds

PC12 cells were purchased from American Type Culture Collection and were maintained in DMEM medium supplemented with penicillin (100 unit/ml), streptomycin (100 μg/ml), 5% fetal bovine serum (FBS), and 10% horse serum at 37 °C in humidified atmosphere with 5% CO_2_. The detailed procedures were performed referring to the reported literatures^6^. Briefly, PC12 cells of logarithmic growth phase were collected, re-suspended and then seeded at a density of 1 × 10^5^ cells per well in 96-well plates and cultured in the DMEM medium with 5% horse serum, 10% FBSfor 24 h.

After that, the upper medium in the 96-well plates was absorbed and then the PC12 cells were treated with 100 μl of 200 μM corticosterone for 1 h and then respectively co-incubated with Fluoxetine, Agomelatine or other compounds for another 24 hour.

After incubation with compounds, 20 μl of 5 mg/ml MTT solution was added to each well and cultured at 37 °C for 2 h–4 h. Then, the culture medium was removed, and 150 μl dimethyl sulfoxide(DMSO) was added to each well for 15 min at room temperature. The absorbance of each well was measured at 570 nm using a microplate reader. The protective rates (PR) were calculated according to equation (1).


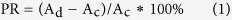


### Cytotoxicity evaluation

Human normal hepatic L02 cells, human embryonic kidney cell line 293 cells (HEK293) and Rattus myoblast cell line H9C2 cells were respectively seeded in 96-well plates at a density of 4 × 10^3^ cells per well and cultured in the 1640 or DMEM medium, with the supplement of 10% fetal bovine serum, penicillin (100 unit/ml), streptomycin (100 μg/ml) in a humidified incubator with 5% CO_2_ for 24 h. After adherence, L02, HEK293 and H9C2 cells were exposed to different compounds for another 48 h. The cytotoxicities of the compounds were evaluated by MTT method.

### BBB permeation ability measurement

Parallel artificial membrane permeation assay (PAMPA) was performed according to the literature^44^ with minor modification. The PVDF (Poly(vinylidene fluoride)) membrane of the donor plate was coated with PBL (Polar Brain Lipid, Avanti, USA) in dodecane (4 μL of 20 mg/mL PBL in dodecane) and the acceptor well was filled with 350 μL of PBS/EtOH (70/30, v/v, pH 7.4) buffer (V_D_). The tested compound was dissolved in DMSO and then diluted with PBS/EtOH (70/30, v/v, pH 7.4) buffer to reach the concentration of 100 μg/mL in the donor well.

The concentration of DMSO did not exceed 0.5% (v/v) in the donor solution. 200 μL of the donor solution was added to the donor wells (V_A_) and the donor filter plate was carefully put on the acceptor plate so that the coated membrane contacted both donor solution and acceptor buffer. Test compound diffused from the donor well through the lipid membrane (area = 0.28 cm^2^) to the acceptor well. The concentration of the drug in both donor and the acceptor wells was assessed after 18 hours of incubation at room temperature in triplicate using Varioskan Flash Multimode Reader (Thermo Scientific) at the maximum absorption wavelength of tested compound. Concentration of the compound was calculated from the standard curve and expressed as the permeability (Pe) according to Equation (2):


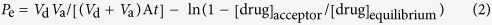


### Animals

All animal methods in this study were carried out in accordance with guidelines and regulations of the Ethical Committee of Sichuan University for the use of Laboratory Animals and approved by the Institutional Animal Care and Treatment Committee of Sichuan University and all animals in this study were treated humanely throughout the experimental period. Sprague Dawley (SD) rats weighing 200–230 g were placed individually in cages maintained at 25 °C and were fasted overnight but free access to water before experiments. 6~8 week-old male Wistar rats were group-housed under standard conditions (12-h light/dark cycle, 22 ± 2 °C, food and water ad libitum). BALB/c mice (8 to 12 weeks old; 20–30 g) used in this study were kept in temperature controlled (24 ± 1 °C) rooms with food and water given ad libitum. The detailed procedures were described in the following different experiments.

### Forced swim test

The detailed procedures were performed according to our previous studies^6^,^34^. Briefly, each SD rat was placed in a vertical Plexiglas cylinder (height 50 cm; diameter 20 cm) containing 18 cm height of water at 25 ± 2 °C and was forced to swim individually for 15 min on1^st^ day. From 2^nd^ day to 15^th^ day, the rats were intragastrically administered compound **6a-1** or control drugs at dosage of 32 mg/kg/day. On the 2^nd^ day and 15^th^ day, 30 minutes after drug treatment, each rat was placed again into water and forced to swim for 6 min. The rat behavior was recorded by a video camera placed directly facing cylinders. The duration of immobility during the last 4 minutes was analyzed by Xeye Animal behavior analysis system (Xeye Aba V3.2). The rat was considered as immobile when its moving speed was less than 20 mm/s. The dosage of is 32 mg/kg every day. The water in the cylinder was changed each trial. After each exposure, animals were partially dried with a towel and returned to their home cages. All tests were performed in a quiet room.

### Open field locomotor activity

The detailed procedures were performed according to our previous studies^6^,^34^. In brief, each SD rat was placed at the center of the open field (50 × 50 cm^2^ chamber, 50-cm-high walls, with a 25 cm^2^ area in the middle of floor defined as the central square) for 6 min in a quiet room. The animals were gently placed in the center of the platform and were allowed to explore the surroundings. A camera was installed above the center of the field. Immediately after a rat was placed at the center in the open field, the movements and position of the animals were recorded and registered automatically by computerized system. Next test was performed after cleaning the chamber. The traveled tracks of rats were recorded for 6 minutes and the videos of the last 4 minutes were analyzed also by Xeye Animal behavior analysis system.

### Measurement of intracellular ROS and GSH level

The detailed procedures for ROS level detection were performed according to the reported literatures^53^,^54^. In brief, PC12 cells were washed with D-Hanks after drug treatment. Then cells were incubated with 2′, 7′-dichloro-fluorescein diacetate (DCF-DA, 20 μM) for 30 min at 37 °C in darkness. The ROS induced fluorescence intensity was measured by microplate reader at an excitation wavelength of 485 nm and an emission wavelength of 538 nm.

The GSH level was measured by reference of literatures’ methods^55^,^56^ with appropriate revision. Briefly, A total number of 1 × 10^6^ cells treated with compound **6a-1** or positive controls were collected and centrifuged at 2000 rpm for 5 min and the cell pellets were lysed using ultrasonic irradiation in 200 μL of ice-cold RIPA lysis buffer with protease inhibitor cocktail. After being incubated on ice for 10 min, the lysate was centrifuged at 10 000 rpm for 10 min. 100 μL of the supernatant was mixed with 200 μL of trichloroacetic acid (25%) and 200 μL of saline. The mixture was centrifuged at 3000 × g for 10 min at 4 °C, and then 200 μL of the resulting supernatant was mixed with 1 mL of phosphate buffer (100 mM, pH 8.0) and 50 μL of 5, 5-dithiobis-2-nitrobenzoic acid (DTNB). The solution was maintained at room temperature for 5 min and its absorbance measured at 412 nm.

### Real-time PCR detection mRNA level of BDNF and NGF

The detailed procedures were performed according to the reported literatures^57^,^58^. Briefly, total RNA from hippocampus of drug treated C57 mice was isolated using TRIzol^®^ reagent (Invitrogen, USA) according to the manufacturer’s instructions. Quantification of mRNAs was performed on Bio-rad CFX96TM Real-Time PCR Detection System (Bio-rad, USA). The sequences of gene-specific primers were as follows: BDNF forward primer, CCCATCACAATCTCACGGTA, BDNF reverse primer, ACAGGACGGAAACAGAACGA; NGF forward primer, CCTTCAACAG -GACTCACAGGA, NGF reverse primer, TCTCCAACCCACACACTGAC.

### Hoechst 33258 staining

To detect apoptotic cells, drug treated cells were stained with the DNA dye Hoechst 33258. Cells with the indicated treatment were fixed with methanol for 10 min at 4 °C before incubation with Hoechst 33258 for 10 min at room temperature. After washes with PBS, the apoptotic cells were mounted onto slides and observed under the fluorescence microscope BX61 (Olympus, *J*apan). Images were captured using DP71 CCD digital camera (Olympus).

### Intracellular Calcium ion concentration analysis

The detailed procedures were performed according to the reported literatures^59^. In brief, calcium ion concentration was measured by Fura-2 AM (Thermo Scientific). PC12 cells were treated with compound **6a-1** for 48 hours. Then cells were detached with 0.25% trypsin and centrifuged at 1000 r/min for 5 minutes. The supernatant was removed and the cells were stained with Fura-2 AM according to the manufacturer’s instructions. Fluorescence intensity of labeled cells was photographed for 25 random sights each group using Thermo Scientific Array Scan Infinity.

### Analysis of cell survival, proliferation and maturation in the rat hippocampus

The detailed procedures were performed according to the reported literatures^49^.

Wistar rats were used herein. Agomelatine were purchased from Dalian Meilun Biotech Company. Compound **6a-1** (40 mg/kg) or Agomelatine (40 mg/kg) was injected as a suspension in 1% Hydroxyethyl cellulose at 40 mg/kg i.p. once a day for 21 days. For cell proliferation study, BrdU (200 mg/kg i.p.) was administered 2 h before perfusion. For cell maturation and survival studies, animals received five injections of BrdU (75 mg/kg, 2 h intervals) the first day of treatment and were killed 21 days later. Each mouse was randomly selected 8 visual fields for statistical analysis.

### BDNF levels measurement *in vivo*

The detailed procedures were performed according to the reported literatures^49^.

SD rats were killed by decapitation and hippocampi were dissected out and stored at −80 °C until use. Tissue samples were homogenized at 4 °C in Promega lysis buffer for ELISA. Samples were then sonicated and lysates were cleared by centrifugation at 4 °C, 15 000 g. Protein concentrations were determined according to the Bradford assay using bovine serum albumin as standard. Quantifications of VEGF and IGF-1 proteins were measured with Quantikine M mouse VEGF and IGF-1enzyme immunoassay kits and BDNF protein with a mouse ELISA kit.

### Acute toxicity evaluation

In brief, all C57 mice were carefully observed after administration of compound **6a-1** or Agomelatine at doses of 140 mg/Kg/day for their general conditions. Ten mice of each group were sacrificed on the 7th day, their heart, liver, spleen, lung and kidney were harvested and H&E histological staining was performed. Then the stained tissues were observed by two pathologists in a blinded manner.

### Inhibition evaluation on hERG K^+^ channel

The detailed procedures were performed according to the reported literatures^60^. In brief, A Chinese hamster ovary (CHO) cell line stably expressing hERG potassium channels were cultured into appropriate cell density. Then cells were automatically prepared for application to chips. Whole-cell recordings were performed using automated QPatch (Sophion) and the data were analyzed using Assay Software provided by Graphpad Prism 5.0.

### FLIPR assays for 5-HT_2c_ antagonism

Seed cells at the density of 10 K cell/well, 37 °C, 5% CO_2_ incubate for 16–24 hours, then loading cells with 30 ul of Calcium 5 and 37 °C, 5% CO_2_ incubate for 1 hour. After transfer compound to compound plate with 30 ul Assay Buffer by Echo, add 15 ul/well of compound, incubate for 10 mins. Then 22.5 ul/well of inducer were added and measure calcium flux signal with FLIPR.

### Statistical analysis

Data are showed as mean ± S.D. Statistical analyses were performed with one-way ANOVA followed by Dunnett’s test for multiple comparison procedures. The statistics were performed by GraphPad Prism 5.0 software. The level of statistical significance was set at P < 0.05. (*P < 0.05, **P < 0.01,***P < 0.001).

#### Chemistry

All solvents and reagents were analytical reagents and used directly without further purification. All melting points were determined on a SGW X-4 MicroMelting Point apparatus without corrected. ^1^H NMR and ^13^C NMR spectra were recorded on a Bruker Avance (Varian Unity Inova) 400 MHz spectrometer, with TMS took as internal reference chemical shift in *δ*, ppm. High-resolution mass (HRMS) spectrometry was carried out on a Waters Q-TOF Premier mass spectrometer. All compounds used for biological assays were at least of 98% purity based on HPLC analytical results monitored with full wavelengths.

#### 3-(1,3-Dioxoisoindolin-2-yl) propanoic acid (2)

To a flask was added 1, 3-isobenzofurandione(300.0 g, 2.03 mol), β-alanine (180.0 g, 2.03 mol) and acetic acid (2 L) and then refluxed at 120 °C for 4 h. After the completion of reaction, the solvent was evaporated under reduced pressure and the solid residue was washed with water to pH 7 and dried to afford the title compound(421.2 g, yield: 95%), mp: 150–151 °C. The NMR and MS spectral data were in accordance with the literature.

#### 3-(1,3-Dioxoisoindolin-2-yl)-N-(4-methoxyphenethyl) propanamide(3)

Compound **2** (384.0 g,1.75 mol), 2-(4-methoxyphenyl) ethanamine hydrochloride(394.1 g, 2.10 mol) and EDCI (671.7 g, 3.50 mol) were dissolved in 4 L pyridine. After stirring at room temperature for 4 h, the resulting mixture was concentrated under reduced pressure, then buffered to pH 2 with hydrochloric acid and extracted with DCM (500 mL × 3). The combined organic layer were concentrated under reduced pressure to obtained the crude product, which was recrystallized with ethyl acetate. The title compound was obtained as pale white powder (483.6 g, yield: 78.3%). mp: 150–151 °C. ^1^H-NMR (CDCl_3_): *δ* 7.84 (dd, *J*_1_ = 5.6 Hz, *J*_2_ = 3.2 Hz, 2H), 7.19 (dd, *J*_1_ = 5.2 Hz, *J*_2_ = 2.8 Hz, 2H), 7.08 (d, *J* = 4.2 Hz, 2H), 6.81 (d, *J* = 8.4 Hz, 2H), 5.06 (br, 1H), 3.99 (t, *J* = 7.2 Hz, 2H), 3.78 (s, 3H), 3.48 (dd, *J*_1_ = 12.8 Hz, *J*_2_ = 6.8 Hz, 2H), 2.73 (t, *J* = 7.2 Hz, 2H), 2.57 (t, *J* = 7.2 Hz, 2H); MS (ITMS) *m/z* calcd. for C20H20N2O4 [M+H^+^]: 352.14, found: 352.14.

#### 2-(2-(7-Methoxy-3,4-dihydroisoquinolin-1-yl) ethyl) isoindoline-1,3-dione(4)

Compound 3 (70 g, 0.20 mol) were dissolved in xylene (400 mL), and then P_2_O_5_ (36 g, 0.25 mol) and POCl_3_ (110 mL) were added in order. The mixture was heated to 125 °C and string. After 4 h, the xylene was removed and the mixture was buffered to pH 10 with hydrochloric acid, extracted with ethyl acetate and DCM (EA/DCM: 3/1, 800 mL × 5). The organic layer was combined and evaporated and the crude products was separated by flash column (elute DCM/MeOH: 100/1) to obtained the title compound as yellow solid (33.5 g, yield 50.8%), mp: 161–163 °C; ^1^H-NMR (DMSO-*d*_*6*_): *δ* 7.81 (dd, *J*_1_ = 5.6 Hz, *J*_2_ = 3.2 Hz, 2H), 7.69 (dd, *J*_1_ = 5.6 Hz, *J*_2_ = 2.8 Hz, 2H), 7.09–7.06 (m, 2H), 6.84 (dd, *J*_1_ = 8.4 Hz, *J*_2_ = 2.4 Hz, 1H), 4.07 (dd, *J* = 7.6 Hz, 2H), 3.81 (s, 3H), 3.64 (t, *J* = 7.6 Hz, 2H), 3.08 (t, *J* = 7.2 Hz, 2H), 2.61 (t, *J* = 7.2 Hz, 2H); 13C NMR (DMSO-*d*_*6*_): *δ* 167.72, 163.61, 158.12, 134.27, 131.61, 129.13, 128.97, 128.29, 122.87, 116.11, 110.08, 55.17, 46.57, 35.44, 33.28, 24.25; HRMS (TOF) m/z calcd. for C20H18N2O3 [M+H^+^] 335.1396, found: 335.1339.

#### 2-(7-Methoxy-3,4-dihydroisoquinolin-1-yl) ethanamine hydrochloric acid (5)

To a flask with compound **4** (33.5 g, 0.1 mol) was added concentrated hydrochloric acid (500 mL) and heated to reflux for 8 h. After the completion of reaction, the solvent was evaporated. By adding acetone, a white solid was separated. After filtration, compound **5** was obtained as pale white solid (20.9 g, yield 86.7%). ^1^H NMR (DMSO-*d*_*6*_): *δ* 7.58 (s, 1H), 7.44 (d, *J* = 8.4 Hz, 1H), 7.37 (d, *J* = 8.4 Hz, 1H), 3.89 (s, 3H), 3.80 (t, *J* = 8.0 Hz, 2H), 3.61 (t, *J* = 7.2 Hz, 2H), 3.23–3.22 (m, 2H), 3.03 (t, *J* = 7.6 Hz, 2H).

### General procedure for the preparation of compound 6a

#### A-with different substituted acyl chlorides or sulfonyl chlorides or anhydrides

2-(7-Methoxy-3,4-dihydroisoquinolin-1-yl)ethanamine hydrochloride **(5)** (300 mg, 1.43 mmol) and K_2_CO_3_ (494 mg, 3.58 mmol) was dissolved in CH_2_Cl_2_ (10 mL) and DMF (2 mL), then appropriate substituted acyl chloride or sulfonyl chloride or anhydride (3.12 mmol) was added dropwise under ice bath. After stirring at room temperature for 10 h, the reaction mixture was diluted with CH_2_Cl_2_ (20 mL), then washed with H_2_O (20 mL). The organic layer was dried over Na_2_SO_4_, filtered and concentrated under reduced pressure and the residue was purified by chromatography or preparative TLC (eluent DCM/MeOH:80/1, v/v) to afford the desired crude product. The crude product was then dissolved in acetone, titrated with oxalic acid and the white solid were separated. After filtration, the oxalate form was obtained.

#### B- with different carboxylic acids

2-(7-Methoxy-3,4-dihydroisoquinolin-1-yl)ethanamine hydrochloride (**5**) (300 mg, 1.43 mmol) and EDCI (548 mg, 2.86 mmol) was dissolved in 10 mL pyridine, then carboxylic acid (1.72 mmol) was added. After stirring at room temperature for 10 h, the solvent was removed by evaporation and the residue was diluted with CH_2_Cl_2_ (20 mL), then washed with H_2_O (20 mL). The organic layer was dried over Na_2_SO_4_, filtered and concentrated under reduced pressure and the residue was purified by chromatography or preparative TLC (eluent DCM/MeOH: 80/1, v/v) to afford the desired crude product. The crude product was then dissolved in acetone, titrated with oxalic acid and the white solid were separated. After filtration, the oxalate form was obtained.

#### N-(2-(7-Methoxy-3,4-dihydroisoquinolin-1-yl) ethyl) acetamide oxalate (6a-1)

Following general procedure to obtain pure product as white solid 167 mg (yield: 39.9%). mp: 115–117 °C; ^1^H-NMR (D_2_O): 7.34 (d, 1H, *J* = 2.4 Hz, Ar-H), 7.31 (d, 1H, *J* = 8.4 Hz, Ar-H), 7.25 (dd, 1H, *J*_1_ = 8.4 Hz, *J*_2_ = 2.4 Hz, Ar-H), 3.78 (s, 3H, -OCH_3_), 3.73 (t, 2H, *J* = 8.0 Hz, -CH_2_-), 3.47 (t, 2H, *J* = 5.2 Hz, -CH_2_-), 3.17 (t, 2H, *J* = 4.8 Hz, -CH_2_-), 2.93 (t, 2H, *J* = 8.0 Hz, -CH_2_-), 1.72 (s, 3H, -CH_3_); ^13^C-NMR (D_2_O): *δ* 178.3, 174.3, 164.8, 158.4, 130.7, 129.9, 125.6, 123.3, 114.8, 55.8, 41.6, 37.5, 33.9, 23.6, 21.3; HRMS (TOF) m/z calcd. for C_14_H_18_N_2_O_2_ [M+H^+^]: 247.1450, found: 247.1447.

#### N-(2-(7-Methoxy-3,4-dihydroisoquinolin-1-yl) ethyl) propionamide oxalate (6a-2)

Following general procedure to obtain pure product as white solid 154 mg (yield: 30.7%). mp: 103–105 °C; ^1^H-NMR (DMSO-*d*_*6*_): *δ* 8.35 (br, s, 1H, -NH-), 7.60 (s, 1H, Ar-H), 7.41 (d, 1H, *J* = 8.4 Hz, Ar-H), 7.34 (d, 1H, *J* = 8.0 Hz, Ar-H), 3.87 (s, 3H, -OCH_3_), 3.73 (t, 2H, *J* = 7.6 Hz, -CH_2_-), 3.41 (t, 2H, *J* = 5.2 Hz, -CH_2_-), 3.31 (t, 2H, *J* = 4.8 Hz, -CH_2_-), 2.98 (t, 2H, *J* = 8.4 Hz, -CH_2_-), 1.99 (d, 2H, *J* = 8.4 Hz, -CH_2_-), 0.88 (t, 3H, *J* = 7.2 Hz, -CH_3_); ^13^C-NMR (DMSO-*d*_*6*_): *δ* 176.5, 173.6, 161.5, 158.5, 130.1, 129.6, 126.3, 122.1, 114.6, 55.8, 40.9, 37.3, 33.1 28.3, 23.4, 9.6; HRMS (TOF) m/z calcd. for C_15_H_20_N_2_O_2_ [M+H^+^]: 261.1603, found: 261.1580.

#### 2,2,2-Trifluoro-N-(2-(7-methoxy-3,4-dihydroisoquinolin-1-yl) ethyl) acetamideoxalate (6a-3)

Following general procedure to obtain pure product as white solid 89 mg (yield: 18.3%). mp: 41–43 °C; ^1^H-NMR (DMSO-*d*_*6*_): *δ* 8.32 (br, s, 1H, -NH-), 7.56 (s, 1H, Ar-H), 7.36 (d, 1H, *J* = 8.0 Hz, Ar-H), 7.17 (d, 1H, *J* = 8.0 Hz, Ar-H), 3.87 (s, 3H, -OCH_3_), 3.73 (t, 2H, *J* = 8.4 Hz, -CH_2_-), 3.41 (t, 2H, *J* = 5.2 Hz, -CH_2_-), 3.23 (t, 2H, *J* = 4.8 Hz, -CH_2_-), 2.79 (t, 2H, *J* = 8.0 Hz, -CH_2_-); ^13^C-NMR (DMSO-*d*_*6*_): *δ* 178.3, 173.2, 164.6, 158.5, 157.6, 130.2, 129.3, 128.7, 119.4, 116.8, 113.9, 55.7, 46.4, 37.4, 33.2; HRMS (TOF) m/z calcd. for C14H15F3N2O2 [M+H^+^]: 301.1164, found: 301.1245.

#### N-(2-(7-Methoxy-3,4-dihydroisoquinolin-1-yl) ethyl) cyclopropanecarboxamideoxalate (6a-4)

Following general procedure to obtain pure product as white solid 130 mg (yield: 35.9%). mp: >220 °C; ^1^H-NMR (DMSO-*d*_*6*_): *δ* 8.71 (br, s, 1H, -NH-), 7.72 (s, 1H, Ar-H), 7.53 (d, 1H, *J* = 8.0 Hz, Ar-H), 7.21 (d, 1H, *J* = 8.4 Hz, Ar-H), 3.83 (s, 3H, -OCH_3_), 3.73 (t, 2H, *J* = 7.6 Hz, -CH_2_-), 3.43 (t, 2H, *J* = 5.6 Hz, -CH_2_-), 3.28 (t, 2H, *J* = 4.8 Hz, -CH_2_-), 2.93 (t, 2H, *J* = 8.4 Hz, -CH_2_-), 1.32 (m, 1H, -CH-), 1.16 (m, 2H, -CH_2_-), 0.97 (m, 2H, -CH_2_-); ^13^C-NMR (DMSO-*d*_*6*_): *δ* 177.1, 173.8, 164.4, 158.5, 130.2, 129.4, 127.7, 121.5, 114.5, 55.6, 42.3, 37.4, 33.2, 25.5, 16.7, 8.7; HRMS (TOF) m/z calcd. for C_16_H_20_N_2_O_2_ [M+H^+^]: 273.1603, found: 273.1578.

#### 4-Chloro-N-(2-(7-methoxy-3,4-dihydroisoquinolin-1-yl) ethyl) benzamideoxalate (6a-5)

Following general procedure to obtain pure product as gray solid 160 mg (yield: 46.7%). mp: 90–92 °C; ^1^H-NMR (DMSO-*d*_*6*_): *δ* 8.95 (br, s, 1H, -NH-), 7.77 (d, 2H, *J* = 8.4 Hz, Ar-H), 7.57–7.52 (m, 3H, Ar-H), 7.38 (d, 1H, *J* = 8.0 Hz, Ar-H), 7.26 (d, 1H, *J* = 7.6 Hz, Ar-H), 3.82 (s, 3H, -OCH_3_), 3.72 (t, 2H, *J* = 8.4 Hz, -CH_2_-), 3.62 (t, 2H, *J* = 5.6 Hz, -CH_2_-), 3.30 (t, 2H, *J* = 4.8 Hz, -CH_2_-), 2.91 (t, 2H, *J* = 8.4 Hz, -CH_2_-); ^13^C-NMR (DMSO-*d*_*6*_): *δ* 174.7, 165.7, 162.8, 158.4, 136.1, 129.9, 129.4, 129.0, 128.3, 126.7, 121.0, 114.0, 55.6, 42.1, 37.9, 33.6, 23.6; HRMS (TOF) m/z calcd. for C_19_H_19_ClN_2_O_2_ [M+H^+^]: 343.1213, found: 343.1110.

#### 4-Methoxy-N-(2-(7-methoxy-3,4-dihydroisoquinolin-1-yl) ethyl) benzamideoxalate (6a-6)

Following general procedure to obtain pure product as pale yellow oil 192 mg (yield: 44.8%). ^1^H-NMR (DMSO-*d*_*6*_): *δ* 8.73 (br, s, 1H, -NH-), 7.74 (d, 2H, *J* = 8.4 Hz, Ar-H), 7.55 (d, 1H, *J* = 8.4 Hz, Ar-H), 7.36 (d, 1H, *J* = 7.6 Hz, Ar-H), 7.24 (dd, 1H, *J*_1_ = 8.4 Hz, *J*_2_ = 2.4 Hz, Ar-H), 6.96 (d, 2H, *J* = 8.0 Hz, Ar-H), 3.81 (s, 3H, -OCH_3_), 3.79 (s, 3H, -OCH_3_), 3.70 (t, 2H, *J* = 8.4 Hz, -CH_2_-), 3.60 (t, 2H, *J* = 5.2 Hz, -CH_2_-), 3.28 (t, 2H, *J* = 4.8 Hz, -CH_2_-), 2.89 (t, 2H, *J* = 8.0 Hz, -CH_2_-); ^13^C-NMR (DMSO-*d*_*6*_): *δ* 174.3, 166.2, 163.2, 161.6, 158.4, 129.9, 129.3, 128.9, 126.9, 126.1, 120.9, 113.9, 113.4, 55.6, 42.2, 37.8, 33.8, 30.7, 23.6; HRMS (TOF) m/z calcd. for C_20_H_22_N_2_O_3_ [M+H^+^]: 339.1709, found: 339.1609.

#### 2-Chloro-N-(2-(7-methoxy-3,4-dihydroisoquinolin-1-yl) ethyl) benzamide oxalate (6a-7)

Following general procedure to obtain pure product as white solid 274 mg (yield: 44.2%). mp: >220 °C; ^1^H-NMR (DMSO-*d*_*6*_): *δ* 9.07 (br, s, 1H, -NH-), 7.73 (t, 2H, *J* = 8.4 Hz, Ar-H), 7.51–7.45 (m, 3H, Ar-H), 7.35 (s, 1H, Ar-H), 7.19 (d, 1H, *J* = 8 Hz, Ar-H), 3.83 (s, 3H, -OCH_3_), 3.75 (t, 2H, *J* = 7.6 Hz, -CH_2_-), 3.59 (t, 2H, *J* = 5.6 Hz, -CH_2_-), 3.13 (t, 2H, *J* = 4.8 Hz, -CH_2_-), 2.97 (t, 2H, *J* = 8.0 Hz, -CH_2_-); ^13^C-NMR (DMSO-*d*_*6*_): *δ* 174.8, 165.5, 162.3, 158.3, 135.4, 132.8, 131.3, 129.2, 128.3, 126.7, 120.9, 115.5, 55.9, 44.7, 37.9, 33.9, 24.6; HRMS (TOF) m/z calcd. for C_19_H_19_ClN_2_O_2_ [M+H^+^]: 343.1213, found: 343.2206.

#### N-(2-(7-Methoxy-3,4-dihydroisoquinolin-1-yl) ethyl) -4-(trifluoromethyl) benzamideoxalate (6a-8)

Following general procedure to obtain pure product as yellow solid 78 mg (yield: 16.7%). mp: >220 °C; ^1^H-NMR (DMSO- *d*_*6*_): *δ* 8.93 (br, s, 1H, -NH-), 7.73 (d, 2H, *J* = 8.4 Hz, Ar-H), 7.63 (s, 1H, Ar-H), 7.36 (d, 2H, *J* = 8.0 Hz, Ar-H), 7.28 (d, 1H, *J* = 7.6 Hz, Ar-H), 7.21 (dd, 1H, *J*_1_ = 8.4 Hz, *J*_2_ = 2.8 Hz, Ar-H), 3.83 (s, 3H, -OCH_3_), 3.73 (t, 2H, *J* = 8.0 Hz, -CH_2_-), 3.58 (t, 2H, *J* = 5.6 Hz, -CH_2_-), 3.31 (t, 2H, *J* = 4.8 Hz, -CH_2_-), 2.93 (t, 2H, *J* = 8.4 Hz, -CH_2_-); ^13^C-NMR (DMSO-*d*_*6*_): *δ* 176.6, 166.7, 163.5, 158.5, 137.9, 133.6, 129.8, 129.7, 128.4, 127.1, 124.9, 123.3, 120.8, 114.3, 55.9, 45.1, 37.5, 34.3, 25.5. HRMS (TOF) m/z calcd. for C20H19F3N2O2 [M+H^+^]: 377.1477, found: 377.1724.

#### N-(2-(7-Methoxy-3,4-dihydroisoquinolin-1-yl) ethyl) furan-2-carboxamideoxalate (6a-9)

Following general procedure to obtain pure product as pale gray solid 497 mg (yield: 90.3%). mp: >220 °C; ^1^H-NMR (DMSO-*d*_*6*_): *δ* 8.85 (t, 1H, *J* = 5.6 Hz, -NH-), 7.82 (s, 1H, Ar-H), 7.62 (d, 1H, *J* = 2.0 Hz, Ar-H), 7.40 (d, 1H, *J* = 8.4 Hz, Ar-H), 7.31 (d, 1H, *J* = 8.4 Hz, Ar-H), 7.10 (d, 1H, *J* = 3.2 Hz, Ar-H), 6.60 (s, 1H, Ar-H), 3.83 (s, 3H, -OCH_3_), 3.75 (t, 2H, *J* = 7.6 Hz, -CH_2_-), 3.59 (t, 2H, *J* = 5.6 Hz, -CH_2_-), 3.37 (t, 2H, *J* = 6.0 Hz, -CH_2_-), 2.98 (t, 2H, *J* = 7.6 Hz, -CH_2_-); ^13^C-NMR (DMSO-*d*_*6*_): *δ* 176.3, 161.4, 158.5, 158.1, 154.5, 147.4, 145.2, 130.1, 129.5, 126.3, 114.5, 113.7, 111.8, 55.7, 37.3, 33.3, 33.4; HRMS (TOF) m/z calcd. for C_17_H_18_N_2_O_3_ [M+H^+^]: 299.1396, found: 299.2260.

#### N-(2-(7-Methoxy-3,4-dihydroisoquinolin-1-yl) ethyl)benzamide oxalate (6a-10)

Following general procedure to obtain pure product as pale yellow grease 362 mg (yield: 82.1%). ^1^H-NMR (DMSO-*d*_*6*_): *δ* 8.98 (br, s, 1H, -NH-), 7.95 (d, 1H, *J* = 8.4 Hz, Ar-H), 7.76 (d, 1H, *J* = 8.0 Hz, Ar-H), 7.61 (t, 1H, *J* = 7.6 Hz, Ar-H), 7.49 (t, 2H, *J* = 8.4 Hz, Ar-H), 7.41 (dd, 2H, *J*_1_ = 8.4 Hz, *J*_2_ = 2.8 Hz, Ar-H), 7.28 (d, 1H, *J* = 5.2 Hz, Ar-H), 3.83 (s, 3H, -OCH_3_), 3.73 (t, 2H, *J* = 8.0 Hz, -CH_2_-), 3.67 (t, 2H, *J* = 5.6 Hz, -CH_2_-), 3.31 (t, 2H, *J* = 4.8 Hz, -CH_2_-), 2.97 (t, 2H, *J* = 8 Hz, -CH_2_-); ^13^C-NMR (DMSO-*d*_*6*_): *δ* 175.2, 166.7, 163.5, 158.5, 137.9, 133.6, 129.8, 128.4, 127.1, 124.9, 123.3, 120.8, 114.3, 55.9, 45.1, 37.5, 34.3, 25.5. HRMS (TOF) m/z calcd. for C_19_H_20_N_2_O_2_ [M+H^+^]: 309.1603, found: 309.1607.

#### 4-(2-Bromoethyl) -N-(2-(7-methoxy-3,4-dihydroisoquinolin-1-yl) ethyl) benzamide oxalate (6a-11)

Following general procedure to obtain pure product as white powder 172 mg (yield: 23.8%). ^1^H-NMR (DMSO- *d*_*6*_): *δ* 7.89 (s, 4H, Ar-H), 7.40 (s, 1H, Ar-H), 7.34 (d, 1H, *J* = 8.4 Hz, Ar-H), 7.18 (d, 1H, *J* = 12.8 Hz, Ar-H), 3.94 (t, 2H, *J* = 8.0 Hz, -CH_2_-), 3.83 (s, 3H, -OCH_3_), 3.65 (t, 2H, *J* = 7.6 Hz, -CH_2_-), 3.28 (t, 2H, *J* = 5.6 Hz, -CH_2_-), 2.79 (t, 2H, *J* = 4.8 Hz,-CH_2_-), 2.55 (t, 2H, *J* = 8.0 Hz, -CH_2_-), 1.30 (t, 2H, *J* = 8.4 Hz, -CH_2_-); ^13^C-NMR (DMSO-*d*_*6*_): *δ* 174.3, 167.7, 161.9, 158.4, 142.9, 134.4, 131.5, 129.2, 129.1, 119.4, 112.3, 55.5, 43.3, 39.1, 33.5, 32.1, 31.6, 23.8; HRMS (TOF) m/z calcd. for C_21_H_23_BrN_2_O_2_ [M+H^+^]: 415.1021, found: 415.1307.

#### 4-Fluoro-N-(2-(7-methoxy-3,4-dihydroisoquinolin-1-yl) ethyl) benzamide oxalate (6a-12)

Following general procedure to obtain pure product as white powder 152 mg (yield: 25.5%). mp: 149–151 °C; ^1^H-NMR (DMSO-*d*_*6*_): *δ* 8.92 (br, s, 1H, -NH-), 7.80 (d, 2H, *J* = 8.4 Hz, Ar-H), 7.53–7.50 (m, 3H, Ar-H), 7.34 (d, 1H, *J* = 8.0 Hz, Ar-H), 7.22–7.20 (m, 1H, Ar-H), 3.81 (s, 3H, -OCH_3_), 3.69 (t, 2H, *J* = 7.2 Hz, -CH_2_-), 3.61 (d, 2H, *J* = 5.6 Hz, -CH_2_-), 3.24 (t, 2H, *J* = 4.8 Hz, -CH_2_-), 2.85 (t, 2H, *J* = 7.6 Hz, -CH_2_-); ^13^C-NMR (DMSO-*d*_*6*_): *δ* 172.7, 165.5, 162.9, 158.4, 136.1, 132.8, 129.8, 129.2, 129.1, 128.3, 127.2, 120.1, 113.4, 55.6, 42.9, 37.8, 33.8, 23.7; HRMS (TOF) m/z calcd. for C_19_H_19_FN_2_O_2_ [M+H^+^]: 327.1509, found: 327.1393.

#### 4-Isopropyl-N-(2-(7-methoxy-3,4-dihydroisoquinolin-1-yl) ethyl) benzamide oxalate (6a-13)

Following general procedure to obtain pure product as white powder 167 mg (yield: 39.9%). mp: 160–162^o^C; ^1^H-NMR (DMSO-*d*_*6*_): *δ* 9.04 (br, s, 1H, -NH-), 7.73 (d, 2H, *J* = 8.0 Hz, Ar-H), 7.66 (d, 1H, *J* = 2.0 Hz, Ar-H), 7.40 (d, 1H, *J* = 8.4 Hz, Ar-H), 7.30–7.27 (m, 3H, Ar-H), 3.81 (s, 3H, -OCH_3_), 3.71 (t, 2H, *J* = 7.6 Hz, -CH_2_-), 3.64 (d, 2H, *J* = 5.6 Hz, -CH_2_-), 3.44 (t, 2H, *J* = 4.8 Hz, -CH_2_-), 2.99 (d, 2H, *J* = 8.0 Hz, -CH_2_-), 2.95–2.90 (m, 1H, -CH-), 1.20 (d, 6H, *J* = 6.8 Hz, -CH_3_); ^13^C-NMR (DMSO-*d*_*6*_): *δ* 176.7, 166.7, 161.2, 158.5, 151.8, 131.4, 130.0, 129.5, 127.3, 126.4, 126.0, 122.1, 114.7, 55.8, 40.9, 38.0, 33.3, 33.1, 23.6, 23.4; HRMS (TOF) m/z calcd. for C_22_H_26_N_2_O_2_ [M+H^+^]: 351.2073, found: 351.1982.

#### N-(2-(7-Methoxy-3,4-dihydroisoquinolin-1-yl) ethyl)-1-(methylsulfonyl) methanamide oxalate (6a-14)

Following general procedure to obtain pure product as white powder 127 mg (yield: 18.6%). mp: 193–195 °C; ^1^H-NMR (DMSO-*d*_*6*_): *δ* 8.01 (br, s, 1H, -NH-), 7.73 (t, 1H, *J* = 8.0 Hz, Ar-H), 7.59 (d, 2H, *J* = 5.6 Hz, Ar-H), 7.42 (d, 2H, *J* = 8.4 Hz, Ar-H), 7.34 (m, 2H, Ar-H), 3.83–3.80 (d, 4H, *J* = 12.8 Hz, -CH_2_-), 3.72 (s, 3H, -OCH_3_), 3.29 (s, 1H, -CH_2_-), 3.17–3.13 (m, 1H, -CH_2_-), 2.98 (s, 1H, -CH_2_-), 2.35 (s, 3H, -CH_3_), 1.27–1.21 (m, 1H, -CH_2_-); ^13^C-NMR (DMSO-*d*_*6*_): *δ* 174.4, 166.7, 161.2, 158.5, 137.7, 131.6, 130.3, 127.9, 126.4, 119.6, 113.6, 55.8, 40.9, 38.0, 33.3, 33.1, 23.6, 23.4; HRMS (TOF) m/z calcd. for C_19_H_22_N_2_O_3_S [M+H^+^]: 359.1429, found: 359.1367.

#### 2,4-Dimethoxy-N-(2-(7-methoxy-3,4-dihydroisoquinolin-1-yl) ethyl) benzamide oxalate (6a-15)

Following general procedure to obtain pure product as white powder 286 mg (yield: 43.6%). mp: 85–87 °C; ^1^H-NMR (DMSO-*d*_*6*_): *δ* 8.43 (t, 1H, *J* = 5.6 Hz, -NH-), 7.72 (d, 1H, *J* = 8.4 Hz, Ar-H), 7.55 (d, 1H, *J* = 2.4 Hz, Ar-H), 7.35 (d, 1H, *J* = 7.6 Hz, Ar-H), 7.23 (d, 1H, *J* = 2.4 Hz, Ar-H), 6.60–6.56 (m, 2H, Ar-H), 3.86 (s, 3H, -OCH_3_), 3.80 (s, 3H, -OCH_3_), 3.79 (s, 3H, -OCH_3_), 3.72 (t, 2H, *J* = 7.6 Hz, -CH_2_-), 3.67 (d, 2H, *J* = 5.6 Hz, -CH_2_-), 3.31 (t, 2H, *J* = 4.8 Hz, -CH_2_-), 2.91 (t, 2H, *J* = 7.2 Hz, -CH_2_-); ^13^C-NMR (DMSO-*d*_*6*_): *δ* 174.9, 164.8, 163.0, 162.9, 158.7, 158.4, 132.4, 129.8, 129.2, 127.0, 120.9, 114.0, 105.5, 98.2, 55.8, 55.6, 55.4, 42.0, 37.7, 33.6, 30.6, 23.6; HRMS (TOF) m/z calcd. for C_21_H_24_N_2_O_4_ [M+H^+^]: 369. 1814, found: 369. 1677.

#### 4-Acetyl-N-(2-(7-methoxy-3,4-dihydroisoquinolin-1-yl) ethyl) benzamideoxalate (6a-16)

Following general procedure to obtain pure product as pale yellow grease 130 mg (yield: 20.6%). ^1^H-NMR (DMSO-*d*_*6*_): *δ* 9.24 (br, s, 1H, -NH-), 7.97 (d, 2H, *J* = 8.4 Hz, Ar-H), 7.89 (d, 2H, *J* = 8.0 Hz, Ar-H), 7.61 (s, 1H, Ar-H), 7.37 (d, 1H, *J* = 8.4 Hz, Ar-H), 7.25 (dd, 1H, *J*_1_ = 8.0 Hz, *J*_2_ = 1.6 Hz, Ar-H), 3.80 (s, 3H, -OCH_3_), 3.72 (t, 2H, *J* = 7.2 Hz, -CH_2_-), 3.65 (d, 2H, *J* = 5.6 Hz, -CH_2_-), 3.41 (s, 2H, -CH_2_-), 2.96 (t, 2H, *J* = 7.2 Hz, -CH_2_-), 2.59 (s, 3H, -CH_3_); ^13^C-NMR (DMSO-*d*_*6*_): *δ* 197.7, 175.9, 166.0, 163.0, 161.6, 158.4, 138.6, 137.6, 130.0, 128.0, 126.5, 121.7, 114.4, 55.7, 51.4, 48.54, 41.4, 38.0, 33.1, 26.9, 23.5; HRMS (TOF) m/z calcd. for C_21_H_22_N_2_O_3_ [M+H^+^]: 351.1709, found: 351.1729.

#### 2-(1H-Indol-2-yl)-N-(2-(7-methoxy-3,4-dihydroisoquinolin-1-yl)ethyl)acetamide oxalate(6a-17)

Following general procedure to obtain pure product as pale yellow grease 160 mg (yield: 24.0%). ^1^H-NMR (DMSO-*d*_*6*_): *δ* 8.44 (d, 1H, *J* = 5.6 Hz, -NH-), 7.85 (s, 3H, Ar-H), 7.35 (s, 1H, Ar-H), 7.30 (d, 1H, *J* = 8.4 Hz, Ar-H), 7.13 (d, 1H, *J* = 8.0 Hz, Ar-H), 7.05–6.97 (m, 2H, Ar-H), 3.88 (t, 2H, *J* = 8.4 Hz, -CH_2_-), 3.79 (s, 3H, -OCH_3_), 3.69 (t, *J* = 5.6 Hz, -CH_2_-), 3.61 (t, 2H, *J* = 4.8 Hz, -CH_2_-), 3.23 (t, 2H, *J* = 7.2 Hz, -CH_2_-), 2.74 (t, 2H, *J* = 7.6 Hz, -CH_2_-); ^13^C-NMR (DMSO-*d*_*6*_): *δ* 174.9, 166.0, 161.6, 157.6, 136.6, 129.8, 129.3, 128.8, 126.5, 121.7, 119.9, 114.4, 111.5, 55.7, 48.9, 41.4, 36.9, 33.1, 24.8; HRMS (TOF) m/z calcd. for C_21_H_22_N_2_O_3_ [M+H^+^]: 362.1869, found: 362.1826.

#### 4-(tert-Butyl)-N-(2-(7-methoxy-3,4-dihydroisoquinolin-1-yl)ethyl)benzamide oxalate (6a-18)

Following general procedure to obtain pure product as pale yellow solid 144 mg (yield: 22.2%). mp: 63–65 °C; ^1^H-NMR (DMSO-*d*_*6*_): *δ* 8.87 (br, s, 1H, -NH-), 7.72 (d, 2H, *J* = 8.0 Hz, Ar-H), 7.60 (s, 1H, Ar-H), 7.44 (d, 2H, *J* = 8.0 Hz, Ar-H), 7.38 (d, 1H, *J* = 8.4 Hz, Ar-H), 7.26 (dd, 1H, *J*_1_ = 8.4 Hz, *J*_2_ = 1.6 Hz, Ar-H), 3.81 (s, 3H, -OCH_3_), 3.72 (t, 2H, *J* = 7.6 Hz, -CH_2_-), 3.63 (d, 2H, *J* = 5.2 Hz, -CH_2_-), 3.38 (t, 2H, *J* = 4.8 Hz, -CH_2_-), 2.93 (t, 2H, *J* = 7.6 Hz, -CH_2_-), 1.28 (s, 9H, -CH_3_); ^13^C-NMR (DMSO-*d*_*6*_): *δ* 175.1, 166.6, 163.3, 158.5, 154.0, 131.2, 129.9, 129.4, 127.0, 126.7, 124.9, 121.2, 114.2, 55.6, 41.8, 37.9, 34.5, 33.6, 30.9, 23.6. HRMS (TOF) m/z calcd. for C_23_H_28_N_2_O_2_ [M+H^+^]: 365.2229, found: 365.2178.

#### 4-Cyano-N-(2-(7-methoxy-3,4-dihydroisoquinolin-1-yl)ethyl)benzamide oxalate(6a-19)

Following general procedure to obtain pure product as pale yellow solid 170 mg (yield: 28.1%). mp: 180–182 °C; ^1^H-NMR (DMSO-*d*_*6*_): *δ* 9.43 (br, s, 1H, -NH-), 7.94 (dd, 4H, *J*_1_ = 16.4 Hz, *J*_2_ = 8.4 Hz, Ar-H), 7.62 (d, 1H, *J* = 2.0 Hz, Ar-H), 7.39 (d, 1H, *J* = 8.4 Hz, Ar-H), 7.28 (dd, 1H, *J*_1_ = 8.4 Hz, *J*_2_ = 2.4 Hz, Ar-H), 3.81 (s, 3H, -OCH_3_), 3.74 (t, 2H, *J* = 7.6 Hz, -CH_2_-), 3.66 (d, 2H, *J* = 5.6 Hz, -CH_2_-), 3.45 (t, 2H, *J* = 5.6 Hz, -CH_2_-), 2.99 (t, 2H, *J* = 7.2 Hz, -CH_2_-); ^13^C-NMR (DMSO-*d*_*6*_): *δ* 176.5, 165.3, 161.4, 158.4, 137.7, 132.3, 130.1, 129.6, 128.1, 126.3, 122.1, 118.3, 114.7, 113.6, 55.8, 41.0, 38.1, 32.9, 23.4. HRMS (TOF) m/z calcd. for C_20_H_19_N_3_O_2_ [M+H^+^]: 334.1556, found: 334.1392.

#### 2-((2-(7-Methoxy-3,4-dihydroisoquinolin-1-yl)ethyl)carbamoyl)phenylacetate oxalate(6a-20)

Following general procedure to obtain pure product as white solid powder 243 mg (yield: 37.2%). mp: > 220 °C; ^1^H-NMR (DMSO-*d*_*6*_): *δ* 9.49 (br, s, 1H, -NH-), 7.88 (t, 1H, *J* = 8.4 Hz, Ar-H), 7.82 (s, 1H, Ar-H), 7.64 (s, 1H, Ar-H), 7.43–7.35 (m, 2H, Ar-H), 7.27 (d, 1H, *J* = 8.0 Hz, Ar-H), 6.91–6.83 (m, 1H, Ar-H), 3.83 (s, 3H, -OCH_3_), 3.75–3.70 (m, 2H, -CH_2_-), 3.48 (t, 2H, *J* = 5.6 Hz, -CH_2_-), 3.16 (s, 3H, -CH_3_), 3.00 (t, 2H, *J* = 4.8 Hz, -CH_2_-), 1.91 (s, 2H, *J* = 8.4 Hz, -CH_2_-); ^13^C-NMR (DMSO-*d*_*6*_): *δ* 172.3, 165.6, 164.0, 161.4, 158.4, 148.4, 132.6, 130.2, 129.4, 128.7, 127.7, 122.4, 118.8, 113.7, 55.6, 44.2, 37.9, 34.2, 24.5, 21.0. HRMS (TOF) m/z calcd. for C_21_H_22_N_2_O_4_ [M+H^+^]: 367.2416, found: 367.1658.

#### 2-Chloro-N-(2-(7-methoxy-3,4-dihydroisoquinolin-1-yl)ethyl)nicotinamide oxalate(6a-21)

Following general procedure to obtain pure product as pale yellow solid powder 208 mg (yield: 37.2%). mp: 91–93 °C; ^1^H-NMR (DMSO-*d*_*6*_): *δ* 9.26 (br, s, 1H, -NH-), 8.54 (d, 1H, *J* = 4.8 Hz, Ar-H), 7.75 (s, 1H, Ar-H), 7.69 (d, 1H, *J* = 4.8 Hz, Ar-H), 7.48 (s, 1H, Ar-H), 7.35 (d, 1H, *J* = 8.4 Hz, Ar-H), 7.22 (d, 1H, *J* = 8.0 Hz, Ar-H), 3.82 (s, 3H, -OCH_3_), 3.70 (t, 2H, *J* = 7.6 Hz, -CH_2_-), 3.63 (d, 2H, *J* = 5.6 Hz, -CH_2_-), 3.26 (t, 2H, *J* = 5.6 Hz, -CH_2_-), 2.86 (t, 2H, *J* = 7.6 Hz, -CH_2_-); ^13^C-NMR (DMSO-*d*_*6*_): *δ* 172.7, 163.6, 162.9, 158.4, 150.8, 150.6, 144.5, 129.8, 129.2, 127.1, 121.8, 120.7, 120.1, 113.5, 55.6, 42.8, 37.8, 33.4, 30.7, 23.7. HRMS (TOF) m/z calcd. for C_18_H_18_ClN_3_O_2_ [M+H^+^]: 344.1166, found: 344.1169.

#### N-(2-(7-Methoxy-3,4-dihydroisoquinolin-1-yl)ethyl)-3-methylbenzamide oxalate(6a-22)

Following general procedure to obtain pure product as pale yellow solid powder 267 mg (yield: 45.2%). mp: > 220 °C; ^1^H-NMR (DMSO-*d*_*6*_): *δ* 9.04 (t, 1H, *J* = 5.6 Hz, -NH-), 7.74 (t, 1H, *J* = 8.4 Hz, Ar-H), 7.65 (d, 1H, *J* = 5.6 Hz, Ar-H), 7.57 (d, 2H, *J* = 7.6 Hz, Ar-H), 7.40 (t, 1H, *J* = 4.8 Hz, Ar-H), 7.29 (t, 2H, *J* = 8.4 Hz, Ar-H), 3.81 (s, 3H, -OCH_3_), 3.72 (t, 2H, *J* = 7.2 Hz, -CH_2_-), 3.64 (t, 2H, *J* = 5.6 Hz, -CH_2_-), 3.43 (t, 2H, *J* = 4.8 Hz, -CH_2_-), 2.99 (t, 2H, *J* = 8.0 Hz, -CH_2_-), 2.32 (s, 3H, -CH_3_); ^13^C-NMR (DMSO-*d*_*6*_): *δ* 176.8, 166.9, 161.1, 158.5, 137.4, 133.8, 131.8, 130.0, 129.5, 128.0, 127.7, 126.4, 124.3, 122.2, 114.7, 55.7, 40.98, 38.0, 32.9, 23.4, 20.9. HRMS (TOF) m/z calcd. for C_20_H_22_N_2_O_2_ [M+H^+^]: 323.1760, found: 323.1741.

#### N-(2-(7-Methoxy-3,4-dihydroisoquinolin-1-yl)ethyl)nicotinamide oxalate(6a-23)

Following general procedure to obtain pure product as pale yellow solid powder 97 mg (yield: 16.9%). mp: 57–59 °C; ^1^H-NMR (DMSO- *d*_*6*_): *δ* 9.18 (br, s, 1H, -NH-), 8.91 (s, 1H, Ar-H), 8.68 (d, 1H, *J* = 4.0 Hz, Ar-H), 8.12 (d, 1H, *J* = 7.6 Hz, Ar-H), 7.59 (s, 1H, Ar-H), 7.49–7.46 (m, 1H, Ar-H), 7.38 (d, 1H, *J* = 8.0 Hz, Ar-H), 7.27 (dd, 1H, *J*_1_ = 8.4 Hz, *J*_2_ = 2.4 Hz, Ar-H), 3.81 (s, 3H, -OCH_3_), 3.77–3.71 (m, 2H, -CH_2_-), 3.65 (d, 2H, *J* = 5.6 Hz, -CH_2_-), 3.37 (t, 2H, *J* = 4.8 Hz, -CH_2_-), 2.95 (t, 2H, *J* = 7.6 Hz, -CH_2_-); ^13^C-NMR (DMSO-*d*_*6*_): *δ* 165.3, 162.0, 158.4, 151.9, 148.3, 134.8, 130.0, 129.5, 129.4, 126.6, 123.3, 121.4, 114.3, 55.7, 41.7, 37.8, 33.3, 30.7, 23.5. HRMS (TOF) m/z calcd. for C_18_H_19_N_3_O_2_ [M+H^+^]: 310.1556, found: 310.1578.

#### N-(2-(7-Methoxy-3,4-dihydroisoquinolin-1-yl)ethyl)picolinamide oxalate(6a-24)

Following general procedure to obtain pure product as pale yellow solid powder 158 mg (yield: 27.7%). mp: 95–97 °C; ^1^H-NMR (DMSO-*d*_*6*_): *δ* 9.14 (t, 1H, *J* = 6.0 Hz,-NH-), 8.61 (d, 1H, *J* = 4.4 Hz, Ar-H), 7.95 (dd, 2H, *J*_1_ = 12.8 Hz, *J*_2_ = 7.2 Hz, Ar-H), 7.59–7.57 (m, 1H, Ar-H), 7.51 (d, 1H, *J* = 2.0 Hz, Ar-H), 7.31 (d, 1H, *J* = 8.4 Hz, Ar-H), 7.17 (dd, 1H, *J*_1_ = 8.4 Hz, *J*_2_ = 2.4 Hz, Ar-H), 3.79 (s, 3H, -OCH_3_), 3.72–3.65 (m, 4H, -CH_2_-), 3.29 (t, 2H, *J* = 6.0 Hz, -CH_2_-), 2.87 (t, 2H, *J* = 7.2 Hz, -CH_2_-); ^13^C-NMR (DMSO-*d*_*6*_): *δ* 173.6, 164.2, 163.4, 158.3, 149.5, 148.3, 137.7, 129.8, 129.1, 127.1, 126.5, 121.8, 120.5, 113.6, 55.5, 42.5, 37.5, 33.6, 23.7. HRMS (TOF) m/z calcd. for C_18_H_19_N_3_O_2_ [M+H^+^]: 310.1556, found: 310.1546.

#### N-(2-(7-Methoxy-3,4-dihydroisoquinolin-1-yl)ethyl)-4-methylbenzamide oxalate(6a-25)

Following general procedure to obtain pure product as pale yellow solid powder 258 mg (yield: 43.7%). mp: 208–210 °C; ^1^H-NMR (DMSO-*d*_*6*_): *δ* 8.92 (t, 1H, *J* = 5.2 Hz, -NH-), 7.69 (d, 2H, *J* = 8.0 Hz, Ar-H), 7.64 (d, 1H, *J* = 2.0 Hz, Ar-H), 7.40 (d, 1H, *J* = 8.4 Hz, Ar-H), 7.29 (dd, 1H, *J*_1_ = 8.4 Hz, *J*_2_ = 2.4 Hz, Ar-H), 7.23 (d, 2H, *J* = 8.0 Hz, Ar-H), 3.82 (s, 3H, -OCH_3_), 3.71 (t, 2H, *J* = 8.0 Hz, -CH_2_-), 3.63 (t, 2H, *J* = 5.6 Hz, -CH_2_-), 3.39 (t, 2H, *J* = 5.6 Hz, -CH_2_-), 2.97 (t, 2H, *J* = 7.6 Hz, -CH_2_-), 2.34 (s, 3H, -CH_3_); ^13^C-NMR (DMSO-*d*_*6*_): *δ* 175.1, 166.6, 161.8, 158.5, 141.2, 131.04, 130.0, 129.5, 128.7, 127.1, 126.5, 121.8, 114.2, 55.7, 41.3, 37.9, 33.3, 30.9, 23.5, 20.9. HRMS (TOF) m/z calcd. for C_20_H_22_N_2_O_2_ [M+H^+^]: 323.1760, found: 323.1693.

#### N-(2-(7-Methoxy-3,4-dihydroisoquinolin-1-yl)ethyl)quinoline-2-carboxamide oxalate(6a-26)

Following general procedure to obtain pure product as pale yellow oil 210 mg (yield: 32.6%). ^1^H-NMR (DMSO-*d*_*6*_): *δ* 9.21 (t, 1H, *J* = 4.8 Hz, -NH-), 8.65 (d, 1H, *J* = 4.4 Hz, Ar-H), 8.07 (d, 2H, *J* = 8.4 Hz, Ar-H), 7.68 (d, 1H, *J* = 5.6 Hz, Ar-H), 7.55–7.51 (m, 2H, Ar-H), 7.42 (d, 1H, *J* = 8.0 Hz, Ar-H), 7.35 (d, 1H, *J* = 2.0 Hz, Ar-H), 7.33 (d, 1H, *J* = 2.4 Hz, Ar-H), 3.86 (s, 3H, -OCH_3_), 3.75 (t, 2H, *J* = 8.4 Hz, -CH_2_-), 3.59 (t, 2H, *J* = 5.6 Hz, -CH_2_-), 3.45 (t, 2H, *J* = 4.8 Hz, -CH_2_-), 2.98 (t, 2H, *J* = 7.6 Hz, -CH_2_-); ^13^C-NMR (DMSO-*d*_*6*_): *δ* 173.6, 164.2, 163.4, 158.3, 149.5, 148.3, 137.7, 129.8, 129.1, 127.1, 126.5, 121.8, 120.5, 113.6, 55.5, 42.5, 37.5, 33.6, 23.7. HRMS (TOF) m/z calcd. for C_22_H_21_N_3_O_2_ [M+H^+^]: 360.1712, found: 360.1797.

#### 2-Bromo-5-chloro-N-(2-(7-methoxy-3,4-dihydroisoquinolin-1-yl)ethyl)benzamide oxalate(6a-27)

Following general procedure to obtain pure product as colorless oil 152 mg (yield: 20.8%). ^1^H-NMR (DMSO-*d*_*6*_): *δ* 8.95 (br, s, 1H, -NH-), 7.67 (dd, 1H, *J*_1_ = 8.0 Hz, *J*_2_ = 4.8 Hz, Ar-H), 7.60 (s, 1H, Ar-H), 7.41 (d, 1H, *J* = 8.4 Hz, Ar-H), 7.32 (d, 1H, *J* = 8.0 Hz, Ar-H), 7.24 (t, 2H, *J* = 8.4 Hz, Ar-H), 3.86 (s, 3H, -OCH_3_), 3.77 (t, 2H, *J* = 7.6 Hz, -CH_2_-), 3.60 (t, 2H, *J* = 5.6 Hz, -CH_2_-), 3.37 (t, 2H, *J* = 4.8 Hz,-CH_2_-), 2.94 (t, 2H, *J* = 6.8 Hz, -CH_2_-); ^13^C-NMR (DMSO-*d*_*6*_): *δ* 166.4, 163.3, 162.1, 159.6, 158.5, 140.0, 134.7, 130.1, 129.5, 126.6, 121.1, 118.3, 116.2, 114.3, 113.6, 55.7, 41.9, 37.7, 33.3, 23.5; HRMS (TOF) m/z calcd. for C_19_H_18_BrClN_2_O_2_ [M+H^+^]: 421.0318, found: 421.0287.

#### 2-Bromo-N-(2-(7-methoxy-3,4-dihydroisoquinolin-1-yl)ethyl)-5-(trifluoromethyl)benzamide oxalate (6a-28)

Following general procedure to obtain pure product as colorless oil 167 mg (yield: 39.9%). ^1^H-NMR (DMSO-*d*_*6*_): *δ* 9.06 (br, s, 1H, -NH-), 7.89 (d, 1H, *J* = 8.4 Hz, Ar-H), 7.70 (d, 2H, *J* = 5.6 Hz, Ar-H), 7.41 (d, 2H, *J* = 8.0 Hz, Ar-H), 7.40–7.32 (m, 3H, Ar-H), 3.85 (s, 3H, -OCH_3_), 3.78 (t, 2H, *J* = 8.4 Hz, -CH_2_-), 3.64 (t, 2H, *J* = 5.6 Hz, -CH_2_-), 3.39 (t, 2H, *J* = 4.8 Hz, -CH_2_-), 2.94 (t, 2H, *J* = 7.6 Hz, -CH_2_-); ^13^C-NMR (DMSO-*d*_*6*_): *δ* 174.8, 166.4, 163.1, 158.5, 151.5, 139.2, 134.0, 132.4, 130.0, 129.5, 126.7, 123.7, 121.1, 118.1, 115.9, 114.2, 55.7, 42.0, 37.9, 33.2, 23.5; HRMS (TOF) m/z calcd. for C_20_H_18_BrF_3_N_2_O_2_ [M+H^+^]: 455.0582, found: 455.0606.

#### 5-Bromo-N-(2-(7-methoxy-3,4-dihydroisoquinolin-1-yl)ethyl)nicotinamide oxalate(6a-29)

Following general procedure to obtain pure product as white solid 109 mg (yield: 15.9%). mp: 185–187 °C; ^1^H-NMR (DMSO-*d*_*6*_): *δ* 9.22 (s, 1H, -NH-), 8.85 (dd, 2H, *J*_1_ = 12.8 Hz, *J*_2_ = 4.8 Hz, Ar-H), 8.29 (s, 1H, Ar-H), 7.52 (s, 1H, Ar-H), 7.36 (d, 1H, *J* = 8.0 Hz, Ar-H), 7.23 (dd, 1H, *J*_1_ = 8.4 Hz, *J*_2_ = 2.4 Hz, Ar-H), 3.81 (s, 3H, -OCH_3_), 3.72 (t, 2H, *J* = 8.4 Hz, -CH_2_-), 3.64 (d, 2H, *J* = 5.6 Hz, -CH_2_-), 3.30 (s, 2H, -CH_2_-), 2.90 (t, 2H, *J* = 7.6 Hz, -CH_2_-); ^13^C-NMR (DMSO-*d*_*6*_): *δ* 163.8, 163.2, 158.4, 152.5, 146.9, 137.2, 131.0, 129.9, 129.3, 126.9, 120.6, 119.9, 113.9, 55.6, 42.3, 37.9, 33.3, 23.6; HRMS (TOF) m/z calcd. for C_18_H_18_BrN_3_O_2_ [M+H^+^]: 388.0661, found: 388.0674.

#### 5-Bromo-2-chloro-N-(2-(7-methoxy-3,4-dihydroisoquinolin-1-yl)ethyl)nicotinamide oxalate (6a-30)

Following general procedure to obtain pure product as white solid 220 mg (yield: 30.0%). mp: 168–170 °C; ^1^H-NMR (DMSO-*d*_*6*_): *δ* 9.02 (s, 1H, -NH-), 8.64 (d, 1H, *J* = 8.4 Hz, Ar-H), 8.04 (d, 1H, *J* = 5.6 Hz, Ar-H), 7.46 (s, 1H, Ar-H), 7.35 (d, 1H, *J* = 8.0 Hz, Ar-H), 7.24 (d, 1H, *J* = 8.4 Hz, Ar-H), 3.84 (s, 3H, -OCH_3_), 3.71 (t, 2H, *J* = 8.0 Hz, -CH_2_-), 3.59 (d, 2H, *J* = 5.6 Hz, -CH_2_-), 3.23 (s, 2H, -CH_2_-), 2.83 (t, 2H, *J* = 7.2 Hz, -CH_2_-); ^13^C-NMR (DMSO-*d*_*6*_): *δ* 163.9, 163.2, 158.4, 150.9, 145.5, 140.2, 133.7, 129.8, 129.2, 127.4, 119.5, 118.7, 113.2, 55.6, 43.3, 37.5, 33.5, 23.8; HRMS (TOF) m/z calcd. for C_18_H_17_BrClN_3_O_2_ [M+H^+^]: 422.0271, found: 422.0247.

#### 6-Bromo-N-(2-(7-methoxy-3,4-dihydroisoquinolin-1-yl)ethyl)nicotinamide oxalate (6a-31)

Following general procedure to obtain pure product as white solid 227 mg (yield: 33.2%). ^1^H-NMR (DMSO-*d*_*6*_): *δ* 9.28 (s, 1H, -NH-), 8.71 (s, 1H, Ar-H), 8.05 (d, 1H, *J* = 8.4 Hz, Ar-H), 7.75 (d, 1H, *J* = 8.0 Hz, Ar-H), 7.56 (s, 1H, Ar-H), 7.37 (d, 1H, *J* = 7.6 Hz, Ar-H), 7.25 (d, 1H, *J* = 8.0 Hz, Ar-H), 3.82 (s, 3H, -OCH_3_), 3.73 (t, 2H, *J* = 7.6 Hz, -CH_2_-), 3.64 (d, 2H, *J* = 4.8 Hz, -CH_2_-), 3.35 (s, 2H, -CH_2_-), 2.92 (t, 2H, *J* = 8.0 Hz, -CH_2_-); ^13^C-NMR (DMSO-*d*_*6*_): *δ* 174.8, 164.3, 163.1, 158.4, 149.3, 144.0, 138.1, 130.0, 129.4, 129.1, 127.8, 126.6, 121.1, 114.1, 55.7, 41.9, 37.84, 33.2, 23.5; HRMS (TOF) m/z calcd. for C_18_H_18_BrN_3_O_2_ [M+H^+^]: 388.0661, found: 388.0643.

#### N-(2-(7-Methoxy-3,4-dihydroisoquinolin-1-yl)ethyl)-4-methylbenzamide oxalate (6a-32)

Following general procedure to obtain pure product as colorless oil 281 mg (yield: 54.8%). ^1^H-NMR (DMSO-*d*_*6*_): *δ* 8.01 (br, s, 1H, -NH-), 7.82 (s, 1H, Ar-H), 7.73 (dd, 1H, *J*_1_ = 12.8 Hz, *J*_2_ = 4.8 Hz, Ar-H), 7.60 (d, 1H, *J* = 8.4 Hz, Ar-H), 7.40 (t, 2H, *J* = 15.6 Hz, Ar-H), 7.34 (t, 2H, *J* = 8.4 Hz, Ar-H), 3.81 (d, 2H, *J* = 7.6 Hz, -CH_2_-), 3.72 (s, 3H, -OCH_3_), 3.29 (s, 1H, -CH_2_-), 3.17–3.13 (m, 1H, -CH_2_-), 2.98 (s, 1H, -CH_2_-), 2.35 (d, 2H, *J* = 4.8 Hz, -CH_2_-), 2.28 (s, 3H, -CH_3_), 1.27–1.21 (m, 1H, -CH_2_-); ^13^C-NMR(DMSO-*d*_*6*_): *δ* 176.0, 161.8, 160.1, 158.5, 142.9, 137.0, 129.7, 126.4, 125.6, 122.2 114.6, 113.6, 112.5, 55.7, 33.2, 23.4, 20.8, 13.7; HRMS (TOF) m/z calcd. for C_19_H_22_N_2_O_3_S [M+H^+^]: 359.1429, found: 359.1367.

#### 4-Fluoro-N-(2-(7-methoxy-3,4-dihydroisoquinolin-1-yl)ethyl)benzamide oxalate (6a-33)

Following general procedure to obtain pure product as colorless oil 176 mg (yield: 34.0%). ^1^H-NMR (DMSO-*d*_*6*_): *δ* 8.05 (s, 1H, -NH-), 7.84 (s, 2H, Ar-H), 7.42 (t, 2H, *J* = 8.4 Hz, Ar-H), 7.33 (d, 1H, *J* = 4.8 Hz, Ar-H), 7.28 (s, 1H, Ar-H), 7.20 (t, 1H, *J* = 8.0 Hz, Ar-H), 3.82 (s, 3H, -OCH_3_), 3.68 (t, 2H, *J* = 7.6 Hz, -CH_2_-), 3.13 (s, 2H, -CH_2_-), 2.80 (s, 2H, -CH_2_-), 1.25 (d, 2H, *J* = 8.4 Hz, -CH_2_-); ^13^C-NMR (DMSO-*d*_*6*_): *δ* 166.3, 164.1, 162.6, 158.3, 139.5, 131.7, 130.2, 129.9, 129.1, 118.2, 116.4, 113.7, 55.6, 43.3, 37.6, 33.2, 25.4; HRMS (TOF) m/z calcd. for C_18_H_19_FN_2_O_3_S [M+H^+^]: 363.1179, found: 363.1084.

#### 2,6-Difluoro-N-(2-(7-methoxy-3,4-dihydroisoquinolin-1-yl)ethyl)benzamide oxalate (6a-34)

Following general procedure to obtain pure product as colorless oil 114 mg (yield: 20.9%). ^1^H-NMR (DMSO-*d*_*6*_): *δ* 8.51 (s, 1H, -NH-), 7.85 (s, 3H, Ar-H), 7.45 (s, 1H, Ar-H), 7.35 (t, 1H, *J* = 15.6 Hz, Ar-H), 7.22 (t, 1H, *J* = 8.0 Hz, Ar-H), 3.91 (t, 2H, *J* = 6.0 Hz, -CH_2_-), 3.80 (s, 3H, -OCH_3_), 3.66 (t, 2H, *J* = 12.8 Hz, -CH_2_-), 3.33 (t, 2H, *J* = 8.4 Hz, -CH_2_-), 3.17 (t, 1H, *J* = 5.6 Hz, -CH_2_-), 2.83 (t, 2H, *J* = 8.4 Hz, -CH_2_-); ^13^C-NMR (DMSO-*d*_*6*_): *δ* 165.5, 163.9, 162.1, 159.6, 139.6, 134.4, 129.9, 129.1, 118.1, 116.3, 114.3, 113.6, 112.5, 55.7, 41.9, 37.7, 33.34 23.5; HRMS (TOF) m/z calcd. for C_18_H_18_F_2_N_2_O_3_S [M+H^+^]: 381.1084, found: 381.1124.

### General procedure for the preparation of compound 6b

Into a 50 mL three-neck flask, 2-(7-methoxy-3,4-dihydroisoquinolin-1-yl)ethanamine hydrochloride **(5)** 300 mg(1.43 mmol, 1.0 eq), potassium carbonate 279 mg(1.72 mmol, 1.2 eq), DMF 2 mL and DCM 10 mL were added in order. Then the mixture was stirred in ice bath for 10 min. CDI(1.72 mmol, 1.2 eq) was added in three portions. After stirring at room temperature for 10 h, the reaction mixture was diluted with CH_2_Cl_2_ (20 mL), then washed with H_2_O. The organic layer was dried over Na_2_SO_4_, filtered and concentrated under reduced pressure and the residue was purified by chromatography or preparative TLC (eluent DCM/MeOH: 80/1, v/v) to afford the crude product. The crude product was dissolved in acetone, titrated with oxalic acid and the white solid were separated. After filtration, their oxalate form was obtained.

#### 1-Cyclopropyl-3-(2-(7-methoxy-3,4-dihydroisoquinolin-1-yl)ethyl)urea oxalate(6b-1)

Following general procedure to obtain pure product as white solid 78 mg (yield: 18.9%). ^1^H-NMR (DMSO-*d*_*6*_): *δ* 8.93 (s, 1H, -NH-), 8.59 (d, 1H, *J* = 4.8 Hz, -NH-), 7.51 (s, 1H, Ar-H), 7.35 (d, 1H, *J* = 5.6 Hz, Ar-H), 7.17 (d, 1H, *J* = 4.8 Hz, Ar-H), 4.09 (t, 2H, *J* = 2.4 Hz, -CH_2_-), 3.84 (s, 3H, -OCH_3_), 3. 73 (t, 2H, *J* = 5.6 Hz, -CH_2_-), 3.31–3.24 (m, 1H, -CH-), 2.95 (t, 2H, *J* = 7.6 Hz, -CH_2_-), 2.70–1.60 (m, 2H, -CH_2_-), 0.86 (s, 1H,-CH_2_-), 0.64 (s, 1H,-CH_2_-), 0.52 (s, 1H,-CH_2_-), 0.27 (s, 1H,-CH_2_-); ^13^C-NMR (DMSO-*d*_*6*_): *δ*164.0, 161.2, 159.9, 158.7, 129.6, 128.8, 119.3, 113.0, 99.9, 55.7, 46.2, 37.6, 35.7, 28.8, 25.5, 6.9; HRMS (TOF) *m/z* calcd. for C_16_H_21_N_3_O_2_ [M+H^+^]: 288.1712, found: 288.1695.

#### 1-(3-Chlorophenyl)-3-(2-(7-methoxy-3,4-dihydroisoquinolin-1-yl)ethyl)urea oxalate(6b-2)

Following general procedure to obtain pure product as brown solid 253 mg (yield: 39.5%). mp: 185–187 °C; ^1^H-NMR (DMSO-*d*_*6*_): *δ* 8.88 (s, 1H, -NH-), 8.57 (d, 1H, *J* = 4.4 Hz, -NH-), 7.58 (s, 3H, Ar-H), 7.49 (d, 1H, *J* = 1.6 Hz, Ar-H), 7.34 (d, 1H, *J* = 8.4 Hz, Ar-H), 7.16–7.11 (m, 2H, Ar-H), 4.08 (t, 2H, *J* = 6.8 Hz, -CH_2_-), 4.03–3.98 (m, 1H, -CH_2_-), 3.83 (s, 3H, -OCH_3_), 3.80–3.70 (m, 2H, -CH_2_-), 2.96 (t, 2H, *J* = 8.0 Hz, -CH_2_-), 2.08 (s, 1H, -CH_2_-); ^13^C-NMR (DMSO-*d*_*6*_): *δ*164.4, 163.6, 158.5, 155.8, 152.5, 134.4, 130.2, 129.4, 128.7, 127.5, 119.5, 118.9, 117.4, 116.5, 111.0, 99.8, 55.5, 40.4, 30.7, 25.6; HRMS (TOF) *m/z* calcd. for C_19_H_20_ClN_3_O_2_ [M+H^+^]: 358.1322, found: 358.1368.

#### 1-(2-(7-Methoxy-3,4-dihydroisoquinolin-1-yl)ethyl)-3-(4-(trifluoromethyl)phenyl)urea oxalate (6b-3)

Following general procedure to obtain pure product as pale yellow oil 238 mg (yield: 334.6%). ^1^H-NMR (DMSO-*d*_*6*_): *δ* 9.11 (s, 1H, -NH-), 8.59 (d, 1H, *J* = 4.4 Hz, -NH-), 7.66 (s, 3H, Ar-H), 7.51 (s, 1H, Ar-H), 7.34 (d, 1H, *J* = 8.4 Hz, Ar-H), 7.17 (d, 2H, *J* = 5.6 Hz, Ar-H), 7.06 (t, 1H, *J* = 8.0 Hz, Ar-H), 4.09 (t, 2H, *J* = 5.6 Hz, -CH_2_-), 3.84 (s, 3H, -OCH_3_), 3.80–3.77 (m, 2H, -CH_2_-), 3.72 (s, 2H, -CH_2_-), 2.97 (d, 2H, *J* = 8.4 Hz, -CH_2_-); ^13^C-NMR (DMSO-*d*_*6*_): *δ*163.7, 161.6, 159.9, 158.6, 133.9, 130.2, 129.4, 128.8, 127.4, 119.2, 119.1, 111.2, 99.9, 99.5, 55.6, 40.5, 37.6, 25.5; HRMS (TOF) *m/z* calcd. for C_20_H_20_F_3_N_3_O_2_ [M+H^+^]: 392.1586, found: 392.1497.

#### 1-(2-(7-Methoxy-3,4-dihydroisoquinolin-1-yl)ethyl)-3-(2-(trifluoromethyl)phenyl)urea oxalate (6b-4)

Following general procedure to obtain pure product as pale yellow oil 189 mg (yield: 33.8%). ^1^H-NMR (DMSO-*d*_*6*_): *δ* 9.11 (s, 1H, -NH-), 8.59 (d, 1H, *J* = 4.8 Hz, -NH-), 7.51 (s, 1H, Ar-H), 7.37 (t, 2H, *J* = 7.6 Hz, Ar-H), 7.23 (d, 1H, *J* = 8.4 Hz, Ar-H), 7.05 (t, 1H, *J* = 8.0 Hz, Ar-H), 6.88 (dd, 2H, *J*_1_ = 12.8 Hz, *J*_2_ = 2.4 Hz, Ar-H), 4.08 (t, 2H, *J* = 5.6 Hz, -CH_2_-), 3.84 (s, 3H, -OCH_3_), 3.72 (d, 2H, *J* = 4.8 Hz, -CH_2_-), 2.98–2.89 (m, 2H,-CH_2_-), 2.72–2.59 (m, 2H, -CH_2_-); ^13^C-NMR (DMSO-*d*_*6*_): *δ*164.0, 161.2, 159.9, 158.7, 134.6, 130.3, 129.6, 128.8, 119.3, 113.0, 99.9, 99.5, 55.7, 46.2, 35.7, 28.8, 25.5; HRMS (TOF) *m/z* calcd. for C_16_H_21_N_3_O_2_ [M+H^+^]: 288.1712, found: 288.1695.

#### 1-(2-(7-Methoxy-3,4-dihydroisoquinolin-1-yl)ethyl)-3-(3,4,5-trichlorophenyl)urea oxalate(6b-5)

Following general procedure to obtain pure product as white solid 143 mg (yield: 23.3%). mp: >220 °C; ^1^H-NMR (DMSO-*d*_*6*_): *δ* 8.86 (s, 1H, -NH-), 8.57 (d, 1H, *J* = 4.8 Hz, -NH-), 7.82 (s, 2H, Ar-H), 7.49 (d, 1H, *J* = 4.8 Hz, Ar-H), 7.34 (d, 1H, *J* = 8.0 Hz, Ar-H), 7.16 (d, 1H, *J* = 5.6 Hz, Ar-H), 4.09 (t, 2H, *J* = 4.8 Hz, -CH_2_-), 4.02–3.96 (m, 1H, -CH_2_-), 3.83 (s, 3H, -OCH_3_), 3.75 (t, 2H, *J* = 8.4 Hz, -CH_2_-), 2.96 (t, 2H, *J* = 8.0 Hz, -CH_2_-), 2.08 (s, 1H, -CH_2_-); ^13^C-NMR (DMSO-*d*_*6*_): *δ*164.4, 161.2, 158.3, 155.4, 134.4, 130.2, 129.4, 128.7, 127.5, 127.0, 119.7, 116.7, 55.5, 46.2, 36.7, 31.6, 25.6; HRMS (TOF) *m/z* calcd. for C_19_H_18_Cl_3_N_3_O_2_ [M+H^+^]: 426.0543, found: 426.0579.

#### 1-(4-Cyano-2,6-dimethylphenyl)-3-(2-(7-methoxy-3,4-dihydroisoquinolin-1-yl)ethyl)urea oxalate(6b-6)

Following general procedure to obtain pure product as white solid 217 mg (yield: 40.3%). ^1^H-NMR (DMSO-*d*_*6*_): *δ* 8.56 (d, 1H, *J* = 4.8 Hz, -NH-), 8.19 (s, 1H,-NH-), 7.49 (s, 1H, Ar-H), 7.32 (d, 1H, *J* = 8.4 Hz, Ar-H), 7.27 (s, 2H, Ar-H), 7.13 (dd, 1H, *J*_1_ = 12.8 Hz, *J*_2_ = 2.4 Hz, Ar-H), 4.07 (t, 2H, *J* = 8.0 Hz, -CH_2_-), 4.03–3.95 (m, 1H, -CH_2_-), 3.83 (s, 3H, -OCH_3_), 3.80–3.71 (m, 2H, -CH_2_-), 3.17 (s, 1H,-CH_2_-), 2.96 (t, 2H, *J* = 7.6 Hz, -CH_2_-), 2.13 (s, 6H,-CH_3_); ^13^C-NMR (DMSO-*d*_*6*_): *δ*164.5, 161.2, 158.5, 155.8, 138.9, 134.8, 130.6, 130.1, 128.5, 127.5, 118.8, 118.2, 113.5, 99.8, 55.5, 48.5, 37.7, 35.8, 25.6, 16.7; HRMS (TOF) *m/z* calcd. for C_22_H_24_N_4_O_2_ [M+H^+^]: 377.1978, found: 377.1879.

#### 1-(4-Cyano-3-fluorophenyl)-3-(2-(7-methoxy-3,4-dihydroisoquinolin-1-yl)ethyl)urea oxalate (6b-7)

Following general procedure to obtain pure product as white solid 162 mg (yield: 30.9%). mp: 207–209 °C; ^1^H-NMR (DMSO-*d*_*6*_): *δ* 9.10 (s, 1H,-NH-), 8.59 (d, 1H, *J* = 4.8 Hz, -NH-), 7.67 (s, 2H, Ar-H), 7.51 (d, 1H, *J* = 5.6 Hz, Ar-H), 7.36 (d, 1H, *J* = 8.0 Hz, Ar-H), 7.16 (dd, 2H, *J*_1_ = 12.8 Hz, *J*_2_ = 2.4 Hz, Ar-H), 4.08 (t, 2H, *J* = 8.0 Hz, -CH_2_-), 3.83 (s, 3H, -OCH_3_), 3.73 (t, 2H, *J* = 4.8 Hz, -CH_2_-), 2.97 (t, 2H, *J* = 8.4 Hz, -CH_2_-), 2.73 (t, 2H, *J* = 7.6 Hz, -CH_2_-); ^13^C-NMR (DMSO-*d*_*6*_): *δ*164.5, 161.2, 160.8, 158.3, 155.6, 141.0, 134.8, 130.6, 129.9, 121.3, 118.9, 113.5, 110.2, 108.5, 55.6, 48.6, 37.7, 35.8, 25.6; HRMS (TOF) *m/z* calcd. for C_20_H_19_FN_4_O_2_ [M+H^+^]: 367.1570, found: 367.1614.

#### 2-(3-(2-(7-Methoxy-3,4-dihydroisoquinolin-1-yl)ethyl)ureido)thiazole-5-carboxylate oxalate(6b-8)

Following general procedure to obtain pure product as white solid 72 mg (yield: 12.5%). mp: 125–127 °C; ^1^H-NMR (DMSO-*d*_*6*_): *δ* 9.08 (s, 1H,-NH-), 8.59 (d, 1H, *J* = 4.8 Hz, -NH-), 7.51 (d, 1H, *J* = 5.2 Hz, Ar-H), 7.35 (d, 1H, *J* = 8.4 Hz, Ar-H), 7.15 (t, 2H, *J* = 8.0 Hz, Ar-H), 4.32 (dd, 2H, *J*_1_ = 12.8 Hz, *J*_2_ = 2.4 Hz, -CH_2_-), 4.08 (t, 2H, *J* = 8.4 Hz, -CH_2_-), 3.83 (s, 3H, -OCH_3_), 3.80–3.72 (m, 2H, -CH_2_-), 2.97 (t, 2H, *J* = 8.0 Hz, -CH_2_-), 2.91 (t, 1H, *J* = 4.8 Hz, -CH_2_-), 2.72–2.59 (m, 1H,-CH_2_-), 1.27 (s, 3H, -CH_3_); ^13^C-NMR (DMSO-*d*_*6*_): *δ*164.1, 162.7, 161.7, 160.9, 158.6, 155.5, 152.9, 134.1, 129.4, 128.8, 119.2, 111.1, 60.3, 55.6, 40.5, 37.7, 35.3, 25.6, 14.8; HRMS (TOF) *m/z* calcd. for C_19_H_22_N_4_O_4_S [M+H^+^]: 403.1440, found: 402.8830.

#### 1-(4-Chloro-3-(trifluoromethyl)phenyl)-3-(2-(7-methoxy-3,4-dihydroisoquinolin-1-yl)ethyl)urea oxalate (6b-9)

Following general procedure to obtain pure product as colorless oil 296 mg (yield: 40.2%). ^1^H-NMR (DMSO-*d*_*6*_): *δ* 9.08 (s, 1H,-NH-), 8.59 (d, 1H, *J* = 4.8 Hz, -NH-), 7.89 (t, 1H, *J* = 5.6 Hz, Ar-H), 7.76–7.72 (m, 2H, Ar-H), 7.51 (d, 1H, *J* = 8.0 Hz, Ar-H), 7.33 (d, 1H, *J* = 8.4 Hz, Ar-H), 7.12 (dd, 1H, *J*_1_ = 12.8 Hz, *J*_2_ = 2.4 Hz, Ar-H), 4.08 (t, 2H, *J* = 8.0 Hz, -CH_2_-), 3.83 (s, 3H, -OCH_3_), 3.72 (t, 2H, *J* = 5.6 Hz, -CH_2_-), 2.97 (t, 2H, *J* = 7.2 Hz, -CH_2_-), 2.71 (t, 2H, *J* = 8.0 Hz, -CH_2_-); ^13^C-NMR (DMSO-*d*_*6*_): *δ*164.5, 161.2, 158.3, 155.6, 141.0, 134.8, 130.6, 129.9, 128.8, 121.3, 121.4, 118.9, 113.5, 110.2, 108.5, 55.6, 48.6, 37.7, 35.8, 25.8; HRMS (TOF) *m/z* calcd. for C_20_H_19_FN_4_O_2_ [M+H^+^]: 367.1570, found: 367.1614.

#### 1-Isopropyl-3-(2-(7-methoxy-3,4-dihydroisoquinolin-1-yl)ethyl)urea oxalate(6b-10)

Following general procedure to obtain pure product as pale yellow solid powder 143 mg (yield: 26.3%). mp: 157–159 °C; ^1^H-NMR (DMSO- *d*_*6*_): *δ* 8.86 (s, 1H,-NH-), 8.58 (d, 1H, *J* = 8.0 Hz, -NH-), 7.50 (s, 1H, Ar-H), 7.32 (t, 1H, *J* = 8.4 Hz, Ar-H), 7.17–7.11 (m, 1H, Ar-H), 4.08 (t, 2H, *J* = 7.6 Hz, -CH_2_-), 4.04–3.97 (m, 1H, -CH_2_-), 3.84 (s, 3H, -OCH_3_), 3.81–3.74 (t, 2H, *J* = 5.6 Hz, -CH_2_-), 2.97 (t, 2H, *J* = 8.4 Hz, -CH_2_-), 2.89 (t, 1H, *J* = 7.6 Hz, -CH_2_-), 2.09 (m, 1H, -CH-), 1.44 (d, 6H, *J* = 12.8 Hz, -CH_3_); ^13^C-NMR (DMSO-*d*_*6*_): *δ*164.5, 163.1, 158.6, 155.8, 152.5, 134.4, 129.4, 128.7, 119.6, 118.9, 111.0, 99.7, 55.6, 40.4, 30.7, 25.6; HRMS (TOF) *m/z* calcd. for C_16_H_23_N_3_O_2_ [M+H^+^]: 290.1869, found: 290.9096.

#### 1-(3,5-Dimethylpyridin-4-yl)-3-(2-(7-methoxy-3,4-dihydroisoquinolin-1-yl)ethyl)urea oxalate (6b-11)

Following general procedure to obtain pure product as pale yellow oil 146 mg (yield: 21.1%). ^1^H-NMR (DMSO-*d*_*6*_): *δ* 9.09 (s, 1H,-NH-), 8.58 (d, 1H, *J* = 5.6 Hz, -NH-), 7.51 (s, 1H, Ar-H), 7.33 (d, 1H, *J* = 8.4 Hz, Ar-H), 7.16 (d, 2H, Ar-H), 7.07–7.00 (m, 1H, Ar-H), 4.08 (t, 2H, *J* = 8.0 Hz, -CH_2_-), 3.83 (s, 3H, -OCH_3_), 3.77 (t, 2H, *J* = 5.6 Hz, -CH_2_-), 2.97 (t, 2H, *J* = 7.2 Hz, -CH_2_-), 2.91 (t, 2H, *J* = 8.4 Hz, -CH_2_-); ^13^C-NMR (DMSO-*d*_*6*_): *δ*164.6, 163.1, 158.3, 155.8, 154.0, 147.5, 134.4, 130.5, 128.8, 119.6, 113.2, 99.7, 55.6, 37.7, 35.8, 30.7, 25.7; HRMS (TOF) *m/z* calcd. for C_18_H_19_Cl_2_N_4_O_2_ [M+H^+^]: 393.0885, found: 393.0774.

#### 1-(5-Bromopyridin-2-yl)-3-(2-(7-methoxy-3,4-dihydroisoquinolin-1-yl)ethyl)urea oxalate(6b-12)

Following general procedure to obtain pure product as white solid 151 mg (yield: 21.4%). mp: 186–188 °C; ^1^H-NMR (DMSO-*d*_*6*_): *δ* 8.79 (s, 1H,-NH-), 8.58 (d, 1H, *J* = 4.4 Hz, -NH-), 7.51 (s, 1H, Ar-H), 7.33 (dd, 2H, *J*_1_ = 15.6 Hz, *J*_2_ = 4.8 Hz, Ar-H), 7.16 (dd, 2H, *J*_1_ = 8.4 Hz, *J*_2_ = 2.0 Hz, Ar-H), 7.12 (d, 1H, *J* = 8.0 Hz, Ar-H), 4.04 (t, 2H, *J* = 8.0 Hz, -CH_2_-), 3.83 (s, 3H, -OCH_3_), 3.78 (t, 2H, *J* = 7.2 Hz, -CH_2_-), 2.97 (t, 2H, *J* = 7.6 Hz, -CH_2_-), 2.91 (d, 2H, *J* = 5.6 Hz, -CH_2_-); ^13^C-NMR (DMSO-*d*_*6*_): *δ*164.6, 163.1, 157.5, 155.8, 152.7, 147.5, 130.6, 128.2, 127.8, 119.6, 113.2, 111.3, 104.8, 99.7, 55.6, 46.7, 37.7, 35.8, 25.7; HRMS (TOF) *m/z* calcd. for C_18_H_19_BrN_4_O_2_ [M+H^+^]: 403.0770, found: 403.0736.

#### 1-(2-(7-Methoxy-3,4-dihydroisoquinolin-1-yl)ethyl)-3-(naphthalen-1-yl)urea oxalate(6b-13)

Following general procedure to obtain pure product as brown oil 78 mg (yield: 11.8%). ^1^H-NMR (DMSO-*d*_*6*_): *δ* 9.03 (s, 1H,-NH-), 8.59 (d, 1H, *J* = 8.4 Hz, -NH-), 7.66 (s, 1H, Ar-H), 7.55 (s, 2H, Ar-H), 7.51 (s, 1H, Ar-H), 7.43 (d, 3H, *J* = 8.0 Hz, Ar-H), 7.34 (d, 2H, *J* = 7.6 Hz, Ar-H), 7.14 (t, 1H, *J* = 8.0 Hz, Ar-H), 4.09 (t, 1H, *J* = 7.2 Hz, -CH_2_-), 3.86 (s, 3H, -OCH_3_), 3.84 (s, 1H, -CH_2_-), 3.78 (t, 2H, *J* = 8.4 Hz, -CH_2_-), 3.47 (s, 2H, *J* = 8.0 Hz, -CH_2_-), 2.91 (d, 2H, *J* = 4.8 Hz, -CH_2_-); ^13^C-NMR (DMSO-*d*_*6*_): *δ*164.3, 162.7, 157.6, 154.7, 140.6, 134.9, 130.3, 128.2, 127.8, 126.4, 125.0, 124.8, 121.8, 119.6, 113.4, 105.9, 55.6, 46.7, 37.7, 35.8, 25.9; HRMS (TOF) *m/z* calcd. for C_23_H_23_N_3_O_2_ [M+H^+^]: 374.1869, found: 374.2023.

#### 1-(3-Chloro-4-fluorophenyl)-3-(2-(7-methoxy-3,4-dihydroisoquinolin-1-yl)ethyl)urea oxalate (6b-14)

Following general procedure to obtain pure product as pale yellow solid 231 mg (yield: 34.7%). ^1^H-NMR (DMSO-*d*_*6*_): *δ* 9.08 (s, 1H,-NH-), 8.59 (d, 1H, *J* = 4.8 Hz, -NH-), 7.86 (t, 1H, *J* = 5.6 Hz, Ar-H), 7.76–7.72 (m, 2H, *J* = 4.8 Hz, Ar-H), 7.51 (d, 1H, *J* = 4.8 Hz, Ar-H), 7.42 (d, 1H, *J* = 8.4 Hz, Ar-H), 7.13 (dd, 1H, *J*_1_ = 8.4 Hz, *J*_2_ = 2.4 Hz, Ar-H), 4.08 (t, 2H, *J* = 8.4 Hz, -CH_2_-), 3.83 (s, 3H, -OCH_3_), 3.72 (t, 2H, *J* = 5.6 Hz, -CH_2_-), 2.98 (t, 2H, *J* = 8.4 Hz, -CH_2_-), 2.73 (t, 2H, *J* = 7.6 Hz, -CH_2_-); ^13^C-NMR (DMSO-*d*_*6*_): *δ*164.2, 161.4, 158.0, 155.0, 134.4, 132.2, 130.2, 129.5, 128.8, 124.3, 123.5, 121.4, 118.9, 113.7, 55.6, 46.7, 37.7, 35.6, 25.8; HRMS (TOF) *m/z* calcd. for C_19_H_19_ClFN_3_O_2_ [M+H^+^]: 376.1228, found: 376.2065.

#### 1-(2-(7-Methoxy-3,4-dihydroisoquinolin-1-yl)ethyl)-3-(3-nitrophenyl)urea oxalate(6b-15)

Following general procedure to obtain pure product as yellow oil 126 mg (yield: 19.2%). ^1^H-NMR (DMSO-*d*_*6*_): *δ* 8.93 (s, 1H,-NH-), 8.59 (d, 1H, *J* = 4.8 Hz, -NH-), 7.61 (s, 2H, Ar-H), 7.50 (s, 1H, Ar-H), 7.35 (d, 1H, *J* = 5.6 Hz, Ar-H), 7.30–7.22 (m, 1H, Ar-H), 7.17–7.11 (m, 2H, Ar-H), 4.08 (t, 2H, *J* = 8.4 Hz, -CH_2_-), 3.83 (s, 3H, -OCH_3_), 3.72 (t, 2H, *J* = 4.8 Hz, -CH_2_-), 2.97 (t, 2H, *J* = 8.0 Hz, -CH_2_-), 2.71 (t, 2H, *J* = 7.2 Hz, -CH_2_-); ^13^C-NMR (DMSO-*d*_*6*_): *δ*164.2, 161.2, 157.9, 148.9, 136.5, 130.3, 129.5, 129.1, 128.8, 126.6, 123.5, 119.3, 118.8, 113.4, 113.0, 55.6, 46.7, 37.7, 35.5, 25.8; HRMS (TOF) *m/z* calcd. for C_19_H_20_N_4_O_4_ [M+H^+^]: 369.1263, found: 369.1347.

#### 1-(4-Chloro-2-nitrophenyl)-3-(2-(7-methoxy-3,4-dihydroisoquinolin-1-yl)ethyl)urea oxalate (6b-16)

Following general procedure to obtain pure product as white solid 212 mg (yield: 30.1%). mp: 128–130 °C; ^1^H-NMR (DMSO-*d*_*6*_): *δ* 8.93 (s, 1H,-NH-), 8.59 (d, 1H, *J* = 5.6 Hz, -NH-), 7.50 (d, 1H, *J* = 4.8 Hz, Ar-H), 7.35 (d, 1H, *J* = 8.4 Hz, Ar-H), 7.17–7.11 (m, 2H, Ar-H), 7.05 (d, 1H, *J* = 8.0 Hz, Ar-H), 6.84 (s, 1H, Ar-H), 4.08 (t, 2H, *J* = 8.4 Hz, -CH_2_-), 3.83 (s, 3H, -OCH_3_), 3.72 (t, 2H, *J* = 5.6 Hz, -CH_2_-), 2.97 (t, 2H, *J* = 8.4 Hz, -CH_2_-), 2.73 (t, 2H, *J* = 8.4 Hz, -CH_2_-); ^13^C-NMR (DMSO-*d*_*6*_): *δ*164.2, 161.3, 157.7, 143.7, 135.5, 133.3, 130.2, 129.4, 129.2, 128.8, 126.7, 123.8, 119.7, 117.4, 113.3, 55.6, 46.8, 38.0, 35.2, 25.6; HRMS (TOF) *m/z* calcd. for C_19_H_19_ClN_4_O_4_ [M+H^+^]: 403.1173, found: 402.8860.

#### 1-(2-(7-Methoxy-3,4-dihydroisoquinolin-1-yl)ethyl)-3-(thiazol-2-yl)urea oxalate(6b-17)

Following general procedure to obtain pure product as white solid 109 mg (yield: 18.1%). mp: 160–162 °C; ^1^H-NMR (DMSO-*d*_*6*_): *δ* 8.64 (s, 1H,-NH-), 8.58 (d, 1H, *J* = 7.6 Hz, -NH-), 7.49 (d, 2H, *J* = 5.2 Hz, Ar-H), 7.34 (dd, 1H, *J*_1_ = 12.8 Hz, *J*_2_ = 2.4 Hz, Ar-H), 7.16–7.11 (m, 2H, Ar-H), 4.08 (t, 2H, *J* = 8.0 Hz, -CH_2_-), 3.83 (s, 3H, -OCH_3_), 3.69 (t, 2H, *J* = 5.6 Hz, -CH_2_-), 2.99 (t, 2H, *J* = 8.4 Hz, -CH_2_-), 2.71 (t, 2H, *J* = 7.6 Hz, -CH_2_-); ^13^C-NMR (DMSO-*d*_*6*_): *δ*164.2, 163.1, 161.3, 157.8, 154.3, 132.7, 130.2, 129.3, 128.8, 119.5, 113.7, 112.4, 55.6, 46.8, 37.9, 35.4, 25.7; HRMS (TOF) *m/z* calcd. for C_16_H_18_ClN_4_O_2_S [M+H^+^]: 331.1229, found: 331.1248.

#### 1-(3,4-Dichlorophenyl)-3-(2-(7-methoxy-3,4-dihydroisoquinolin-1-yl)ethyl)urea oxalate(6b-18)

Following general procedure to obtain pure product as yellow solid 163 mg (yield: 23.6%). mp: 134–136 °C; ^1^H-NMR (DMSO-*d*_*6*_): *δ* 8.64 (s, 1H,-NH-), 8.58 (d, 1H, *J* = 8.0 Hz, -NH-), 7.49 (d, 2H, *J* = 4.8 Hz, Ar-H), 7.49 (d, 1H, *J* = 8.4 Hz, Ar-H), 7.35 (d, 2H, *J* = 8.4 Hz, Ar-H), 7.17–7.11 (m, 3H, Ar-H), 4.08 (t, 2H, *J* = 7.6 Hz, -CH_2_-), 3.83 (s, 3H, -OCH_3_), 3.69 (t, 2H, *J* = 5.6 Hz, -CH_2_-), 2.97 (t, 2H, *J* = 7.2 Hz, -CH_2_-), 2.73 (t, 2H, *J* = 7.2 Hz, -CH_2_-); ^13^C-NMR (DMSO-*d*_*6*_): *δ*164.4, 163.3, 158.6, 155.8, 152.5, 134.4, 129.4, 128.7, 127.5, 119.6, 118.9, 111.0, 99.8, 55.5, 40.4, 30.7, 25.6; HRMS (TOF) *m/z* calcd. for C_19_H_19_Cl_2_N_3_O_2_ [M+H^+^]: 392.0933, found: 392.1105.

#### 1-(2-(7-Methoxy-3,4-dihydroisoquinolin-1-yl)ethyl)-3-(2-nitrophenyl)ureaoxalate(6b-19)

Following general procedure to obtain pure product as yellow oil 236 mg (yield: 34.9%). ^1^H-NMR (DMSO-*d*_*6*_): *δ* 8.93 (s, 1H,-NH-), 8.59 (d, 1H, *J* = 4.8 Hz, -NH-), 7.61 (s, 2H, Ar-H), 7.40 (s, 1H, Ar-H), 7.35 (d, 2H, *J* = 5.6 Hz, Ar-H), 7.23 (d, 2H, *J* = 4.8 Hz, Ar-H), 4.09 (t, 2H, *J* = 8.4 Hz, -CH_2_-), 3.83 (s, 3H, -OCH_3_), 3.73 (t, 2H, *J* = 5.6 Hz, -CH_2_-), 2.97 (t, 2H, *J* = 8.0 Hz, -CH_2_-), 2.73 (t, 2H, *J* = 8.4 Hz, -CH_2_-); ^13^C-NMR (DMSO-*d*_*6*_): *δ* 164.2, 162.5, 158.6, 155.4, 143.2, 131.3, 129.7, 129.2, 128.9, 127.2, 119.8, 118.7, 113.4, 55.6, 47.0, 37.2, 34.2, 26.0; HRMS (TOF) *m/z* calcd. for C_19_H_20_N_4_O_4_ [M+H^+^]: 369.1263, found: 369.1198.

#### 1-(4-(Cyanomethyl)phenyl)-3-(2-(7-methoxy-3,4-dihydroisoquinolin-1-yl)ethyl)urea oxalate(6b-20)

Following general procedure to obtain pure product as white solid 157 mg (yield: 24.3%). mp: 141–143 °C; ^1^H-NMR (DMSO-*d*_*6*_): *δ* 9.11 (s, 1H, -NH-), 8.59 (d, 1H, *J* = 8.4 Hz, -NH-), 7.63 (s, 2H, Ar-H), 7.48 (s, 1H, Ar-H), 7.31 (d, 1H, *J* = 8.0 Hz, Ar-H), 7.15 (d, 2H, *J* = 4.8 Hz, Ar-H), 7.06 (t, 1H, *J* = 7.2 Hz, Ar-H), 4.32 (m, 2H, -CH_2_-), 4.09 (t, 2H, *J* = 8.4 Hz, -CH_2_-), 3.84 (s, 3H, -OCH_3_), 3.77–3.72 (m, 2H, -CH_2_-), 2.97 (d, 2H, *J* = 5.6 Hz, -CH_2_-), 2.73 (t, 2H, *J* = 4.8 Hz, -CH_2_-); ^13^C-NMR (DMSO-*d*_*6*_): *δ* 174.6, 164.1, 161.5, 158.6, 155.5, 152.8, 134.1, 128.8, 119.2, 119.0, 111.1, 99.8, 55.6, 46.3, 37.6, 25.5; HRMS (TOF) *m/z* calcd. for C_21_H_22_N_4_O_2_ [M+H^+^]: 363.1821, found: 363.1228.

#### 1-(4-Bromophenyl)-3-(2-(7-methoxy-3,4-dihydroisoquinolin-1-yl)ethyl)ureaoxalate(6b-21)

Following general procedure to obtain pure product as yellow oil 162 mg (yield: 23.6%). ^1^H-NMR (DMSO-*d*_*6*_): *δ* 9.10 (s, 1H, -NH-), 8.59 (d, 1H, *J* = 4.8 Hz, -NH-), 7.67 (s, 3H, Ar-H), 7.50 (s, 1H, Ar-H), 7.35 (d, 1H, *J* = 8.4 Hz, Ar-H), 7.15 (dd, 2H, *J*_1_ = 12.8 Hz, *J*_2_ = 2.4 Hz, Ar-H), 4.32 (m, 2H, -CH_2_-), 4.08 (t, 2H, *J* = 5.6 Hz, -CH_2_-), 3.84 (s, 3H, -OCH_3_), 3.73 (t, 2H, *J* = 5.6 Hz, -CH_2_-), 2.97 (d, 2H, *J* = 8.4 Hz, -CH_2_-), 2.71 (t, 2H, *J* = 7.2 Hz, -CH_2_-); ^13^C-NMR (DMSO-*d*_*6*_): *δ*164.3, 161.6, 158.6, 155.6, 138.6, 130.8, 129.4, 127.5, 122.8, 122.0, 119.5, 113.1, 55.6, 43.3, 39.9, 25.6; HRMS (TOF) *m/z* calcd. for C_19_H_20_BrN_3_O_2_ [M+H^+^]: 402.0817, found: 402.0762.

#### 1-(3-Bromopyridin-2-yl)-3-(2-(7-methoxy-3,4-dihydroisoquinolin-1-yl)ethyl)urea oxalate (6b-22)

Following general procedure to obtain pure product as white solid 267 mg (yield: 37.9%). mp: 131–134 °C; ^1^H-NMR (DMSO-*d*_*6*_): *δ* 9.11 (s, 1H, -NH-), 8.58 (d, 1H, *J* = 4.8 Hz, -NH-), 7.68 (s, 2H, Ar-H), 7.51 (s, 1H, Ar-H), 7.33 (dd, 2H, *J*_1_ = 15.6 Hz, *J*_2_ = 3.2 Hz, Ar-H), 7.17–7.14 (m, 2H, Ar-H), 4.04 (t, 2H, *J* = 8.4 Hz, -CH_2_-), 3.83 (s, 3H, -OCH_3_), 3.78 (t, 2H, *J* = 8.0 Hz, -CH_2_-), 2.99 (t, 2H, *J* = 7.6 Hz, -CH_2_-), 2.91 (d, 2H, *J* = 5.6 Hz, -CH_2_-); ^13^C-NMR (DMSO-*d*_*6*_): *δ*164.1, 163.2, 161.64, 158.6, 155.5, 152.8, 134.1, 129.4, 128.8, 127.5, 123.3, 119.2, 110.1, 99.8, 55.6, 40.5, 37.7, 35.8, 25.6; HRMS (TOF) *m/z* calcd. for C_18_H_19_BrN_4_O_2_ [M+H^+^]: 403.0770, found: 403.0745.

## Additional Information

**How to cite this article**: Yang, Y. *et al.* Scaffold Hopping Toward Agomelatine: Novel 3, 4-Dihydroisoquinoline Compounds as Potential Antidepressant Agents. *Sci. Rep.* **6**, 34711; doi: 10.1038/srep34711 (2016).

## Supplementary Material

Supplementary Information

## Figures and Tables

**Table 1:** The protection rates of the target compounds on corticosterone-injured PC12 cells.

Compd.	PR^a^ (%)		
	1.25 μM	2.5 μM	5 μM
**6a-1**	25.4	37.1	42.1
**6a-2**	32.7	18.1	3.1
**6a-9**	20.3	14.9	7.9
**Ago.**	28.1	24.2	17.6

^a^PR represents the protection rate of the tested compounds measured at 24 h after treatment with different concentrations of compounds. PR = (A_d_ − A_c_)/A_c_ * 100%.

**Table 2:** Prediction of blood-brain barrier penetration of drugs expressed as Pe ± SD (n = 3).

Drugs	Lit.^a^	Pe (*10^−6^ cm/s)	CNS(+/−)
Verapamil	16	16.90 ± 0.36	CNS+
Oxazepam	10	9.60 ± 0.21	CNS+
Diazepam	16	11.86 ± 0.23	CNS+
Clonidine	5.3	5.10 ± 0.16	CNS+
Imipramine	13	10.10 ± 0.22	CNS+
Testosterone	17	16.30 ± 0.25	CNS+
Caffeine	1.3	1.28 ± 0.05	CNS−
Enoxacine	0.9	0.47 ± 0.01	CNS−
Piroxicam	2.5	0.72 ± 0.02	CNS−
Norfloxacin	0.1	0.42 ± 0.01S	CNS−
Theophylline	0.12	0.10 ± 0.003	CNS−
Agomelatine	—	17.79 ± 0.37	CNS+
**6a-1**	—	16.67 ± 0.41	CNS+

^a^The Pe values were recorded in literature^38^.
