# Supplementary material for: Scaffold Hopping Toward Agomelatine: Novel 3, 4-Dihydroisoquinoline Compounds as Potential Antidepressant Agents
Source: Sci Rep. 2016 Oct 4;6:34711. doi: 10.1038/srep34711 (PMC5048153; doi:10.1038/srep34711)

**Supplementary information**

**Scaffold Hopping Toward Agomelatine: Novel 3, 4-Dihydroisoquinoline Compounds as Potential Antidepressant Agents**

Yang Yang^a, *^, Wei Ang^a,b,*,#^, Haiyue Long^a,*^, Ying Chang^a^, Zicheng Li^c^, Liangxue Zhou^a^, Tao Yang^a^, Yong Deng^a,b^ ,Youfu Luo^a^

^a^State Key Laboratory of Biotherapy and Department of Neurosurgery/Collaborative Innovation Center for Biotherapy, West China Hospital, West China Medical School, Sichuan University, Chengdu, Sichuan 610041, PR China

^b^Key Laboratory of Drug Targeting and Drug Delivery System, Ministry of Education, West China School of Pharmacy, Sichuan University, Chengdu, Sichuan 610041, PR China

^c^Department of Pharmaceutical and Bioengineering, School of Chemical Engineering, Sichuan University, Chengdu, Sichuan 610065, PR China

^#^Current address: Department of Pharmacy, The Third Affiliated Hospital, Anhui Medical University, The First People’s Hospital of Hefei, Hefei, Anhui, 230061, PR China

**Contents**

**Figure S1** Four pharmacophores produced by Common Feature Pharmacophore Generation protocol in DS2.55

**Figure S2** The fitting values vs. names of profiled compounds

**Figure S3** Chemical structures of alkaloid compounds for the scaffold extraction.

**Figure S4** The effect of compound **6a-1**on the mRNA levels of NGF in corticosterone injured PC12 cells.

**Figure S5** Hoechst 33258 staining of Compound **6a-1** regulate the apoptosis of corticosterone injured PC12 cells.

**Figure S6** Compound **6a-1** regulates the intracellular Calcium ion concentration of corticosterone-injured PC12 cells.

**Figure S7** The effect of compound **6a-1** on the VEGF and IGF-1 level *in vivo*.

**Figure S8** Immunohistochemistry examination of the effect on cell survival, proliferation and maturation in rat hippocampus induced by compound **6a-1**.

**Table S1** The protection rates of 3,4-dihydroisoquinoline compounds on corticosterone-injured PC12 cells.

**Table S2** Cytotoxicities of 3, 4-dihydroisoquinoline compounds on human embryonic kidney 293 cells and human normal liver L02 cells.

**Table S3** Calculated logP (ClogP) and topological polar surface area (tPSA) values of the target compounds.

**Table S4** IC_50_values of the compound **6a-1** on H9C2 cells and hERG K^+^channel.

**Table S5** FLIPR assays of compound **6a-1** on 5-HT_2C_ receptor antagonism effects.

**
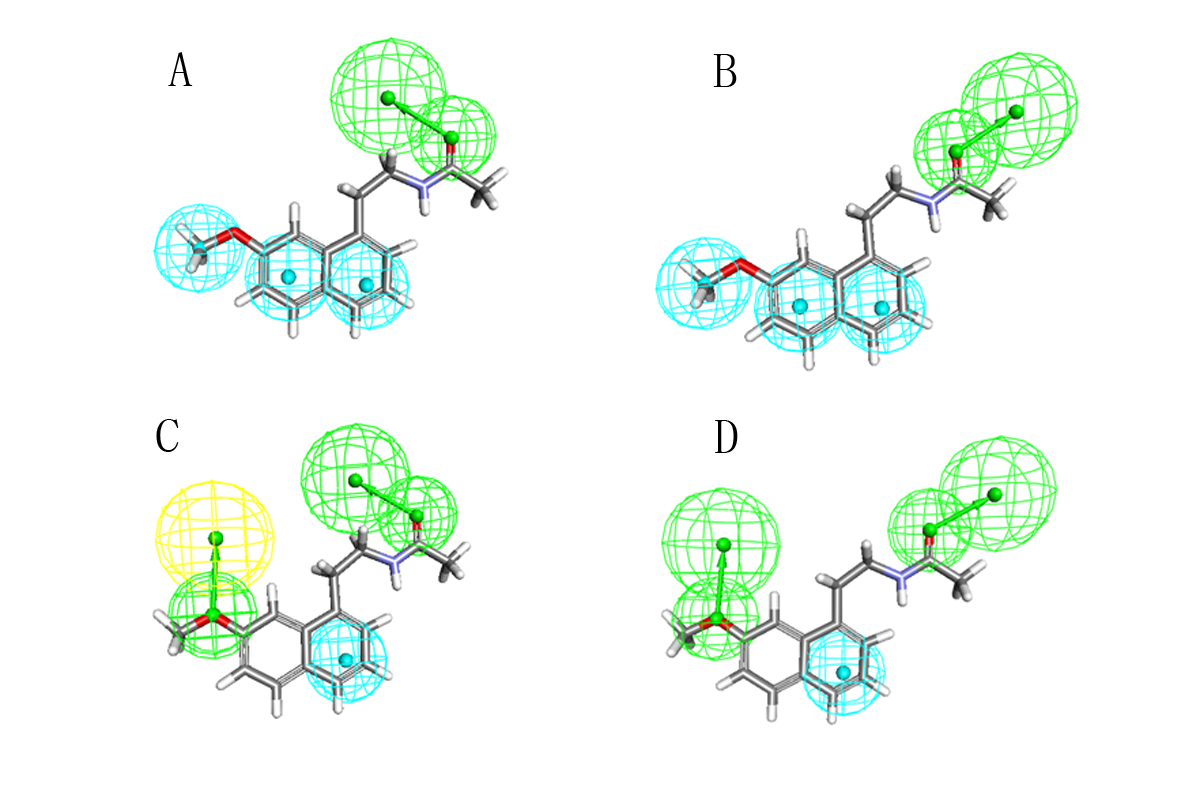
**

**Figure S1 Four pharmacophores produced by Common Feature Pharmacophore Generation protocol in DS2.55**

(A) Agomelatine_01; (B) Agomelatine_02; (C) Agomelatine_03; (D) Agomelatine_04

**
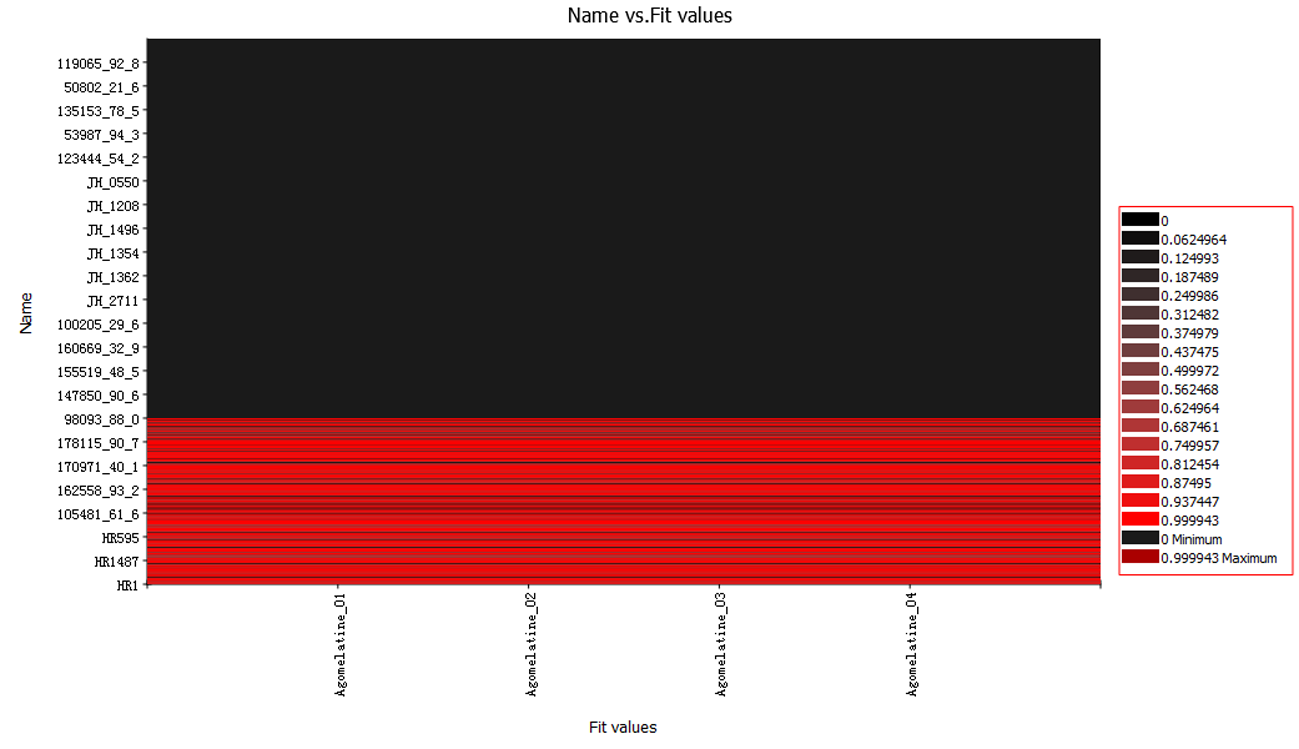
**

**Figure S2 The fitting values vs. names of profiled compounds**

**
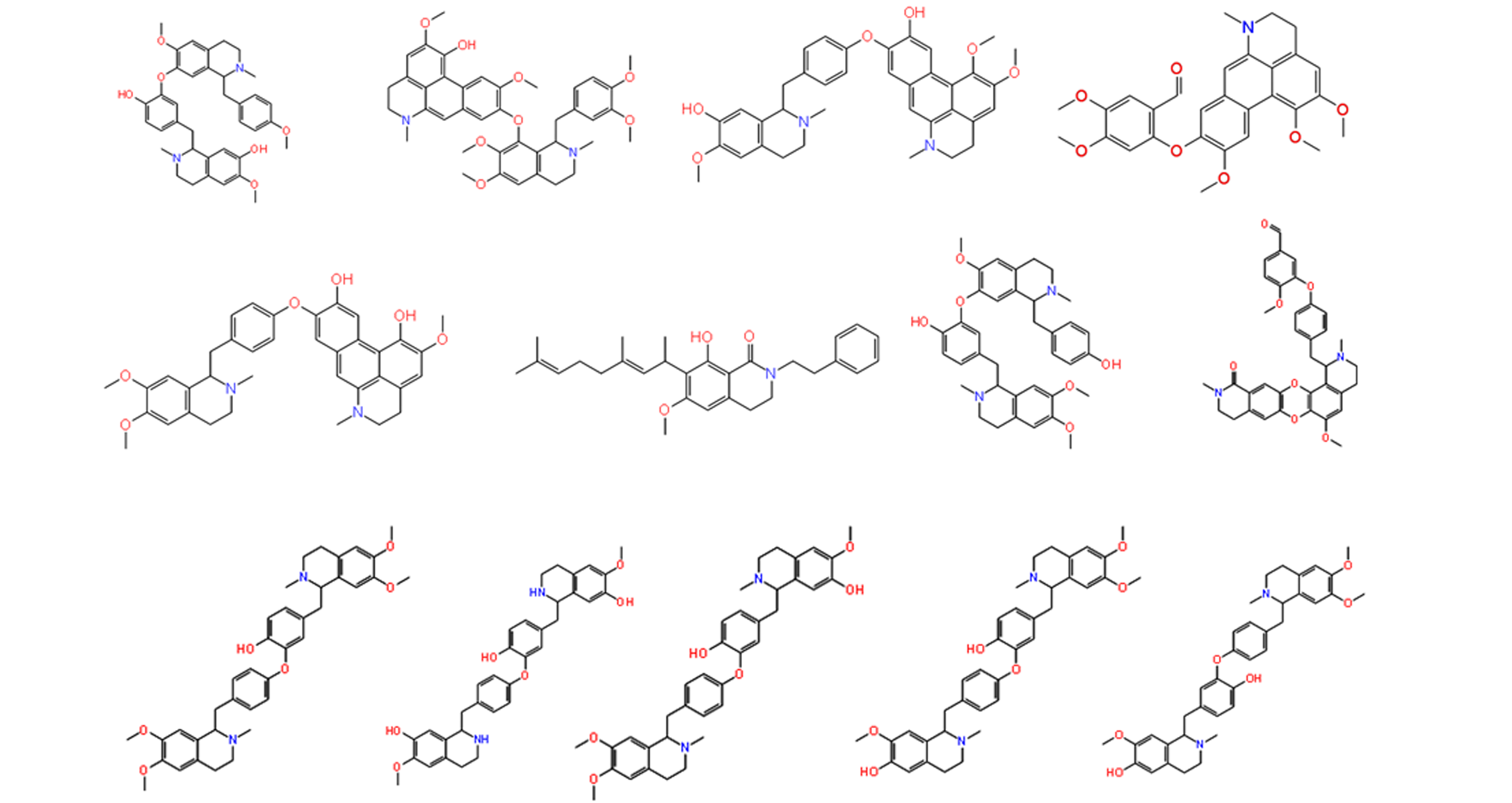
**

**Figure S3 Chemical structures of 13 alkaloid compounds for the scaffold extraction.**


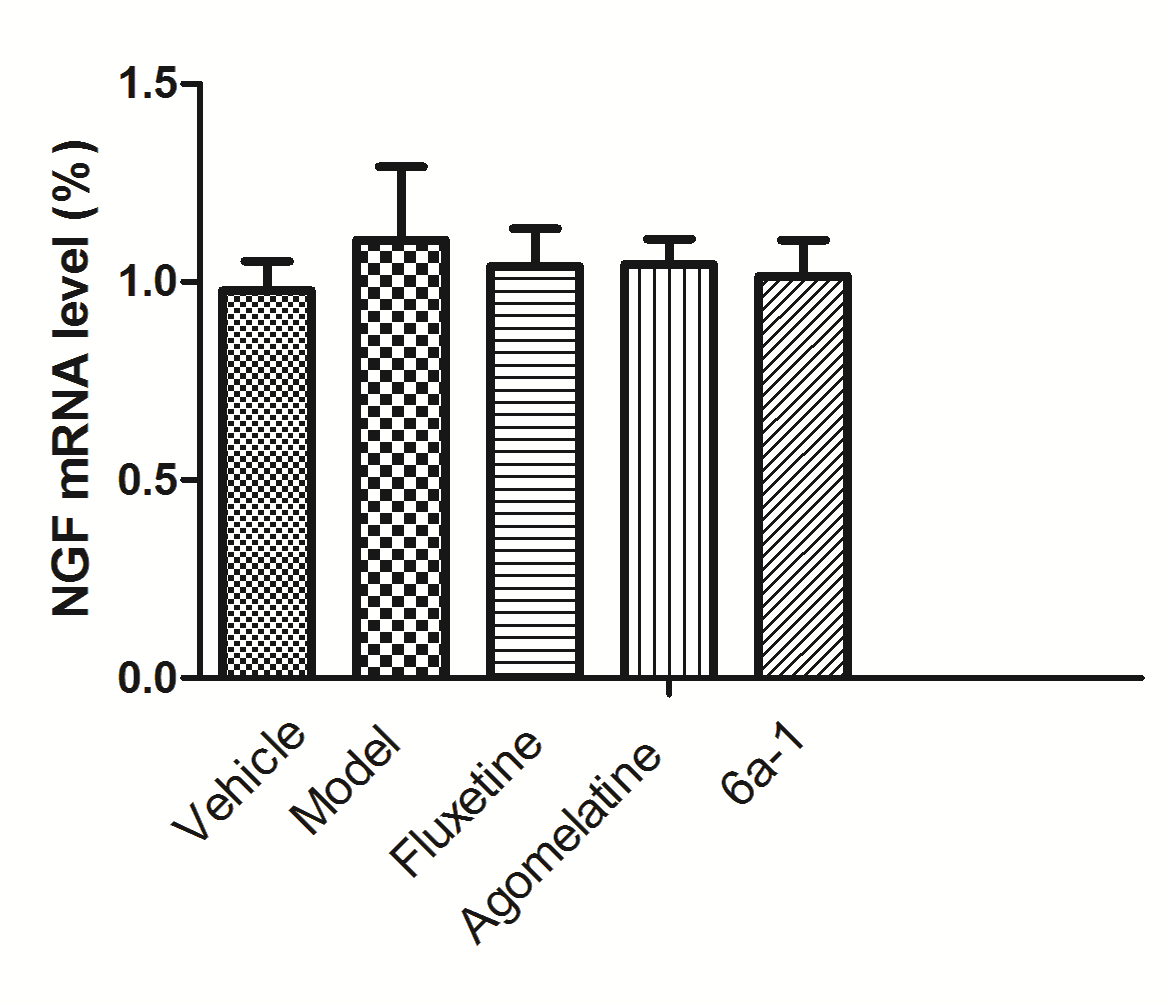


**Figure S4 Compound 6a-1 does not change the mRNA levels of NGF** **in corticosterone injured PC12 cells.** PC12 cells were treated with normal saline (conrtol); 200 μM of corticosterone (vehicle); 200 μM of corticosterone and 5 μM of drugs, respectively. Values given are the mean ± S.D. (n = 6).

**
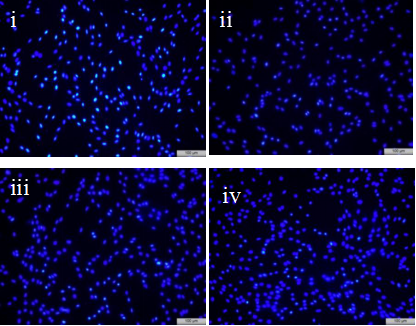
**

**Figure S5 Hoechst 33258 staining of** **Compound 6a-1 regulate the apoptosis of corticosterone injured PC12 cells.** (i) 200 μM of corticosterone; (ii) 200 μM of corticosterone and 5 μM of Fuxetine; (iii) 200 μM of corticosterone and 5 μM of Agomelatine; (iv) 200 μM of corticosterone and 5 μM of compound **6a-1**.

**
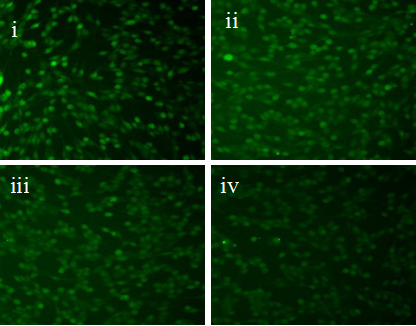
**

**Figure S6 Compound 6a-1 regulates the intracellular** **Calcium ion concentration of** **corticosterone-injured PC12 cells.** (i) 200 μM of corticosterone; (ii) 200 μM of corticosterone and 5 μM of Fuxetine; (iii) 200 μM of corticosterone and 5 μM of Agomelatine; (iv) 200 μM of corticosterone and 5 μM of compound **6a-1**.

***
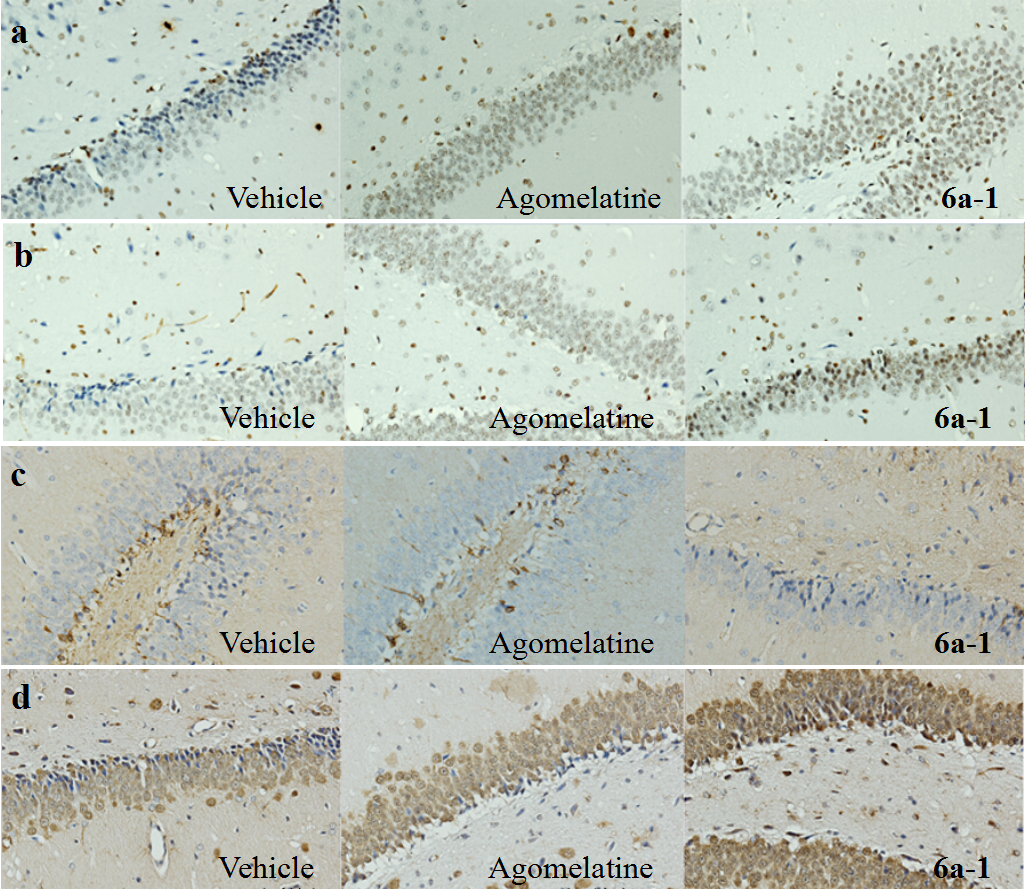
***

**Figure S7 Immunohistochemistry examination of the effect on cell survival, proliferation and maturation in rat hippocampus induced by compound 6a-1 .**

(a)PSA-NCAM staining for the immature neuron cells; (b)NeuN staining for the mature neuron cells; (c) BrdU staining for neuron cells survival; (d) BrdU staining for neuron cells proliferation.


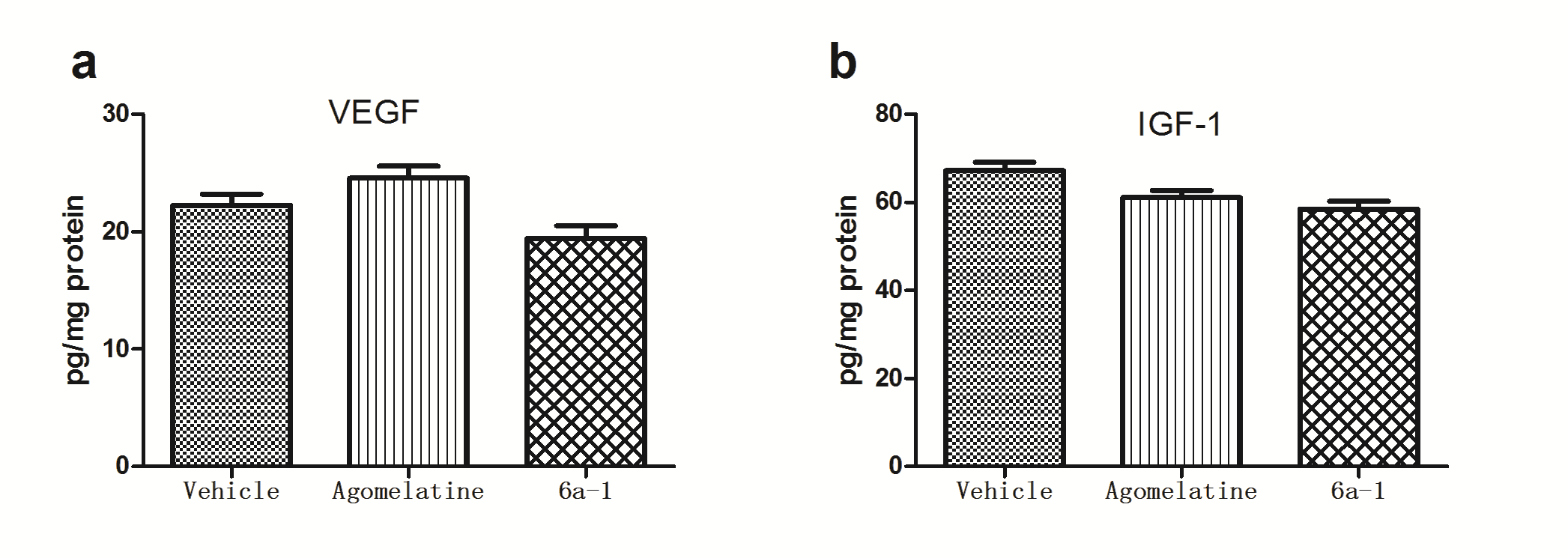


**Figure S8 Compound 6a-1 does noteffect the VEGF and IGF-1 level*in vivo*.** Results are means±SD pg/mg total protein for six rats per group.

**Table S1** The protection rates of 3,4-dihydroisoquinoline compounds on corticosterone-injured PC12 cells.

| Compd. | PR^a^(%) | Compd. | PR^a^ (%) | Compd. | PR^a^ (%) | Compd. | PR^a^ (%) |
| --- | --- | --- | --- | --- | --- | --- | --- |
|  | 1.25μM |  | 1.25μM |  | 1.25μM |  | 1.25μM |
| 6a-3 | -^b^ | 6a-18 | - | 6a-32 | - | 6b-12 | 16.3 |
| 6a-4 | - | 6a-19 | - | 6a-33 | - | 6b-13 | - |
| 6a-5 | 4.1 | 6a-20 | - | 6a-34 | - | 6b-14 | - |
| 6a-6 | - | 6a-21 | 15.2 | 6b-1 | - | 6b-15 | - |
| 6a-7 | - | 6a-22 | - | 6b-2 | - | 6b-16 | NT |
| 6a-8 | - | 6a-23 | - | 6b-3 | 10.7 | 6b-17 | 13.9 |
| 6a-10 | - | 6a-24 | - | 6b-4 | 11.9 | 6b-18 | 12.8 |
| 6a-11 | NT^c^ | 6a-25 | - | 6b-5 | - | 6b-19 | - |
| 6a-12 | 0.7 | 6a-26 | - | 6b-6 | - | 6b-20 | - |
| 6a-13 | - | 6a-27 | 10.1 | 6b-7 | - | 6b-21 | - |
| 6a-14 | - | 6a-28 | 2.4 | 6b-8 | - | 6b-22 | - |
| 6a-15 | 3.7 | 6a-29 | - | 6b-9 | NT | Ago. | 28.1 |
| 6a-16 | 6.1 | 6a-30 | - | 6b-10 | NT |  |  |
| 6a-17 | - | 6a-31 | 0.2 | 6b-11 | - |  |  |

^a^ PR represents the protection rate of the tested compounds measured at 24 h after treatment with different concentrations of compounds. PR= (A_d_-A_c_)/A_c_*100% ,whereA_c_ representsthe mean absorbance value of six independent experiments of control group only treated with corticosterone, A_d_ means the mean absorbance value of six independent experiments of test group treated with corticosterone and tested drug. ^b^ ‘-’representscompounds did not shown protection effects on corticosterone-injured PC12 cells.^c^NT represents no measure data.

**Table S2 Cytotoxicities of 3, 4-dihydroisoquinoline compounds on human embryonic kidney 293 cells and human normal liver L02 cells**

| **Compd.** | IR^a^ (%) | | **Compd.** | IR (%) | |
| --- | --- | --- | --- | --- | --- |
|  | HEK293 | L02 |  | HEK293 | L02 |
| **6a-1** | 10.3 | 13.7 | **6a-29** | 29.5 | 14.4 |
| **6a-2** | 23.4 | 12.4 | **6a-30** | 49.1 | 47.5 |
| **6a-3** | 60.1 | 59.6 | **6a-31** | 56.1 | 46.1 |
| **6a-4** | 46.2 | -^b^ | **6a-32** | 54.8 | 43.2 |
| **6a-5** | 60.0 | 67.0 | **6a-33** | 43.0 | 45.2 |
| **6a-6** | 34.1 | 24.9 | **6a-34** | 47.1 | 44.0 |
| **6a-7** | 27.4 | 23.5 | **6b-1** | 19.5 | 27.2 |
| **6a-8** | 38.4 | 60.6 | **6b-2** | 37.9 | 38.9 |
| **6a-9** | 60.1 | 52.6 | **6b-3** | 23.2 | 35.8 |
| **6a-10** | 27.6 | 22.5 | **6b-4** | 29.2 | 57.7 |
| **6a-11** | 26.3 | 21.6 | **6b-5** | 40.1 | 40.7 |
| **6a-12** | 43.8 | 19.1 | **6b-6** | 17.6 | 13.9 |
| **6a-13** | 57.6 | 43.0 | **6b-7** | 27.2 | 24.7 |
| **6a-14** | - | 35.8 | **6b-8** | 49.2 | 50.0 |
| **6a-15** | 56.1 | 49.5 | **6b-9** | 31.6 | 25.7 |
| **6a-16** | 17.4 | 13.3 | **6b-10** | 34.7 | 31.9 |
| **6a-17** | 55.8 | 42.2 | **6b-11** | 28.1 | 27.3 |
| **6a-18** | 53.3 | 63.2 | **6b-12** | 49.4 | 48.1 |
| **6a-19** | 55.9 | 45.0 | **6b-13** | 18.4 | 19.7 |
| **6a-20** | 57.1 | 54.9 | **6b-14** | 22.8 | 47.7 |
| **6a-21** | 49.8 | 58.8 | **6b-15** | 47.5 | 48.9 |
| **6a-22** | 22.2 | 31.8 | **6b-16** | 52.0 | 53.2 |
| **6a-23** | 57.8 | 66.9 | **6b-17** | 21.9 | 20.1 |
| **6a-24** | 62.9 | 55.8 | **6b-18** | 56.1 | 57.5 |
| **6a-25** | 65.7 | 50.8 | **6b-19** | 33.5 | 31.5 |
| **6a-26** | 48.6 | 42.7 | **6b-20** | 47.8 | 52.0 |
| **6a-27** | 51.8 | 45.9 | **6b-21** | 41.2 | 38.3 |
| **6a-28** | 58.5 | 43.7 | **6b-22** | 46.4 | 48.5 |
| **Ago.** | 47.5 | 41.8 |  |  |  |

^a^IR is the mean inhibitory rate calculated from three independent experiments measured at 24 h after treatment with the test compound at the concentration of 100μM. The viability of the untreated cells was regarded as 100%. ^b^‘-’ representscompounds did not shown inhibitory effects on tested cells.

**Table S3** Calculated logP (ClogP) and topological polar surface area (tPSA) values of the target compounds.

| Compd. | CLogP | tPSA | Compd. | CLogP | tPSA |
| --- | --- | --- | --- | --- | --- |
| 6a-1 | 1.3 | 50.7 | 6a-18 | 4.9 | 50.7 |
| 6a-2 | 1.8 | 50.7 | 6a-19 | 2.9 | 74.5 |
| 6a-3 | 2.4 | 50.7 | 6a-20 | 2.6 | 77.0 |
| 6a-4 | 1.9 | 50.7 | 6a-21 | 2.2 | 63.1 |
| 6a-5 | 3.9 | 50.7 | 6a-22 | 3.5 | 50.7 |
| 6a-6 | 3.2 | 50.7 | 6a-23 | 2.3 | 63.1 |
| 6a-7 | 3.1 | 50.7 | 6a-24 | 2.6 | 63.1 |
| 6a-8 | 4.3 | 50.7 | 6a-25 | 3.5 | 50.7 |
| 6a-9 | 2.2 | 59.9 | 6a-26 | 4.0 | 63.1 |
| 6a-10 | 3.0 | 50.7 | 6a-27 | 4.0 | 50.7 |
| 6a-11 | 3.9 | 50.7 | 6a-28 | 4.3 | 50.7 |
| 6a-12 | 3.4 | 50.7 | 6a-29 | 3.2 | 63.1 |
| 6a-13 | 4.5 | 50.7 | 6a-30 | 3.1 | 63.1 |
| 6a-14 | 2.05 | 84.8 | 6a-31 | 3.2 | 63.1 |
| 6a-15 | 3.2 | 69.2 | 6a-32 | 3.6 | 67.8 |
| 6a-16 | 2.9 | 67.8 | 6a-33 | 3.5 | 67.8 |
| 6a-17 | 3.0 | 62.7 | 6a-34 | 3.8 | 67.8 |
| 6b-1 | 2.3 | 62.7 | 6b-12 | 3.7 | 75.1 |
| 6b-2 | 4.2 | 62.7 | 6b-13 | 4.4 | 62.7 |
| 6b-3 | 4.2 | 62.7 | 6b-14 | 4.5 | 62.7 |
| 6b-4 | 3.8 | 62.7 | 6b-15 | 3.6 | 114.5 |
| 6b-5 | 5.6 | 62.7 | 6b-16 | 4.3 | 114.5 |
| 6b-6 | 3.2 | 86.5 | 6b-17 | 2.6 | 75.1 |
| 6b-7 | 3.6 | 86.5 | 6b-18 | 4.9 | 62.7 |
| 6b-8 | 3.3 | 101.4 | 6b-19 | 3.6 | 114.5 |
| 6b-9 | 5.5 | 62.7 | 6b-20 | 3.8 | 62.7 |
| 6b-10 | 2.5 | 62.7 | 6b-21 | 4.4 | 62.7 |
| 6b-11 | 2.6 | 75.1 | 6b-22 | 3.1 | 75.1 |
| Flu. | 4.6 | 21.3 | Ago. | 2.1 | 38.3 |

**Table S4** IC_50_values of the compound **6a-1** on H9C2 cells and hERG K^+^channel.

| Compd | IC_50_(μM) | |
| --- | --- | --- |
|  | H9C2 | hERG K^+^channel^a^ |
| **6a-1** | 455 ± 13.3 | > 40 |
| Agomelatine | 374.7 ± 9.8 | - |
| Cisapride | - | 0.13 |
| Adriamycin | 0.16±0.02 | - |

*^a^* Measured in hERG-expressing CHO cells using Qpatch 16X assay.

**Table S5** FLIPR assays of compound **6a-1** on 5-HT_2C_ receptor antagonism effects.

| Compound | IC_50_ (μM) | Inhibition^a^（%） |
| --- | --- | --- |
| **6a-1** | >100 | 5.2 |
| **Agomelatine** | 8.51 | 66.6 |
| **Methysergide** | 0.025 | 101.0 |

^a^ Inhibition of the max tested concentration at 100μM.

# ^1^HNMR, ^13^C NMR and MS spectra of target compounds

#
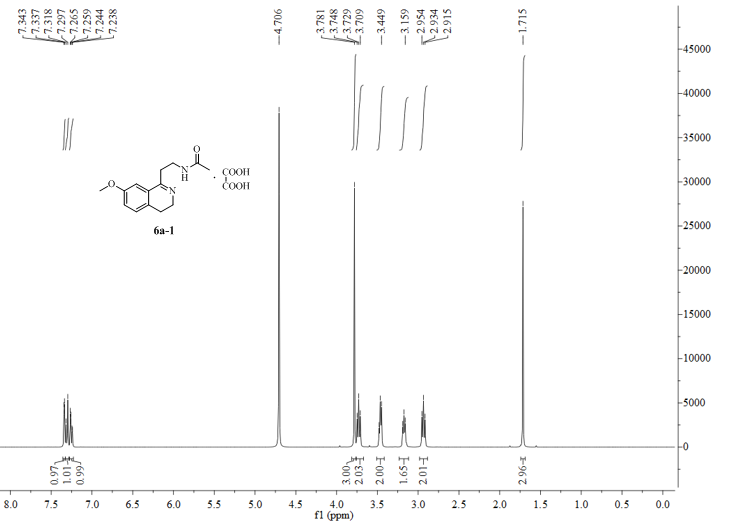

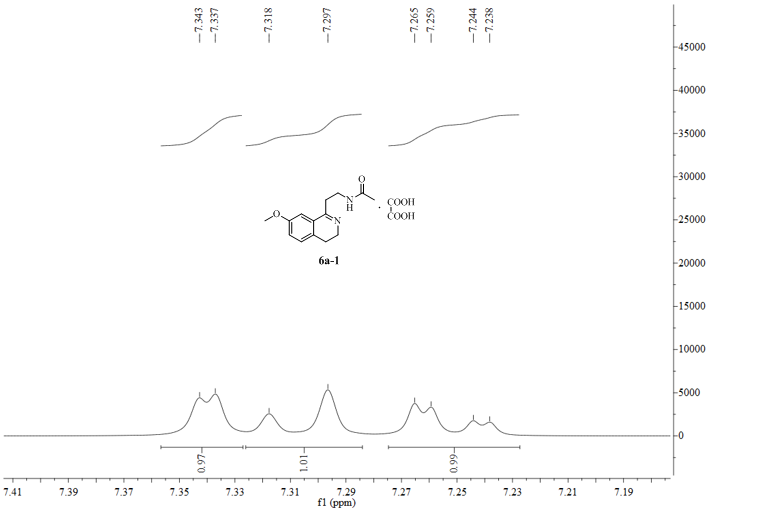

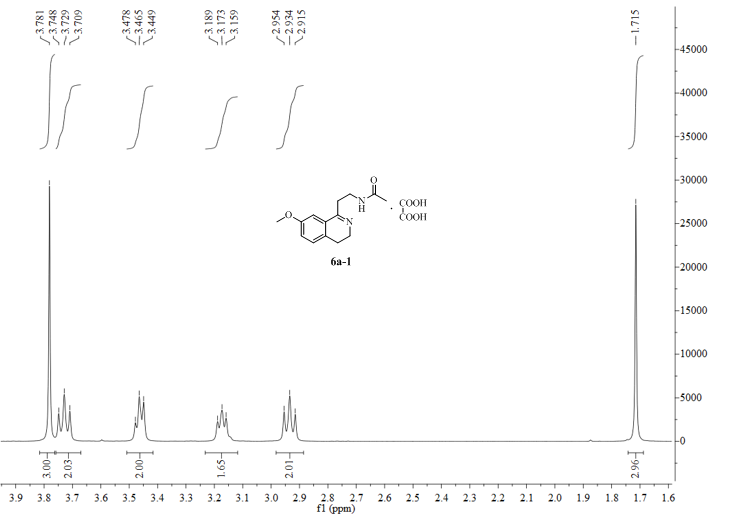


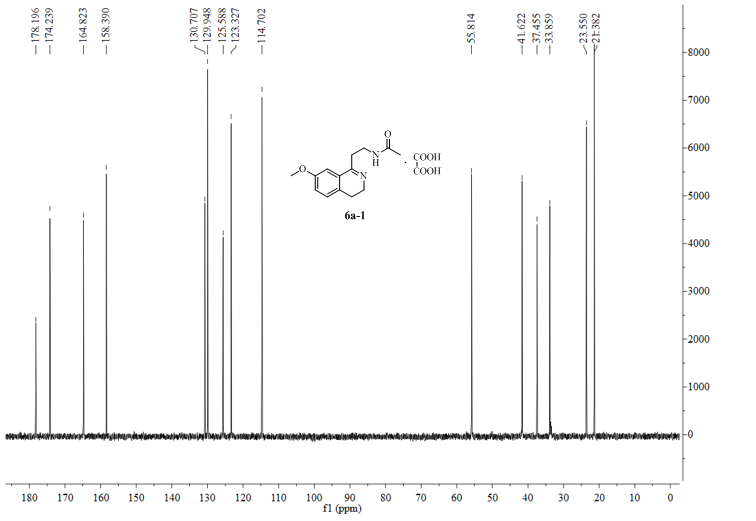


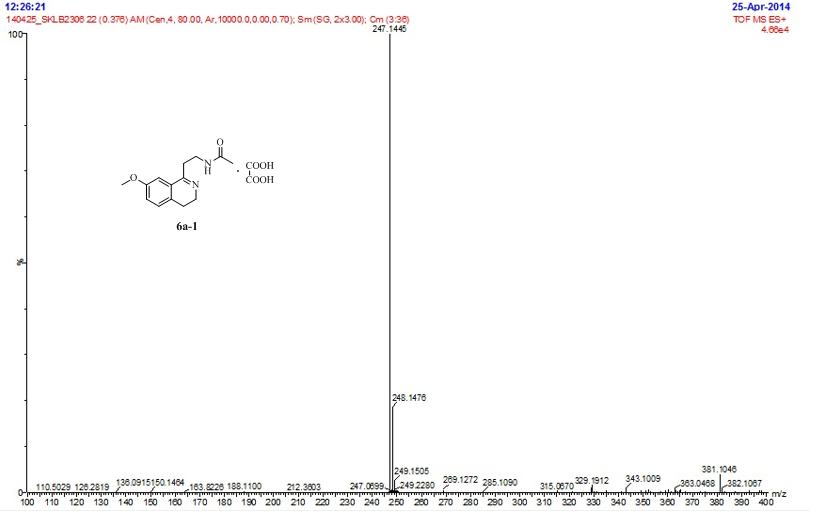


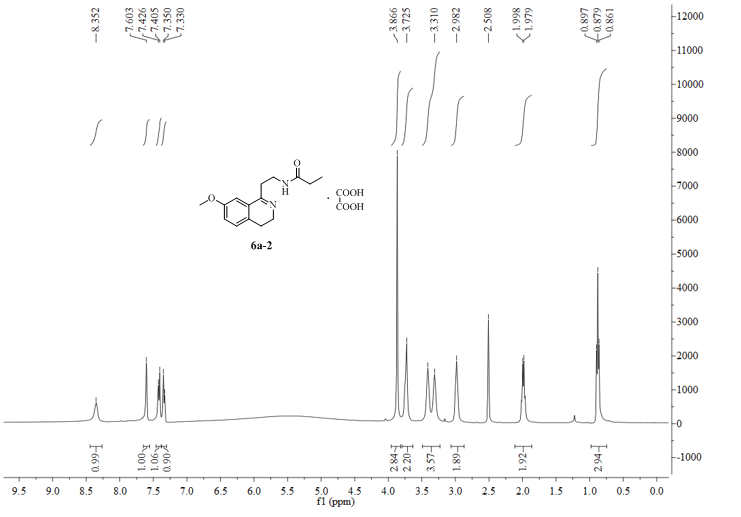


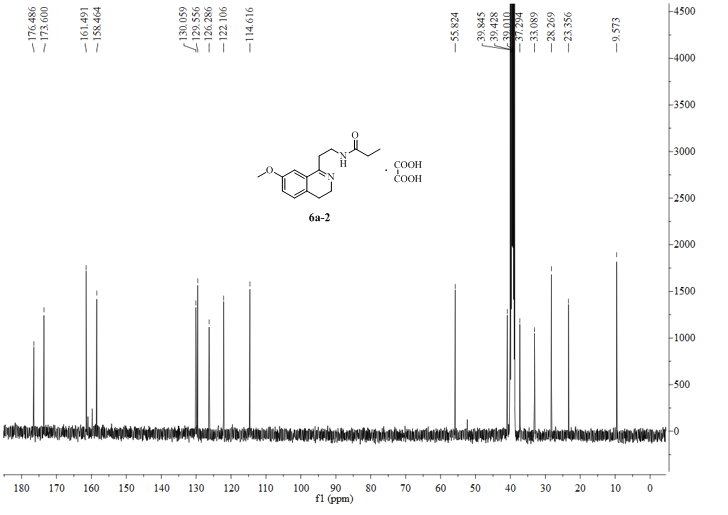

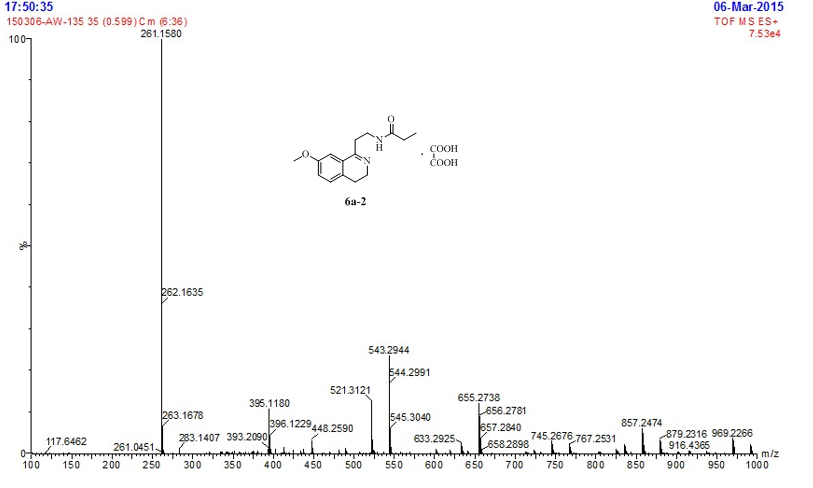

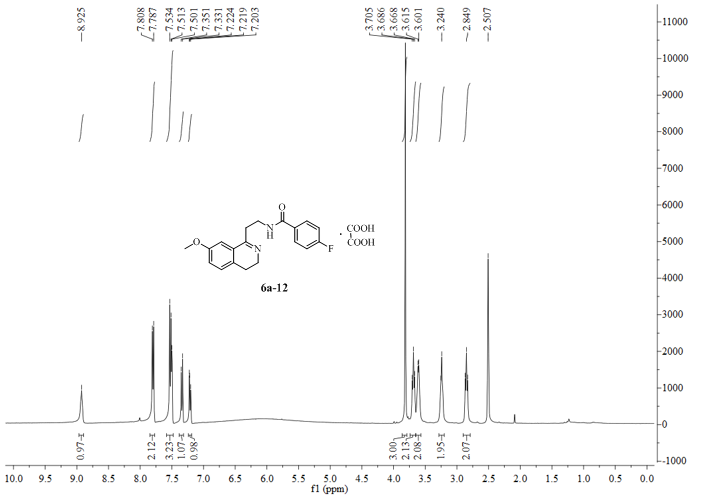

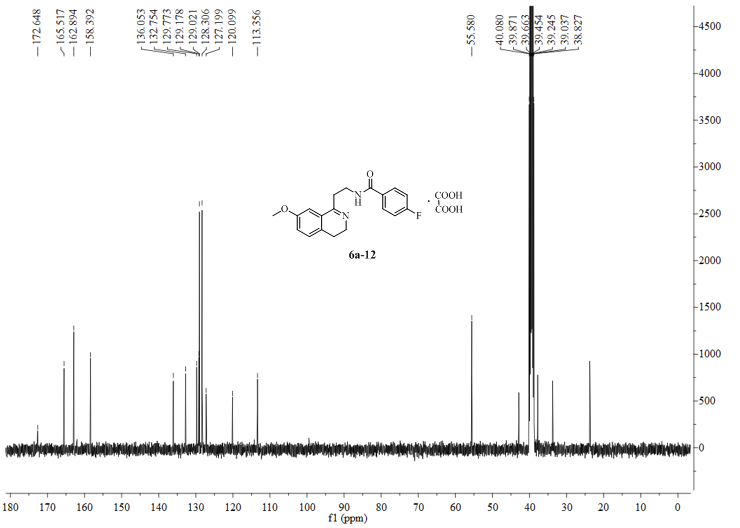

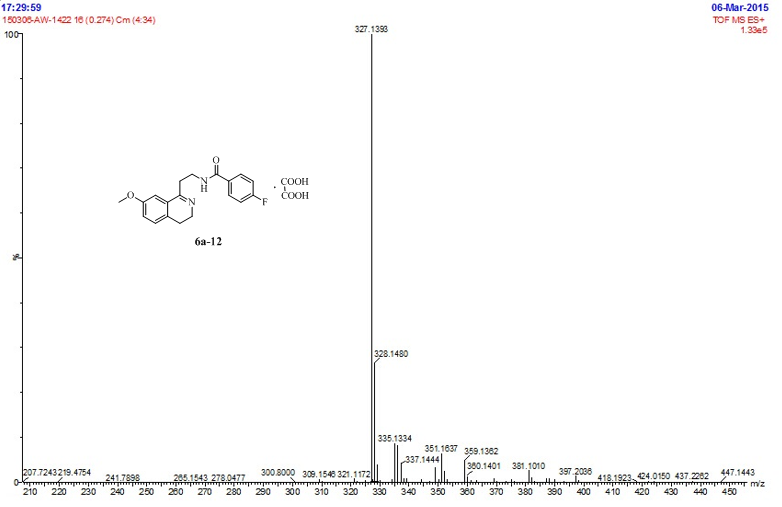

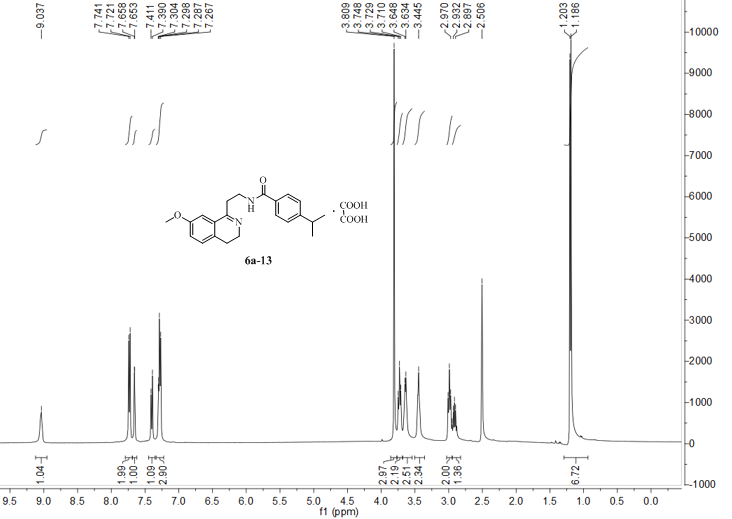

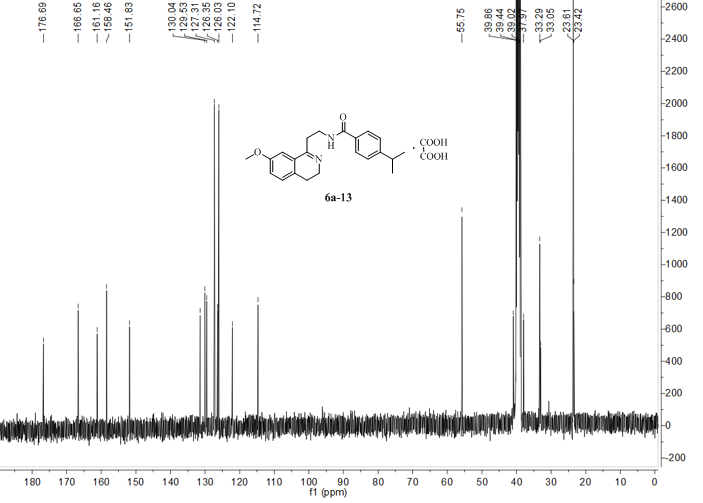

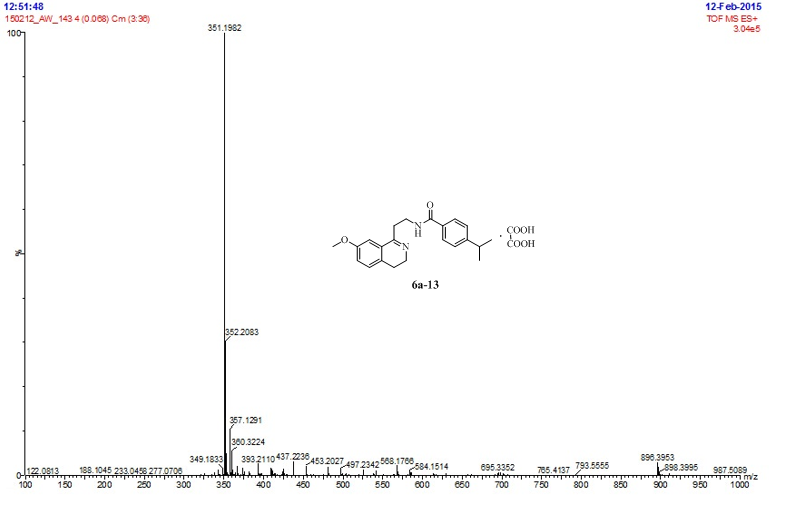


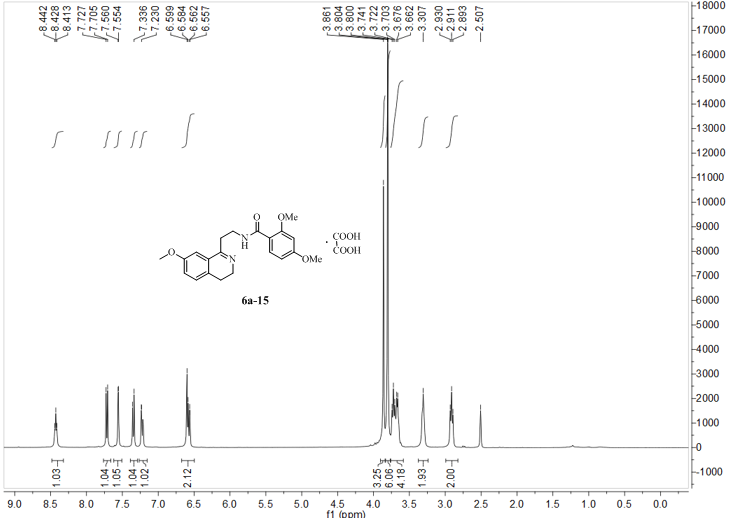

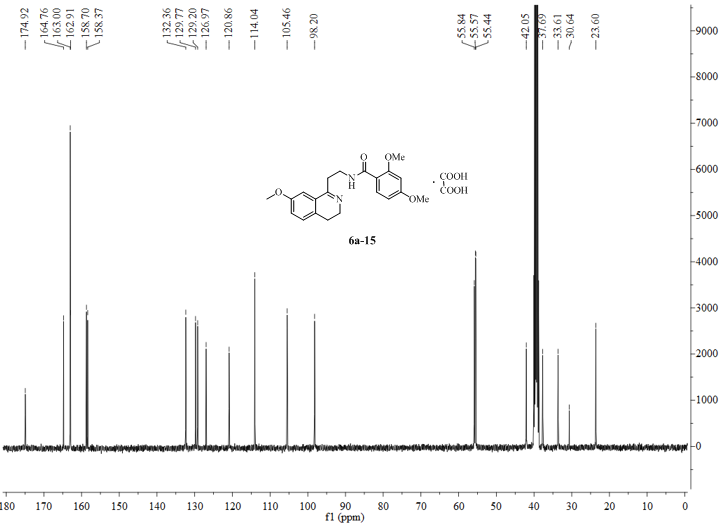

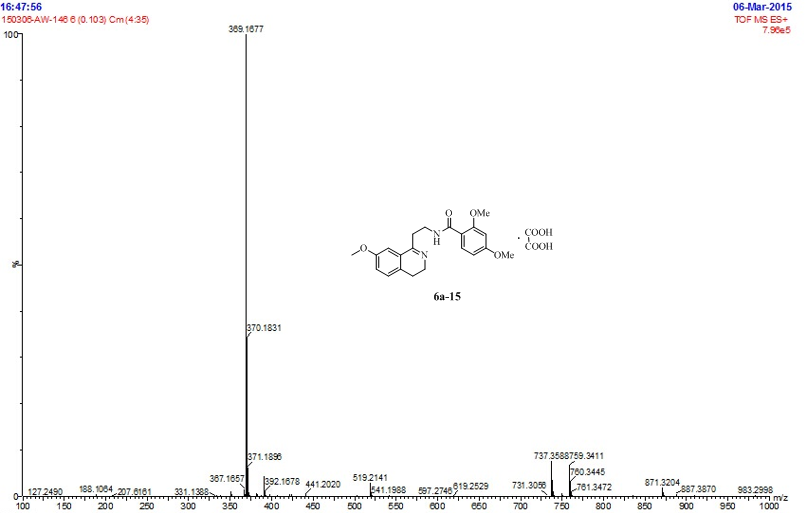

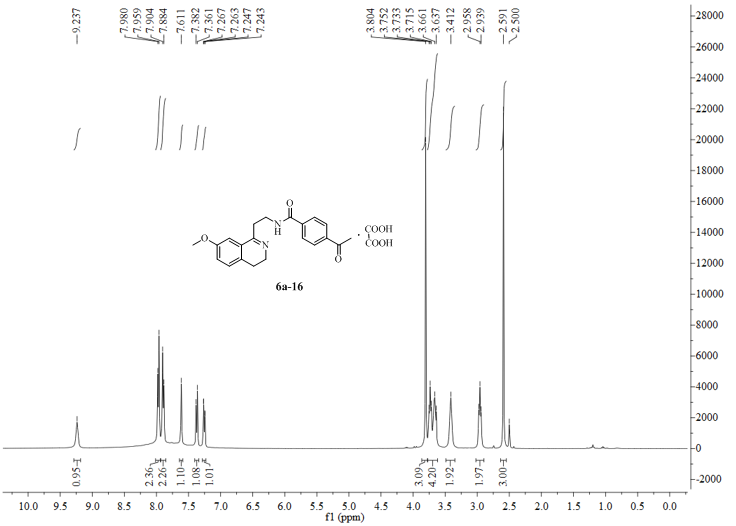

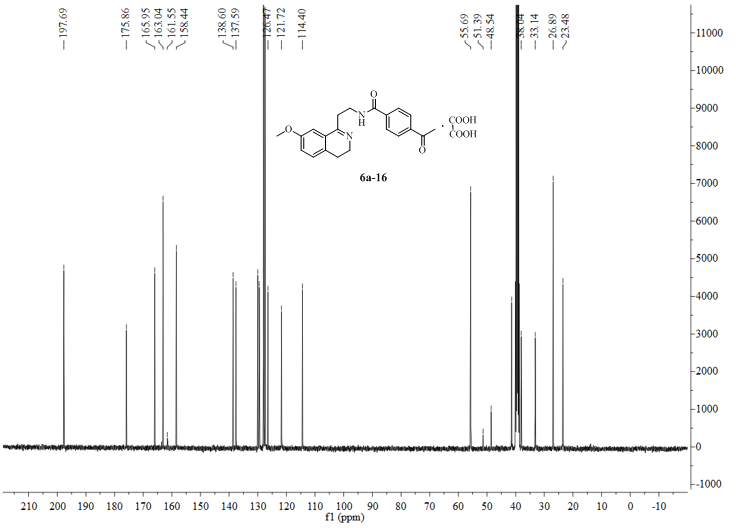

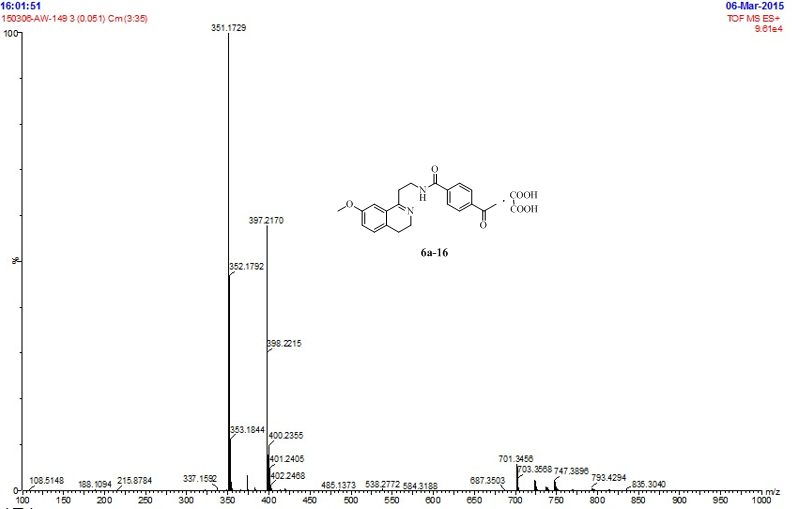

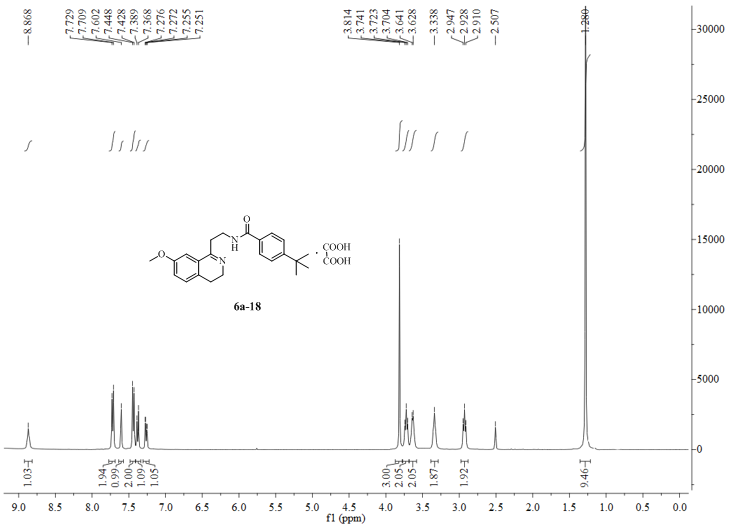

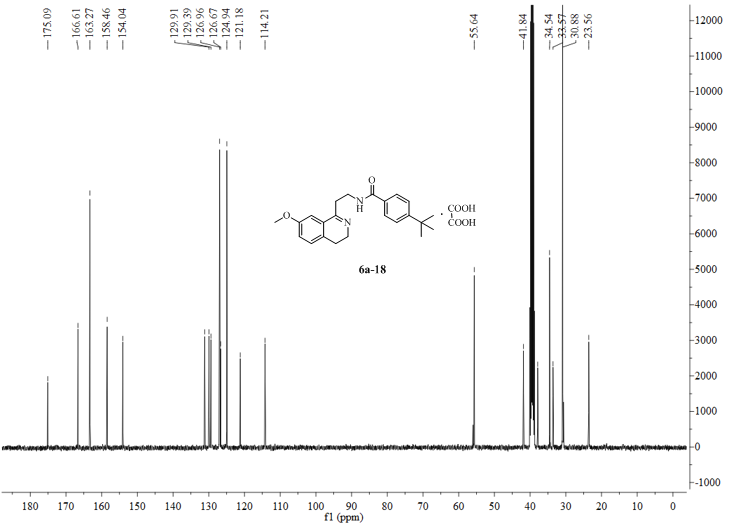

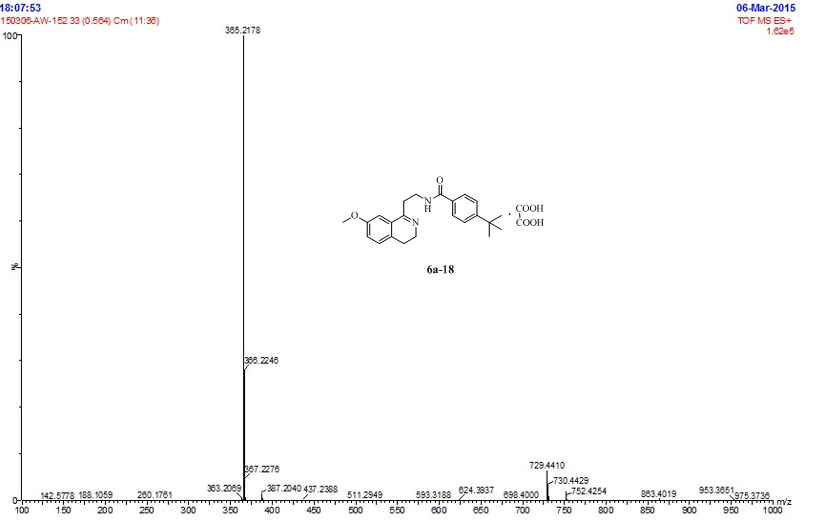

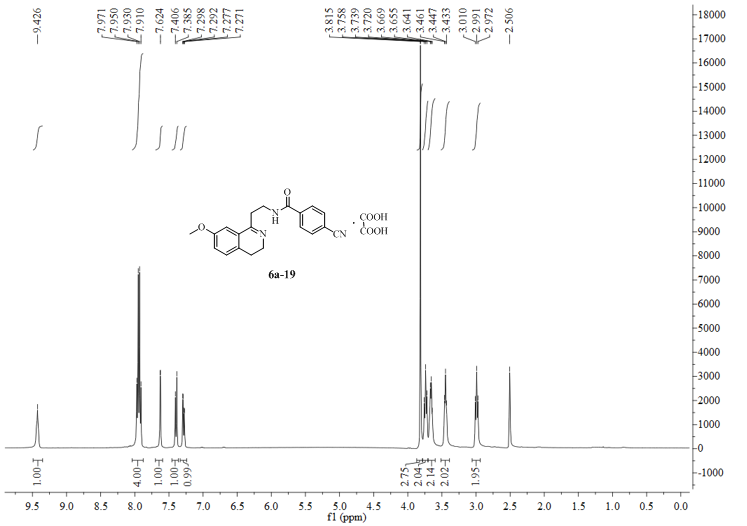

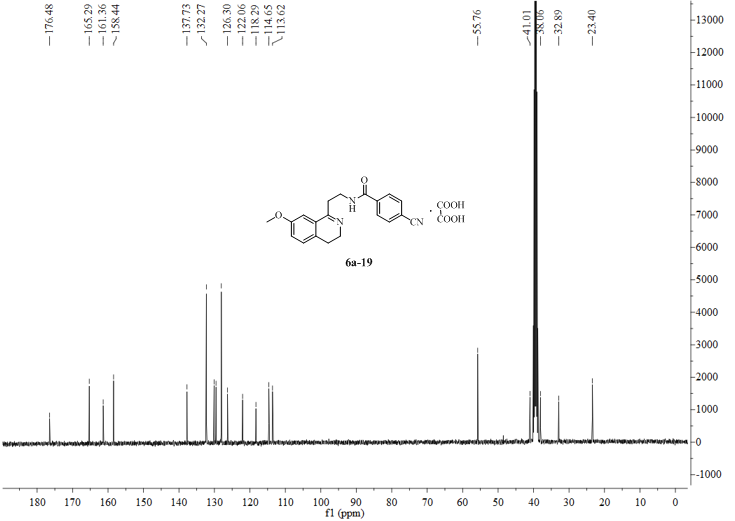

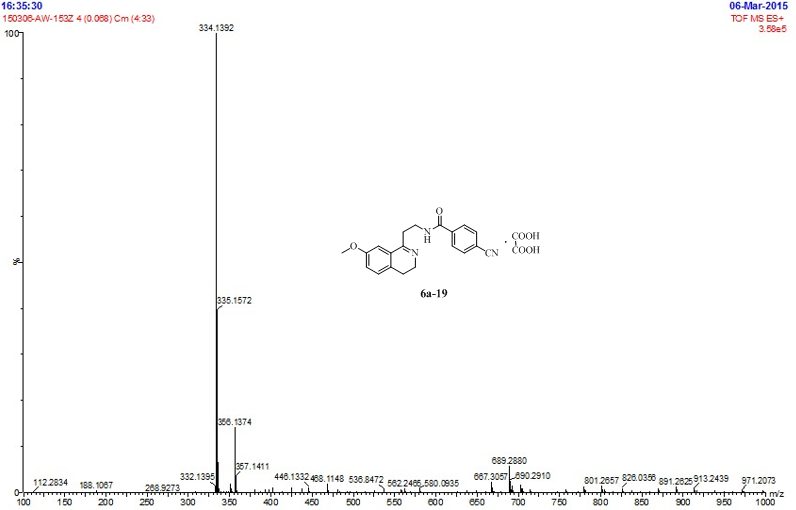

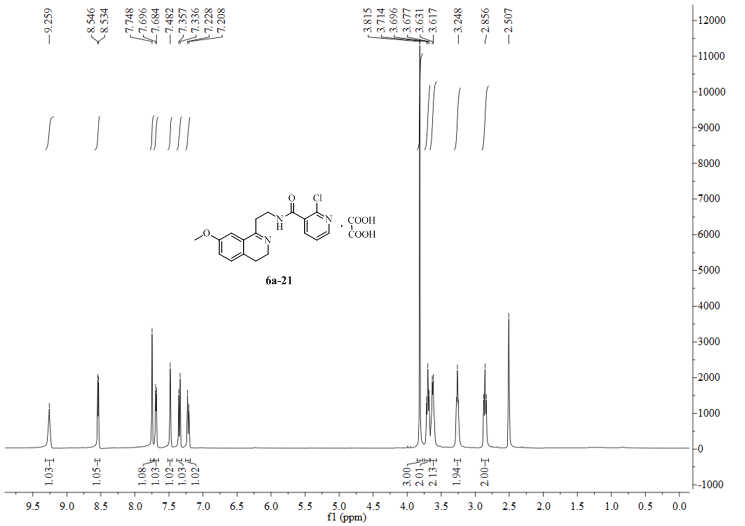

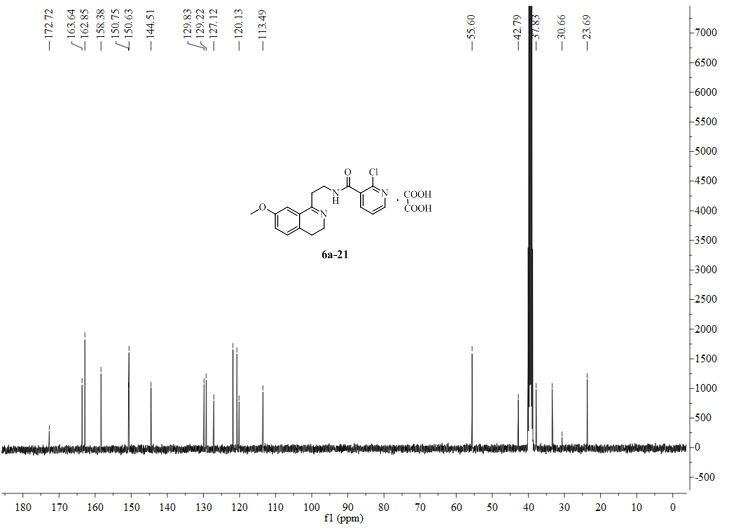

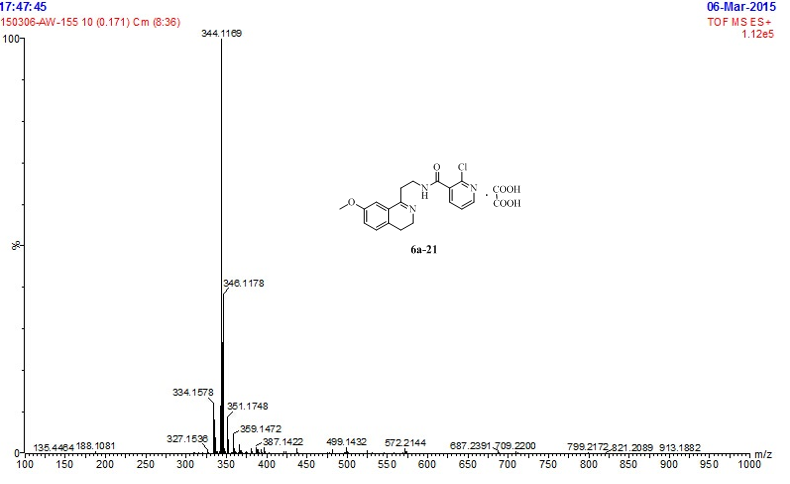

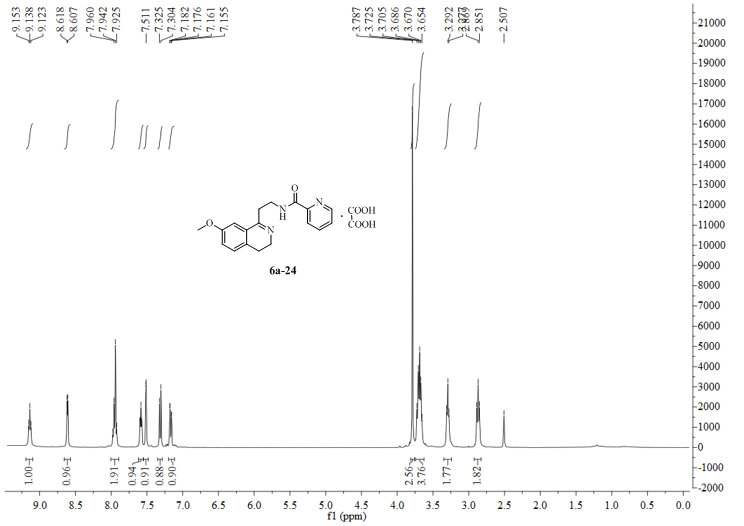

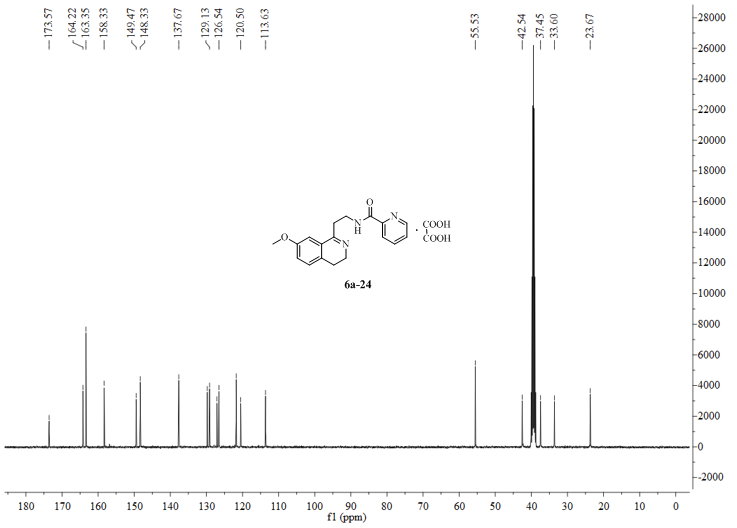

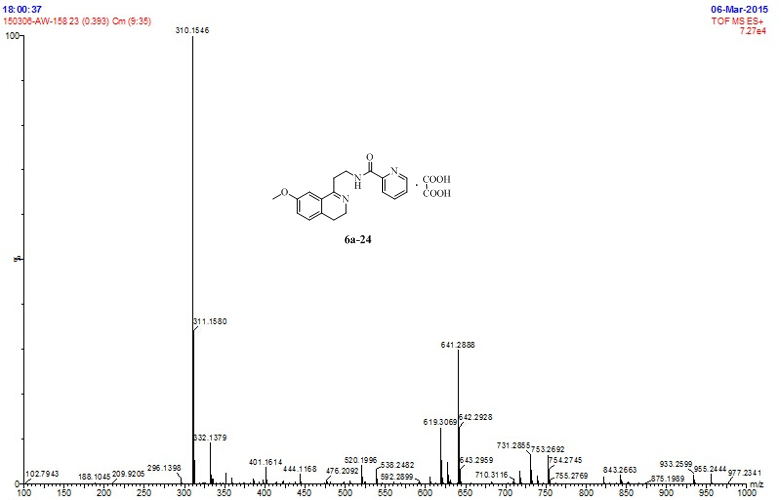

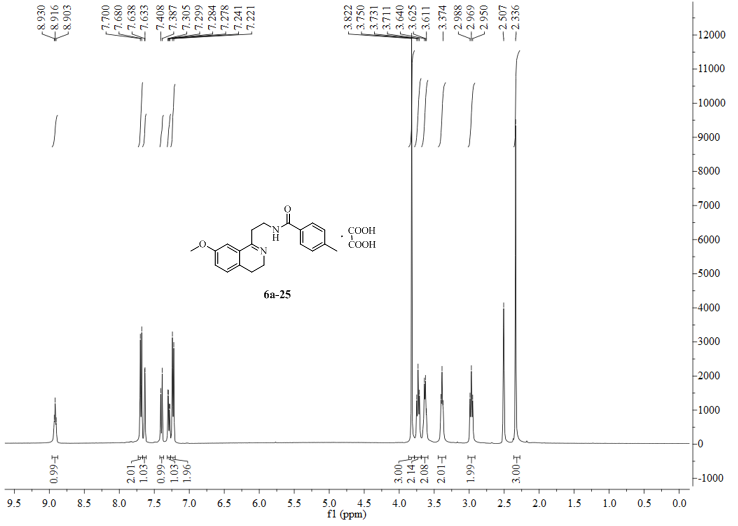


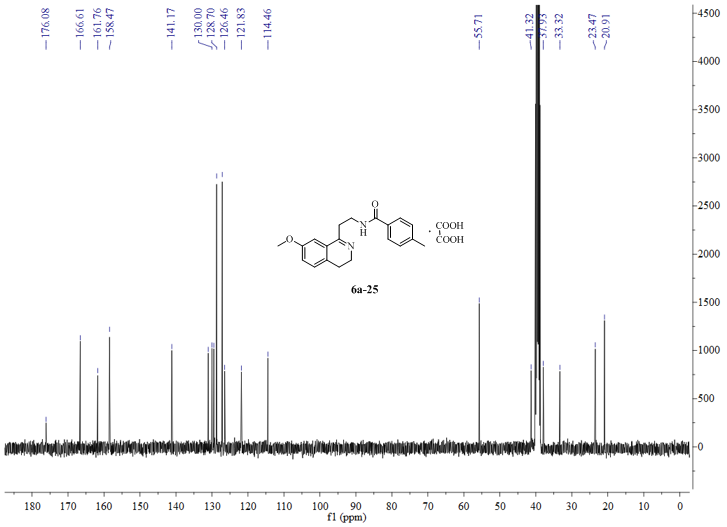


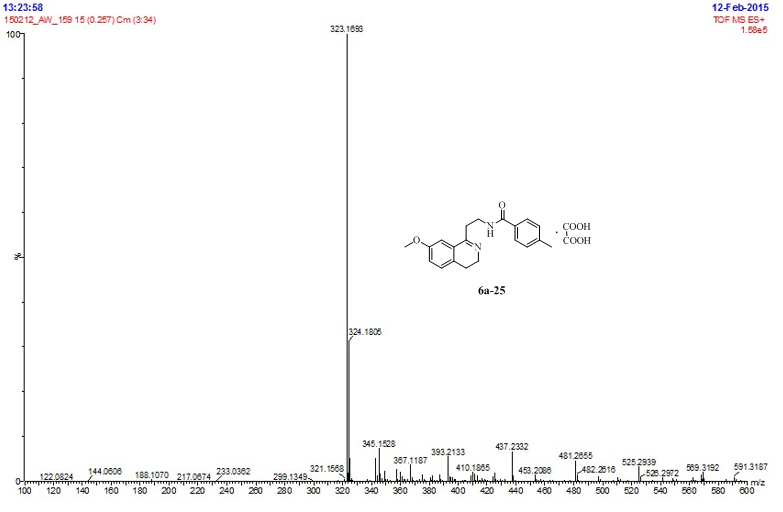

Supplement: Supplementary Information [file srep34711-s1.doc]
